# Supplementary material for: Signatures of Transcription Factor Evolution and the Secondary Gain of Red Algae Complexity
Source: Genes (Basel). 2021 Jul 9;12(7):1055. doi: 10.3390/genes12071055 (PMC8304369; doi:10.3390/genes12071055)
Supplement: Supplementary file 1 [file Figure S1+S2.pdf › Figure S1+S2.pdf]

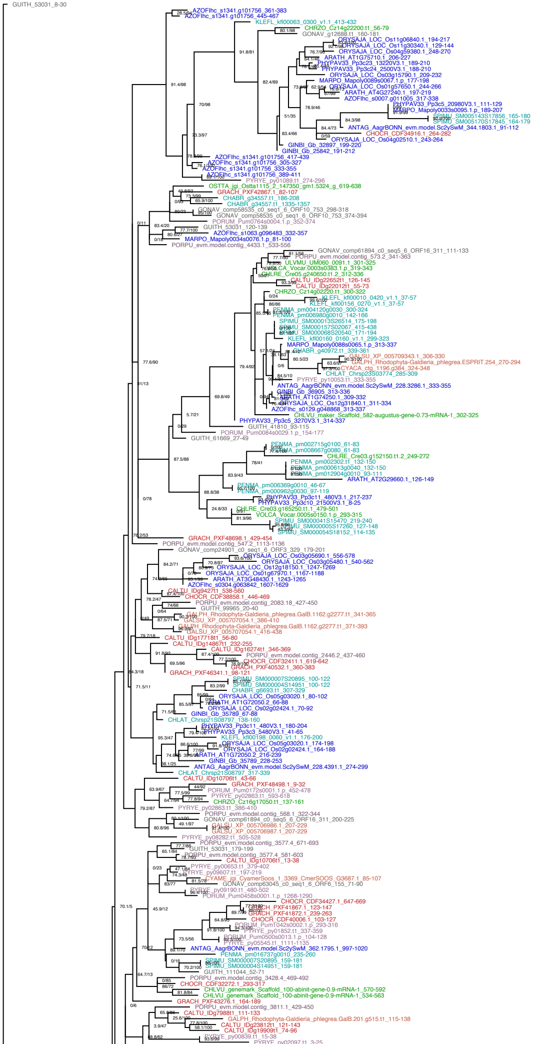

Figure S1. Maximum likelihood tree of the C2H2 family.

The outgroup-rooted tree was calculated using the model VT+R8. The tree contains sequences from 30 species (Table S1). The names of the species are abbreviated using a five-letter code where the first three letters indicate the species and the final two the genus. The species names are color-coded according to the taxonomic groups: Cyanidiales are shown in light red, Florideophyceae in dark red, Porphyridiophyceae in dark purple, Bangiophyceae in light purple, Chlorophyta in green, streptophyte algae in turquoise, Embryophytes in dark blue, and Cryptophytes (outgroup) in gray. The numbers at the nodes indicate bootstrap values. Distinct groups of the Phragmoplastophyta were collapsed for clarity.

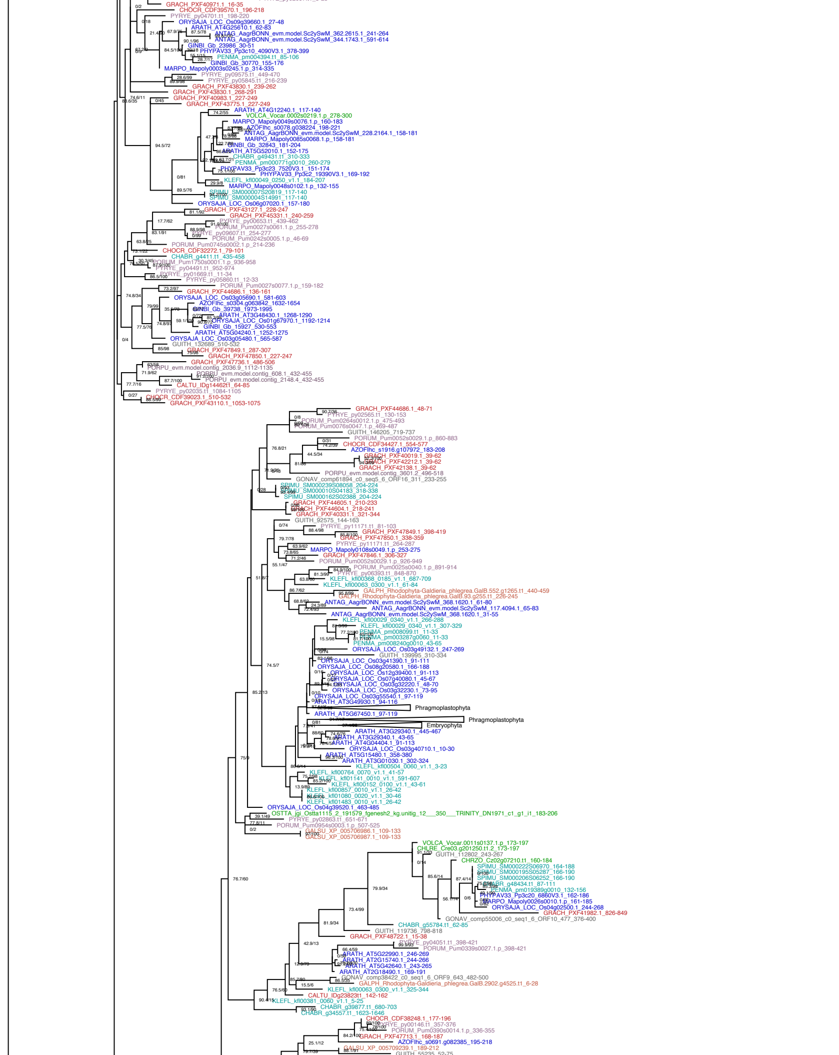

GRACH_PXF40971.1_16-35
CHOCR_CDF39570.1_196-218
PYRYE_py04701.t1_198-220
ORYSAJA_LOC_Os09g39660.1_27-48
ARATH_AT4G25610.1_62-83
ANTAG_AagrBONN_evm.model.Sc2ySwM_362.2615.1_241-264
ANTAG_AagrBONN_evm.model.Sc2ySwM_344.1743.1_591-614
GINBI_Gb_23986_30-51
PHYPAV33_Pp3c10_4090V3.1_378-399
PENMA_pm004394.t1_85-106
GINBI_Gb_30770_155-176
MARPO_Mapoly0003s0245.1.p_314-335
PYRYE_py09575.t1_449-470
PYRYE_py05845.t1_216-239
GRACH_PXF43830.1_239-262
GRACH_PXF43830.1_268-291
GRACH_PXF40983.1_227-249
GRACH_PXF43775.1_227-249
ARATH_AT4G12240.1_117-140
VOLCA_Vocar.0002s0219.1.p_278-300
MARPO_Mapoly0049s0076.1.p_160-183
AZOFIhc_s0078.g038224_198-221
ANTAG_AagrBONN_evm.model.Sc2ySwM_228.2164.1_158-181
MARPO_Mapoly0085s0068.1.p_158-181
GINBI_Gb_32843_181-204
ARATH_AT5G52010.1_152-175
CHABR_g49431.t1_310-333
PENMA_pm000771g0010_260-279
PHYPAV33_Pp3c23_7520V3.1_151-174
PHYPAV33_Pp3c2_19390V3.1_169-192
KLEFL_kfl00049_0250_v1.1_184-207
MARPO_Mapoly0048s0102.1.p_132-155
SPIMU_SM000007S20819_117-140
SPIMU_SM000004S14991_117-140
ORYSAJA_LOC_Os06g07020.1_157-180
GRACH_PXF43127.1_228-247
GRACH_PXF45331.1_240-259
PYRYE_py00653.t1_439-462
PORUM_Pum0027s0061.1.p_255-278
PYRYE_py09607.t1_254-277
PORUM_Pum0242s0005.1.p_46-69
PORUM_Pum0745s0002.1.p_214-236
CHOCR_CDF32272.1_79-101
CHABR_g4411.t1_435-458
PORUM_Pum1750s0001.1.p_936-958
PYRYE_py04491.t1_952-974
PYRYE_py01669.t1_11-34
PYRYE_py05860.t1_12-33
PORUM_Pum0027s0077.1.p_159-182
GRACH_PXF44686.1_136-161
ORYSAJA_LOC_Os03g05690.1_581-603
AZOFIhc_s0304.g063842_1632-1654
GINBI_Gb_39738_1973-1995
ARATH_AT3G48430.1_1268-1290
ORYSAJA_LOC_Os01g67970.1_1192-1214
GINBI_Gb_15927_530-553
ARATH_AT5G04240.1_1252-1275
ORYSAJA_LOC_Os03g05480.1_565-587
GUITH_132689_510-532
GRACH_PXF47849.1_287-307
GRACH_PXF47850.1_227-247
GRACH_PXF47736.1_486-506
PORPU_evm.model.contig_2036.9_1112-1135
PORPU_evm.model.contig_608.1_432-455
PORPU_evm.model.contig_2148.4_432-455
CALTU_IDg14462t1_64-85
PYRYE_py02035.t1_1084-1105
CHOCR_CDF39023.1_510-532
GRACH_PXF43110.1_1053-1075
GRACH_PXF44686.1_48-71
PYRYE_py02565.t1_130-153
PORUM_Pum0264s0012.1.p_475-493
PORUM_Pum0076s0047.1.p_469-487
GUITH_146205_719-737
PORUM_Pum0052s0029.1.p_860-883
CHOCR_CDF34427.1_554-577
AZOFIhc_s1916.g107972_183-208
GRACH_PXF40019.1_39-62
GRACH_PXF42212.1_39-62
GRACH_PXF42138.1_39-62
PORPU_evm.model.contig_3601.2_496-518
GONAV_comp61894_c0_seq5_6_ORF16_311_233-255
SPIMU_SM000239S08058_204-224
SPIMU_SM000010S04183_318-338
SPIMU_SM000162S02388_204-224
GRACH_PXF44605.1_210-233
GRACH_PXF44604.1_218-241
GRACH_PXF40331.1_321-344
GUITH_92575_144-163
PYRYE_py11171.t1_81-103
GRACH_PXF47849.1_398-419
GRACH_PXF47850.1_338-359
PYRYE_py11171.t1_264-287
MARPO_Mapoly0108s0049.1.p_253-275
GRACH_PXF47846.1_306-327
PORUM_Pum0052s0029.1.p_926-949
PORUM_Pum0025s0040.1.p_891-914
PYRYE_py06393.t1_848-870
KLEFL_kfl00368_0185_v1.1_687-709
KLEFL_kfl00063_0300_v1.1_61-84
GALPH_Rhodophyta-Galdieria_phlegrea.GalB.552.g1265.t1_440-459
GALPH_Rhodophyta-Galdieria_phlegrea.GalB.93.g255.t1_226-245
ANTAG_AagrBONN_evm.model.Sc2ySwM_368.1620.1_61-80
ANTAG_AagrBONN_evm.model.Sc2ySwM_117.4094.1_65-83
ANTAG_AagrBONN_evm.model.Sc2ySwM_368.1620.1_31-55
KLEFL_kfl00029_0340_v1.1_266-288
KLEFL_kfl00029_0340_v1.1_307-329
PENMA_pm008099.t1_11-33
PENMA_pm003287g0060_11-33
PENMA_pm008240g0010_43-65
ORYSAJA_LOC_Os03g49132.1_247-269
GUITH_139995_310-334
ORYSAJA_LOC_Os03g41390.1_91-111
ORYSAJA_LOC_Os08g20580.1_166-188
ORYSAJA_LOC_Os12g39400.1_91-113
ORYSAJA_LOC_Os07g40080.1_45-67
ORYSAJA_LOC_Os03g32220.1_48-70
ORYSAJA_LOC_Os03g32230.1_73-95
ORYSAJA_LOC_Os03g55540.1_97-119
ARATH_AT3G49930.1_94-116
Phragmoplastophyta
ARATH_AT5G67450.1_97-119
Phragmoplastophyta
Embryophyta
ARATH_AT3G29340.1_445-467
ARATH_AT3G29340.1_43-65
ARATH_AT4G04404.1_91-113
ORYSAJA_LOC_Os03g40710.1_10-30
ARATH_AT5G15480.1_358-380
ARATH_AT3G01030.1_302-324
KLEFL_kfl00504_0060_v1.1_3-23
KLEFL_kfl00764_0070_v1.1_41-57
KLEFL_kfl01141_0010_v1.1_591-607
KLEFL_kfl00152_0100_v1.1_43-61
KLEFL_kfl00857_0010_v1.1_26-42
KLEFL_kfl01080_0020_v1.1_30-46
KLEFL_kfl01483_0010_v1.1_26-42
ORYSAJA_LOC_Os04g39520.1_463-485
OSTTA_jgi_Ostta1115_2_191579_fgenesh2_kg.unitig_12___350___TRINITY_DN1971_c1_g1_i1_183-206
PYRYE_py02863.t1_651-671
PORUM_Pum0954s0003.1.p_507-525
GALSU_XP_005706986.1_109-133
GALSU_XP_005706987.1_109-133
VOLCA_Vocar.0011s0137.1.p_173-197
CHLRE_Cre03.g201250.t1.2_173-197
GUITH_112802_243-267
CHRZO_Cz02g07210.t1_160-184
SPIMU_SM000222S06970_164-188
SPIMU_SM000195S05287_166-190
SPIMU_SM000206S06252_166-190
CHABR_g48434.t1_87-111
PENMA_pm019389g0010_132-156
PHYPAV33_Pp3c20_6860V3.1_162-186
MARPO_Mapoly0026s0010.1.p_161-185
ORYSAJA_LOC_Os04g02500.1_244-268
GRACH_PXF41982.1_826-849
GONAV_comp55006_c0_seq1_6_ORF10_477_376-400
CHABR_g55784.t1_62-85
GUITH_119736_798-818
GRACH_PXF48722.1_15-38
PYRYE_py04051.t1_398-421
PORUM_Pum0339s0027.1.p_398-421
ARATH_AT5G22990.1_246-269
ARATH_AT2G15740.1_244-266
ARATH_AT5G42640.1_243-265
ARATH_AT2G18490.1_169-191
GONAV_comp38422_c0_seq1_6_ORF9_643_482-500
GALPH_Rhodophyta-Galdieria_phlegrea.GalB.2902.g4525.t1_6-28
KLEFL_kfl00063_0300_v1.1_325-344
CALTU_IDg23823t1_142-162
KLEFL_kfl00381_0060_v1.1_5-25
CHABR_g39877.t1_680-703
CHABR_g34557.t1_1623-1646
CHOCR_CDF38248.1_177-196
PYRYE_py00146.t1_357-376
PORUM_Pum0390s0014.1.p_336-355
GRACH_PXF47713.1_168-187
AZOFIhc_s0691.g082385_195-218
GALSU_XP_005709239.1_189-212
GUITH_55235_52-75

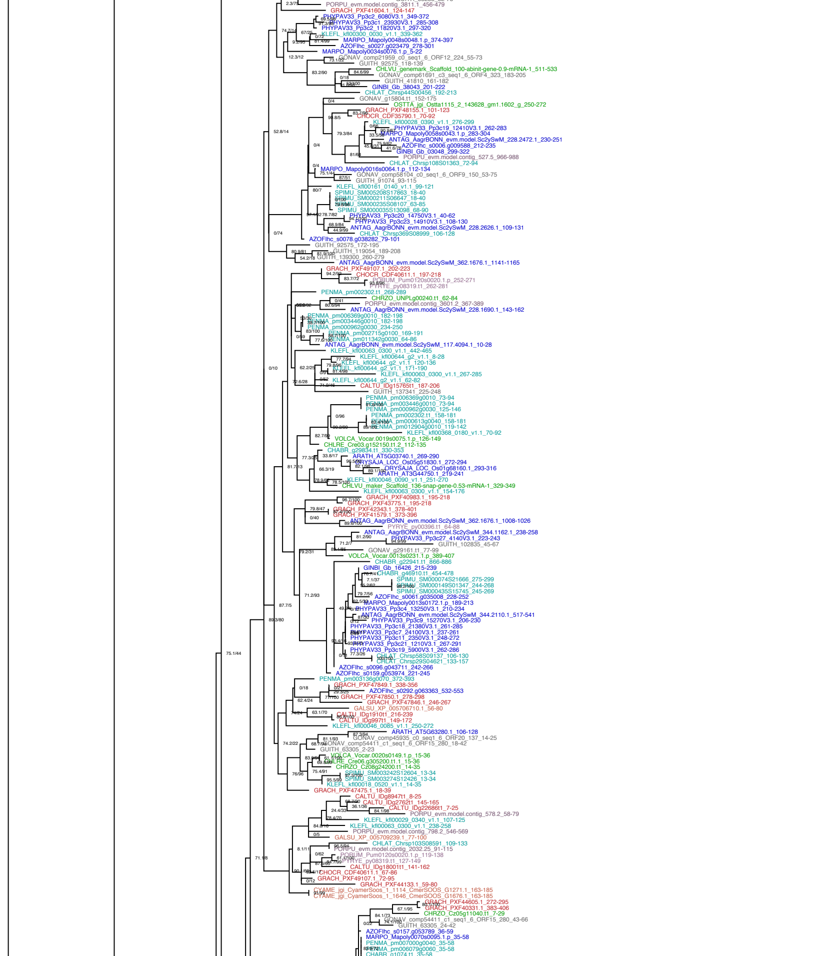
PORPU_evm.model.contig_3811.1_456-479
GRACH_PXF41604.1_124-147
PHYPAV33_Pp3c2_6080V3.1_349-372
PHYPAV33_Pp3c1_23930V3.1_285-308
PHYPAV33_Pp3c2_11820V3.1_297-320
KLEFL_kfl00300_0030_v1.1_339-362
MARPO_Mapoly0048s0048.1.p_374-397
AZOFIhc_s0027.g023479_278-301
MARPO_Mapoly0034s0076.1.p_5-22
GONAV_comp21959_c0_seq1_6_ORF12_224_55-73
GUITH_92575_118-139
CHLVU_genemark_Scaffold_100-abinit-gene-0.9-mRNA-1_511-533
GONAV_comp61691_c3_seq1_6_ORF4_323_183-205
GUITH_41810_161-182
GINBI_Gb_38043_201-222
CHLAT_Chrsp44S00456_192-213
GONAV_g15804.t1_152-175
OSTTA_jgi_Ostta1115_2_143628_gm1.1602_g_250-272
GRACH_PXF48155.1_101-123
CHOCR_CDF35790.1_70-92
KLEFL_kfl00028_0390_v1.1_276-299
PHYPAV33_Pp3c19_12410V3.1_262-283
MARPO_Mapoly0058s0043.1.p_283-304
ANTAG_AagrBONN_evm.model.Sc2ySwM_228.2472.1_230-251
AZOFIhc_s0006.g009588_212-235
GINBI_Gb_03048_299-322
PORPU_evm.model.contig_527.5_966-988
CHLAT_Chrsp108S01363_72-94
MARPO_Mapoly0016s0064.1.p_112-134
GONAV_comp58104_c0_seq1_6_ORF9_150_53-75
GUITH_91074_93-115
KLEFL_kfl00161_0140_v1.1_99-121
SPIMU_SM005208S17863_18-40
SPIMU_SM000211S06647_18-40
SPIMU_SM000235S08107_63-85
SPIMU_SM000035S13098_68-90
PHYPAV33_Pp3c20_14750V3.1_40-62
PHYPAV33_Pp3c23_14910V3.1_108-130
ANTAG_AagrBONN_evm.model.Sc2ySwM_228.2626.1_109-131
CHLAT_Chrsp369S08999_106-128
AZOFIhc_s0078.g038282_79-101
GUITH_92575_172-195
GUITH_119054_189-208
GUITH_139300_260-279
ANTAG_AagrBONN_evm.model.Sc2ySwM_362.1676.1_1141-1165
GRACH_PXF49107.1_202-223
CHOCR_CDF40611.1_197-218
PORUM_Pum0120s0020.1.p_252-271
PYRYE_py08319.t1_262-281
PENMA_pm002302.t1_268-289
CHRZO_UNPLg00240.t1_62-84
PORPU_evm.model.contig_3601.2_367-389
ANTAG_AagrBONN_evm.model.Sc2ySwM_228.1690.1_143-162
PENMA_pm006369g0010_182-198
PENMA_pm003446g0010_182-198
PENMA_pm000962g0030_234-250
PENMA_pm002715g0100_169-191
PENMA_pm011342g0030_64-86
ANTAG_AagrBONN_evm.model.Sc2ySwM_117.4094.1_10-28
KLEFL_kfl00063_0300_v1.1_442-465
KLEFL_kfl00644_g2_v1.1_8-28
KLEFL_kfl00644_g2_v1.1_120-136
KLEFL_kfl00644_g2_v1.1_171-190
KLEFL_kfl00063_0300_v1.1_267-285
KLEFL_kfl00644_g2_v1.1_62-82
CALTU_IDg15765t1_187-206
GUITH_137341_225-248
PENMA_pm006369g0010_73-94
PENMA_pm003446g0010_73-94
PENMA_pm000962g0030_125-146
PENMA_pm002302.t1_158-181
PENMA_pm000613g0040_158-181
PENMA_pm012904g0010_119-142
KLEFL_kfl00368_0180_v1.1_70-92
VOLCA_Vocar.0019s0075.1.p_126-149
CHLRE_Cre03.g152150.t1.2_112-135
CHABR_g29834.t1_330-353
ARATH_AT5G03740.1_269-290
ORYSAJA_LOC_Os05g51830.1_272-294
ORYSAJA_LOC_Os01g68160.1_293-316
ARATH_AT3G44750.1_219-241
KLEFL_kfl00046_0090_v1.1_251-270
CHLVU_maker_Scaffold_136-snap-gene-0.53-mRNA-1_329-349
KLEFL_kfl00063_0300_v1.1_154-176
GRACH_PXF40983.1_195-218
GRACH_PXF43775.1_195-218
GRACH_PXF42343.1_378-401
GRACH_PXF41579.1_373-396
ANTAG_AagrBONN_evm.model.Sc2ySwM_362.1676.1_1008-1026
PYRYE_py00396.t1_64-88
ANTAG_AagrBONN_evm.model.Sc2ySwM_344.1162.1_238-258
PHYPAV33_Pp3c27_4140V3.1_223-243
GUITH_102835_45-67
GONAV_g29161.t1_77-99
VOLCA_Vocar.0013s0231.1.p_389-407
CHABR_g22941.t1_866-886
GINBI_Gb_16426_215-239
CHABR_g46910.t1_454-478
SPIMU_SM000074S21666_275-299
SPIMU_SM000149S01347_244-268
SPIMU_SM000435S15745_245-269
AZOFIhc_s0061.g035008_228-252
MARPO_Mapoly0013s0172.1.p_189-213
PHYPAV33_Pp3c4_13250V3.1_210-234
ANTAG_AagrBONN_evm.model.Sc2ySwM_344.2110.1_517-541
PHYPAV33_Pp3c9_15270V3.1_206-230
PHYPAV33_Pp3c18_21380V3.1_261-285
PHYPAV33_Pp3c7_24100V3.1_237-261
PHYPAV33_Pp3c11_2350V3.1_248-272
PHYPAV33_Pp3c21_1210V3.1_267-291
PHYPAV33_Pp3c19_5900V3.1_262-286
CHLAT_Chrsp58S09137_106-130
CHLAT_Chrsp29S04621_133-157
AZOFIhc_s0096.g043711_242-266
AZOFIhc_s0159.g053974_221-245
PENMA_pm003136g0070_372-393
GRACH_PXF47849.1_338-356
AZOFIhc_s0292.g063363_532-553
GRACH_PXF47850.1_278-298
GRACH_PXF47846.1_246-267
GALSU_XP_005706710.1_56-80
CALTU_IDg1910t1_216-239
CALTU_IDg997t1_149-172
KLEFL_kfl00046_0085_v1.1_250-272
ARATH_AT5G63280.1_106-128
GONAV_comp45935_c0_seq1_6_ORF20_137_14-25
GONAV_comp54411_c1_seq1_6_ORF15_280_18-42
GUITH_63305_2-23
VOLCA_Vocar.0020s0149.1.p_15-36
CHLRE_Cre06.g305200.t1.1_15-36
CHRZO_Cz08g24200.t1_14-35
SPIMU_SM003242S12604_13-34
SPIMU_SM003274S12426_13-34
KLEFL_kfl00018_0520_v1.1_14-35
GRACH_PXF47475.1_18-39
CALTU_IDg8947t1_8-25
CALTU_IDg2762t1_145-165
CALTU_IDg22686t1_7-25
PORPU_evm.model.contig_578.2_58-79
KLEFL_kfl00029_0340_v1.1_107-125
KLEFL_kfl00063_0300_v1.1_238-258
PORPU_evm.model.contig_798.2_546-569
GALSU_XP_005709239.1_77-100
CHLAT_Chrsp103S08591_109-133
PORPU_evm.model.contig_2032.25_91-115
PORUM_Pum0120s0020.1.p_119-138
PYRYE_py08319.t1_127-149
CALTU_IDg18001t1_141-162
CHOCR_CDF40611.1_67-86
GRACH_PXF49107.1_72-95
GRACH_PXF44133.1_59-80
CYAME_jgi_CyamerSOOS_1_1114_CmerSOOS_G1271.1_163-185
CYAME_jgi_CyamerSoos_1_1646_CmerSOOS_G1676.1_163-185
GRACH_PXF44605.1_272-295
GRACH_PXF40331.1_383-406
CHRZO_Cz05g11040.t1_7-29
GONAV_comp54411_c1_seq1_6_ORF15_280_43-66
GUITH_63305_24-42
AZOFIhc_s0157.g053789_36-59
MARPO_Mapoly0070s0095.1.p_35-58
PENMA_pm007000g0040_35-58
PENMA_pm006079g0060_35-58
CHABR_g1074.t1_35-58

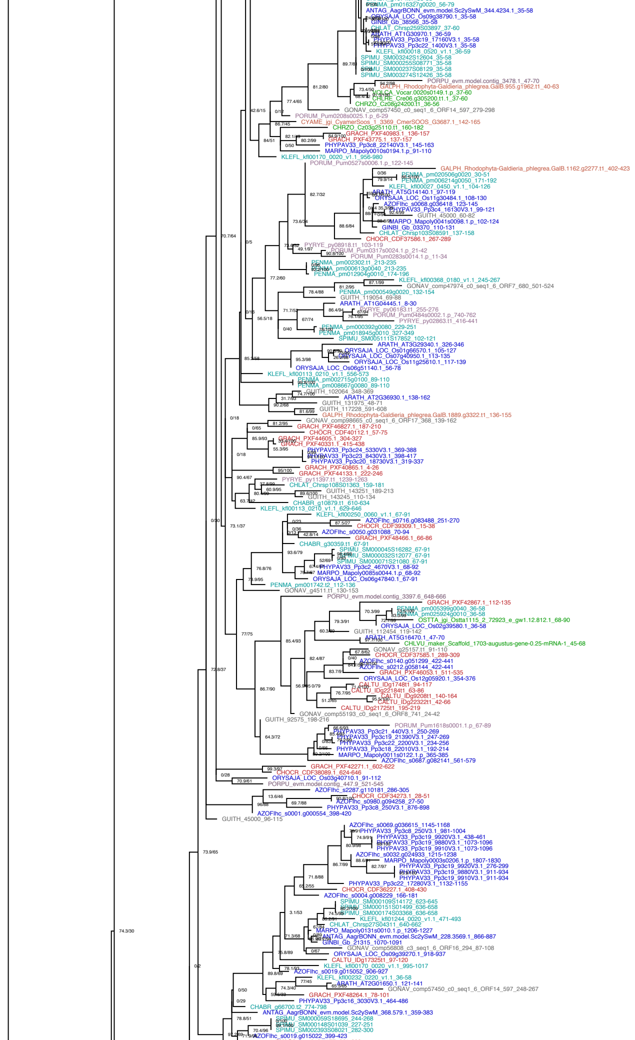
PENMA_pm016327g0020_56-79
ANTAG_AagrBONN_evm.model.Sc2ySwM_344.4234.1_35-58
ORYSAJA_LOC_Os09g38790.1_35-58
GINBI_Gb_38566_35-58
CHLAT_Chrsp259S03897_37-60
ARATH_AT1G30970.1_36-59
PHYPAV33_Pp3c19_17160V3.1_35-58
PHYPAV33_Pp3c22_1400V3.1_35-58
KLEFL_kfl00018_0520_v1.1_36-59
SPIMU_SM003242S12604_35-58
SPIMU_SM000255S08771_35-58
SPIMU_SM000237S08129_35-58
SPIMU_SM003274S12426_35-58
PORPU_evm.model.contig_3478.1_47-70
GALPH_Rhodophyta-Galdieria_phlegrea.GalB.955.g1962.t1_40-63
VOLCA_Vocar.0020s0149.1.p_37-60
CHLRE_Cre06.g305200.t1.1_37-60
CHRZO_Cz08g24200.t1_36-56
GONAV_comp57450_c0_seq1_6_ORF14_597_279-298
PORUM_Pum0208s0025.1.p_6-29
CYAME_jgi_CyamerSoos_1_3369_CmerSOOS_G3687.1_142-165
CHRZO_Cz03g25110.t1_160-182
GRACH_PXF40983.1_136-157
GRACH_PXF43775.1_137-157
PHYPAV33_Pp3c8_22140V3.1_145-163
MARPO_Mapoly0010s0194.1.p_91-110
KLEFL_kfl00170_0020_v1.1_956-980
PORUM_Pum0527s0006.1.p_122-145
GALPH_Rhodophyta-Galdieria_phlegrea.GalB.1162.g2277.t1_402-423
PENMA_pm020506g0020_30-51
PENMA_pm006214g0050_171-192
KLEFL_kfl00027_0450_v1.1_104-126
ARATH_AT5G14140.1_97-119
ORYSAJA_LOC_Os11g30484.1_108-130
AZOFIhc_s0068.g036418_123-145
PHYPAV33_Pp3c4_16130V3.1_99-121
GUITH_45000_60-82
MARPO_Mapoly0041s0098.1.p_102-124
GINBI_Gb_03370_110-131
CHLAT_Chrsp103S08591_137-158
CHOCR_CDF37586.1_267-289
PYRYE_py08918.t1_103-119
PORUM_Pum0317s0024.1.p_21-42
PORUM_Pum0283s0014.1.p_11-34
PENMA_pm002302.t1_213-235
PENMA_pm000613g0040_213-235
PENMA_pm012904g0010_174-196
KLEFL_kfl00368_0180_v1.1_245-267
GONAV_comp47974_c0_seq1_6_ORF7_680_501-524
PENMA_pm000549g0020_132-154
GUITH_119054_69-88
ARATH_AT1G04445.1_8-30
PYRYE_py06183.t1_255-276
PORUM_Pum0484s0002.1.p_740-762
PYRYE_py02863.t1_416-441
PENMA_pm000392g0080_229-251
PENMA_pm018945g0010_327-349
SPIMU_SM005111S17852_102-121
ARATH_AT3G29340.1_326-346
ORYSAJA_LOC_Os01g66570.1_105-127
ORYSAJA_LOC_Os07g40950.1_113-135
ORYSAJA_LOC_Os11g25610.1_117-139
ORYSAJA_LOC_Os06g51140.1_56-78
KLEFL_kfl00113_0210_v1.1_556-573
PENMA_pm002715g0100_89-110
PENMA_pm008667g0080_89-110
GUITH_102064_348-369
ARATH_AT2G36930.1_138-162
GUITH_131975_48-71
GUITH_117228_591-608
GALPH_Rhodophyta-Galdieria_phlegrea.GalB.1889.g3322.t1_136-155
GONAV_comp98665_c0_seq1_6_ORF17_368_139-162
GRACH_PXF46827.1_187-210
CHOCR_CDF40112.1_57-75
GRACH_PXF44605.1_304-327
GRACH_PXF40331.1_415-438
PHYPAV33_Pp3c24_5330V3.1_369-388
PHYPAV33_Pp3c23_8430V3.1_398-417
PHYPAV33_Pp3c20_18730V3.1_319-337
GRACH_PXF40865.1_4-26
GRACH_PXF44133.1_222-246
PYRYE_py11397.t1_1239-1263
CHLAT_Chrsp108S01363_159-181
GUITH_143251_189-213
GUITH_143245_110-134
CHABR_g10879.t1_610-634
KLEFL_kfl00113_0210_v1.1_629-646
KLEFL_kfl00250_0060_v1.1_67-91
AZOFIhc_s0716.g083488_251-270
CHOCR_CDF39309.1_15-38
AZOFIhc_s0050.g031088_70-94
GRACH_PXF48466.1_66-86
CHABR_g30359.t1_67-91
SPIMU_SM000045S16282_67-91
SPIMU_SM000032S12077_67-91
SPIMU_SM000071S21080_67-91
PHYPAV33_Pp3c2_4670V3.1_68-92
MARPO_Mapoly0085s0044.1.p_68-92
ORYSAJA_LOC_Os06g47840.1_67-91
PENMA_pm001742.t2_112-136
GONAV_g4511.t1_130-153
PORPU_evm.model.contig_3397.6_648-666
GRACH_PXF42867.1_112-135
PENMA_pm005399g0040_36-58
PENMA_pm025924g0010_36-58
OSTTA_jgi_Ostta1115_2_72923_e_gw1.12.812.1_68-90
ORYSAJA_LOC_Os02g39580.1_36-58
GUITH_112454_119-142
ARATH_AT5G16470.1_47-70
CHLVU_maker_Scaffold_1703-augustus-gene-0.25-mRNA-1_45-68
GONAV_g25157.t1_91-110
CHOCR_CDF37585.1_289-309
AZOFIhc_s0140.g051299_422-441
AZOFIhc_s0212.g058144_422-441
GRACH_PXF46053.1_511-535
ORYSAJA_LOC_Os12g05920.1_354-376
CALTU_IDg1748t1_94-117
CALTU_IDg22184t1_63-86
CALTU_IDg9208t1_140-164
CALTU_IDg22322t1_42-66
CALTU_IDg21725t1_195-219
GONAV_comp55193_c0_seq1_6_ORF8_741_24-42
GUITH_92575_198-216
PORUM_Pum1618s0001.1.p_67-89
PHYPAV33_Pp3c21_440V3.1_250-269
PHYPAV33_Pp3c19_21390V3.1_247-269
PHYPAV33_Pp3c22_2200V3.1_234-256
PHYPAV33_Pp3c18_22010V3.1_192-214
MARPO_Mapoly0011s0122.1.p_365-385
AZOFIhc_s0687.g082141_561-579
GRACH_PXF42271.1_602-622
CHOCR_CDF38089.1_624-646
ORYSAJA_LOC_Os03g40710.1_91-112
PORPU_evm.model.contig_447.9_521-545
AZOFIhc_s2287.g110181_286-305
CHOCR_CDF34273.1_28-51
AZOFIhc_s0980.g094258_27-50
PHYPAV33_Pp3c8_250V3.1_876-898
AZOFIhc_s0001.g000554_398-420
GUITH_45000_96-115
AZOFIhc_s0069.g036615_1145-1168
PHYPAV33_Pp3c8_250V3.1_981-1004
PHYPAV33_Pp3c19_9920V3.1_438-461
PHYPAV33_Pp3c19_9880V3.1_1073-1096
PHYPAV33_Pp3c19_9910V3.1_1073-1096
AZOFIhc_s0032.g024933_1215-1238
MARPO_Mapoly0003s0206.1.p_1807-1830
PHYPAV33_Pp3c19_9920V3.1_276-299
PHYPAV33_Pp3c19_9880V3.1_911-934
PHYPAV33_Pp3c19_9910V3.1_911-934
PHYPAV33_Pp3c22_17280V3.1_1132-1155
CHOCR_CDF36227.1_408-430
AZOFIhc_s0004.g008229_166-181
SPIMU_SM000109S14172_623-645
SPIMU_SM000151S01499_636-658
SPIMU_SM000174S03368_636-658
KLEFL_kfl01244_0020_v1.1_471-493
CHLAT_Chrsp27S04311_640-662
MARPO_Mapoly0131s0010.1.p_1206-1227
ANTAG_AagrBONN_evm.model.Sc2ySwM_228.3569.1_866-887
GINBI_Gb_21315_1070-1091
GONAV_comp56808_c3_seq1_6_ORF16_294_87-108
ORYSAJA_LOC_Os09g39270.1_918-937
CALTU_IDg17325t1_97-120
KLEFL_kfl00170_0020_v1.1_995-1017
AZOFIhc_s0019.g015052_906-927
KLEFL_kfl00232_0220_v1.1_36-58
ARATH_AT2G01650.1_121-141
GONAV_comp57450_c0_seq1_6_ORF14_597_248-267
GRACH_PXF48264.1_78-101
PHYPAV33_Pp3c16_3030V3.1_464-486
CHABR_g66700.t2_774-798
ANTAG_AagrBONN_evm.model.Sc2ySwM_368.579.1_359-383
SPIMU_SM000059S18695_244-268
SPIMU_SM000148S01039_227-251
SPIMU_SM002393S08021_282-300
AZOFIhc_s0019.g015022_399-423

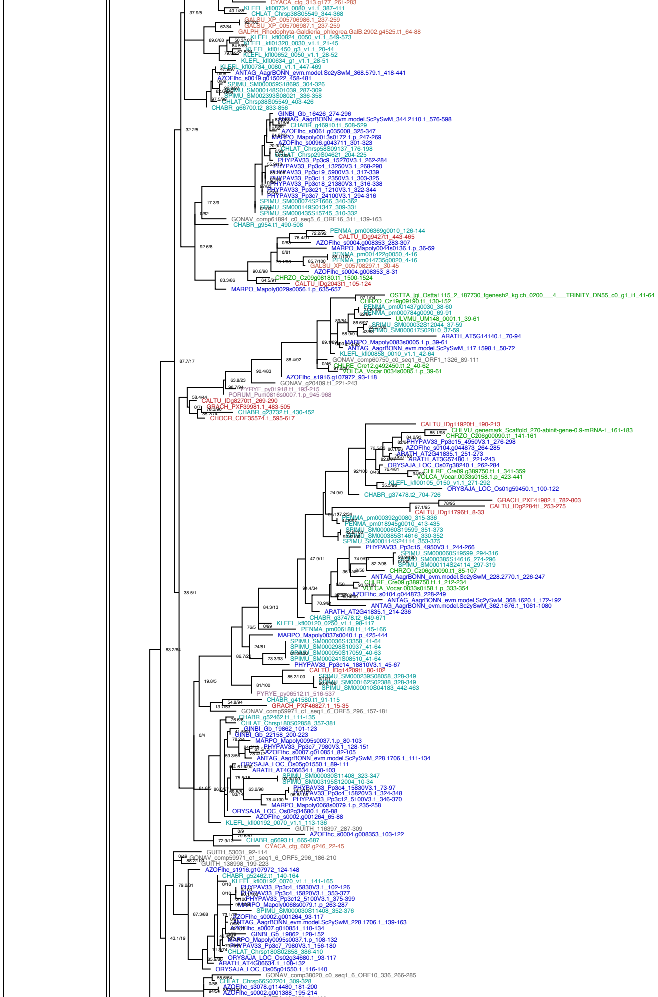

CYACA_ctg_313.g177_261-283
KLEFL_kfl00734_0080_v1.1_387-411
CHLAT_Chrsp38S05549_344-368
GALSU_XP_005706986.1_237-259
GALSU_XP_005706987.1_237-259
GALPH_Rhodophyta-Galdieria_phlegrea.GalB.2902.g4525.t1_64-88
KLEFL_kfl00824_0050_v1.1_549-573
KLEFL_kfl01320_0030_v1.1_21-45
KLEFL_kfl01450_g3_v1.1_20-44
KLEFL_kfl00652_0050_v1.1_28-52
KLEFL_kfl00634_g1_v1.1_28-51
KLEFL_kfl00734_0080_v1.1_447-469
ANTAG_AagrBONN_evm.model.Sc2ySwM_368.579.1_418-441
AZOFIhc_s0019.g015022_458-481
SPIMU_SM000059S18695_304-326
SPIMU_SM000148S01039_287-309
SPIMU_SM002393S08021_336-358
CHLAT_Chrsp38S05549_403-426
CHABR_g66700.t2_833-856
GINBI_Gb_16426_274-296
ANTAG_AagrBONN_evm.model.Sc2ySwM_344.2110.1_576-598
CHABR_g46910.t1_508-529
AZOFIhc_s0061.g035008_325-347
MARPO_Mapoly0013s0172.1.p_247-269
AZOFIhc_s0096.g043711_301-323
CHLAT_Chrsp58S09137_176-198
CHLAT_Chrsp29S04621_204-225
PHYPAV33_Pp3c9_15270V3.1_262-284
PHYPAV33_Pp3c4_13250V3.1_268-290
PHYPAV33_Pp3c19_5900V3.1_317-339
PHYPAV33_Pp3c11_2350V3.1_303-325
PHYPAV33_Pp3c18_21380V3.1_316-338
PHYPAV33_Pp3c21_1210V3.1_322-344
PHYPAV33_Pp3c7_24100V3.1_294-316
SPIMU_SM000074S21666_340-362
SPIMU_SM000149S01347_309-331
SPIMU_SM000435S15745_310-332
GONAV_comp61894_c0_seq5_6_ORF16_311_139-163
CHABR_g954.t1_490-508
PENMA_pm006369g0010_126-144
CALTU_IDg9427t1_443-465
AZOFIhc_s0004.g008353_283-307
MARPO_Mapoly0044s0136.1.p_36-59
PENMA_pm001422g0050_4-16
PENMA_pm014735g0020_4-16
GALSU_XP_005708297.1_30-45
AZOFIhc_s0004.g008353_8-31
CHRZO_Cz09g08180.t1_1500-1524
CALTU_IDg2043t1_105-124
MARPO_Mapoly0029s0056.1.p_635-657
OSTTA_jgi_Ostta1115_2_187730_fgenesh2_kg.ch_0200___4___TRINITY_DN55_c0_g1_i1_41-64
CHRZO_Cz19g09190.t1_130-152
PENMA_pm001437g0030_38-60
PENMA_pm000784g0090_69-91
ULVMU_UM148_0001.1_39-61
SPIMU_SM000032S12044_37-59
SPIMU_SM000017S02810_37-59
ARATH_AT5G14140.1_70-94
MARPO_Mapoly0083s0005.1.p_39-61
ANTAG_AagrBONN_evm.model.Sc2ySwM_117.1598.1_50-72
KLEFL_kfl00858_0010_v1.1_42-64
GONAV_comp60750_c0_seq1_6_ORF1_1326_89-111
CHLRE_Cre12.g492450.t1.2_40-62
VOLCA_Vocar.0034s0085.1.p_39-61
AZOFIhc_s1916.g107972_93-118
GONAV_g20409.t1_221-243
PYRYE_py01918.t1_193-215
PORUM_Pum0816s0007.1.p_945-968
CALTU_IDg8270t1_269-290
GRACH_PXF39981.1_483-505
CHABR_g23732.t1_430-452
CHOCR_CDF35574.1_595-617
CALTU_IDg11920t1_190-213
CHLVU_genemark_Scaffold_270-abinit-gene-0.9-mRNA-1_161-183
CHRZO_Cz06g00090.t1_141-161
PHYPAV33_Pp3c15_4950V3.1_276-298
AZOFIhc_s0104.g044873_264-285
ARATH_AT2G41835.1_251-273
ARATH_AT3G57480.1_221-243
ORYSAJA_LOC_Os07g38240.1_262-284
CHLRE_Cre09.g389750.t1.1_341-359
VOLCA_Vocar.0033s0158.1.p_423-441
KLEFL_kfl00105_0150_v1.1_271-292
ORYSAJA_LOC_Os01g59450.1_100-122
CHABR_g37478.t2_704-726
GRACH_PXF41982.1_782-803
CALTU_IDg2284t1_253-275
CALTU_IDg11796t1_8-33
PENMA_pm000392g0080_315-336
PENMA_pm018945g0010_413-435
SPIMU_SM000060S19599_351-373
SPIMU_SM000385S14616_330-352
SPIMU_SM000114S24114_353-375
PHYPAV33_Pp3c15_4950V3.1_244-266
SPIMU_SM000060S19599_294-316
SPIMU_SM000385S14616_274-296
SPIMU_SM000114S24114_297-319
CHRZO_Cz06g00090.t1_85-107
ANTAG_AagrBONN_evm.model.Sc2ySwM_228.2770.1_226-247
CHLRE_Cre09.g389750.t1.1_212-234
VOLCA_Vocar.0033s0158.1.p_333-354
AZOFIhc_s0104.g044873_228-249
ANTAG_AagrBONN_evm.model.Sc2ySwM_368.1620.1_172-192
ANTAG_AagrBONN_evm.model.Sc2ySwM_362.1676.1_1061-1080
ARATH_AT2G41835.1_214-236
CHABR_g37478.t2_649-671
KLEFL_kfl00120_0250_v1.1_98-117
PENMA_pm006188.t1_145-166
MARPO_Mapoly0037s0040.1.p_425-444
SPIMU_SM000036S13358_41-64
SPIMU_SM000298S10937_41-64
SPIMU_SM000050S17059_40-63
SPIMU_SM000241S08510_41-64
PHYPAV33_Pp3c14_18810V3.1_45-67
CALTU_IDg14209t1_80-102
SPIMU_SM000239S08058_328-349
SPIMU_SM000162S02388_328-349
SPIMU_SM000010S04183_442-463
PYRYE_py06512.t1_516-537
CHABR_g41580.t1_91-115
GRACH_PXF46827.1_15-35
GONAV_comp59971_c1_seq1_6_ORF5_296_157-181
CHABR_g52462.t1_111-135
CHLAT_Chrsp180S02858_357-381
GINBI_Gb_19862_101-123
GINBI_Gb_22158_200-223
MARPO_Mapoly0095s0037.1.p_80-103
PHYPAV33_Pp3c7_7980V3.1_128-151
AZOFIhc_s0007.g010851_82-105
ANTAG_AagrBONN_evm.model.Sc2ySwM_228.1706.1_111-134
ORYSAJA_LOC_Os05g01550.1_89-111
ARATH_AT4G06634.1_80-103
SPIMU_SM000030S11408_323-347
SPIMU_SM003195S12004_10-34
PHYPAV33_Pp3c4_15830V3.1_73-97
PHYPAV33_Pp3c4_15820V3.1_324-348
PHYPAV33_Pp3c12_5100V3.1_346-370
MARPO_Mapoly0068s0079.1.p_235-258
ORYSAJA_LOC_Os02g34680.1_66-88
AZOFIhc_s0002.g001264_65-88
KLEFL_kfl00192_0070_v1.1_113-136
GUITH_116397_287-309
AZOFIhc_s0004.g008353_103-122
CHABR_g6693.t1_665-687
CYACA_ctg_602.g246_22-45
GUITH_53031_92-114
GONAV_comp59971_c1_seq1_6_ORF5_296_186-210
GUITH_138998_199-223
AZOFIhc_s1916.g107972_124-148
CHABR_g52462.t1_140-164
KLEFL_kfl00192_0070_v1.1_141-165
PHYPAV33_Pp3c4_15830V3.1_102-126
PHYPAV33_Pp3c4_15820V3.1_353-377
PHYPAV33_Pp3c12_5100V3.1_375-399
MARPO_Mapoly0068s0079.1.p_263-287
SPIMU_SM000030S11408_352-376
AZOFIhc_s0002.g001264_93-117
ANTAG_AagrBONN_evm.model.Sc2ySwM_228.1706.1_139-163
AZOFIhc_s0007.g010851_110-134
GINBI_Gb_19862_128-152
MARPO_Mapoly0095s0037.1.p_108-132
PHYPAV33_Pp3c7_7980V3.1_156-180
CHLAT_Chrsp180S02858_386-410
ORYSAJA_LOC_Os02g34680.1_93-117
ARATH_AT4G06634.1_108-132
ORYSAJA_LOC_Os05g01550.1_116-140
GONAV_comp38020_c0_seq1_6_ORF10_336_266-285
CHLAT_Chrsp66S07201_309-328
AZOFIhc_s3078.g114480_181-200
AZOFIhc_s0002.g001388_195-214

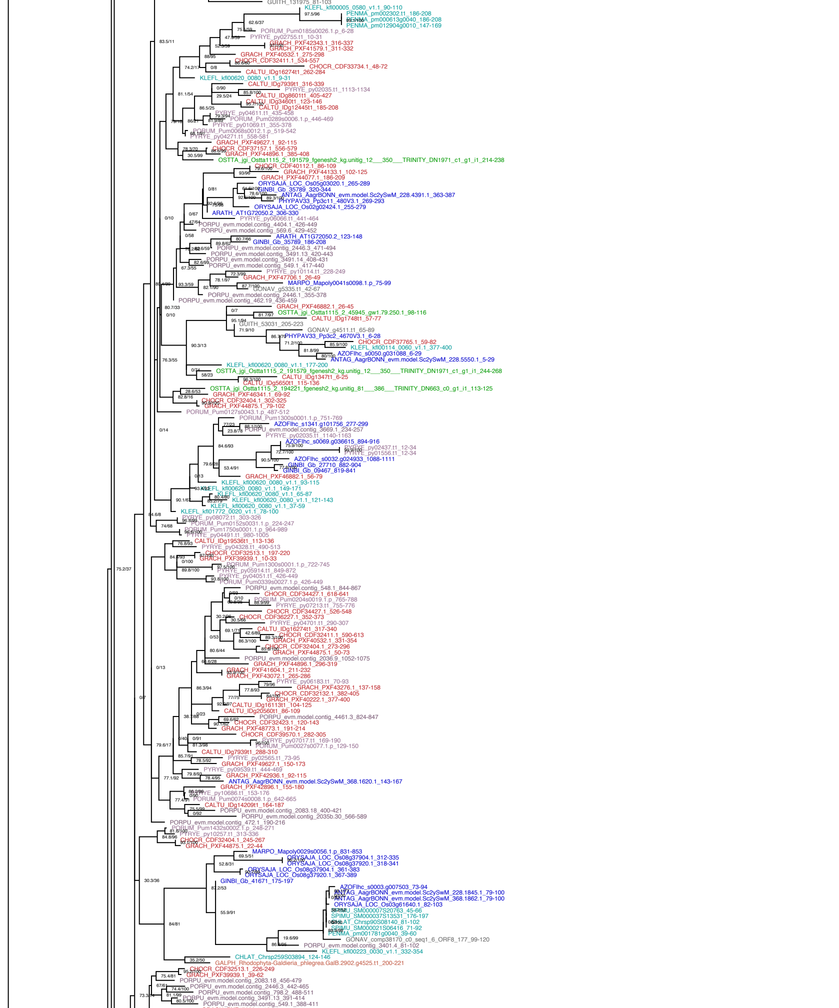
GUITH_131975_81-103
KLEFL_kfl00005_0580_v1.1_90-110
PENMA_pm002302.t1_186-208
PENMA_pm000613g0040_186-208
PENMA_pm012904g0010_147-169
PORUM_Pum0185s0026.1.p_6-28
PYRYE_py02755.t1_10-31
GRACH_PXF42343.1_316-337
GRACH_PXF41579.1_311-332
GRACH_PXF40532.1_275-298
CHOCR_CDF32411.1_534-557
CHOCR_CDF33734.1_48-72
CALTU_IDg16274t1_262-284
KLEFL_kfl00620_0080_v1.1_9-31
CALTU_IDg7939t1_316-339
PYRYE_py02035.t1_1113-1134
CALTU_IDg8601t1_405-427
CALTU_IDg3460t1_123-146
CALTU_IDg12445t1_185-208
PYRYE_py04611.t1_435-458
PORUM_Pum0289s0006.1.p_446-469
PYRYE_py01069.t1_355-378
PORUM_Pum0068s0012.1.p_519-542
PYRYE_py04271.t1_558-581
GRACH_PXF49627.1_92-115
CHOCR_CDF37157.1_556-579
GRACH_PXF44896.1_385-408
OSTTA_jgi_Ostta1115_2_191579_fgenesh2_kg.unitig_12___350___TRINITY_DN1971_c1_g1_i1_214-238
CHOCR_CDF40112.1_86-109
GRACH_PXF44133.1_102-125
GRACH_PXF44077.1_186-209
ORYSAJA_LOC_Os05g03020.1_265-289
GINBI_Gb_35789_320-344
ANTAG_AagrBONN_evm.model.Sc2ySwM_228.4391.1_363-387
PHYPAV33_Pp3c11_480V3.1_269-293
ORYSAJA_LOC_Os02g02424.1_255-279
ARATH_AT1G72050.2_306-330
PYRYE_py06066.t1_441-464
PORPU_evm.model.contig_4404.1_426-449
PORPU_evm.model.contig_569.6_429-452
ARATH_AT1G72050.2_123-148
GINBI_Gb_35789_186-208
PORPU_evm.model.contig_2446.3_471-494
PORPU_evm.model.contig_3491.13_420-443
PORPU_evm.model.contig_3491.14_408-431
PORPU_evm.model.contig_549.1_417-440
PYRYE_py10114.t1_228-249
GRACH_PXF47706.1_26-49
MARPO_Mapoly0041s0098.1.p_75-99
GONAV_g5335.t1_42-67
PORPU_evm.model.contig_2446.1_355-378
PORPU_evm.model.contig_462.19_436-459
GRACH_PXF46882.1_26-45
OSTTA_jgi_Ostta1115_2_45945_gw1.79.250.1_98-116
CALTU_IDg1748t1_57-77
GUITH_53031_205-223
GONAV_g4511.t1_65-89
PHYPAV33_Pp3c2_4670V3.1_6-28
CHOCR_CDF37765.1_59-82
KLEFL_kfl00114_0060_v1.1_377-400
AZOFIhc_s0050.g031088_6-29
ANTAG_AagrBONN_evm.model.Sc2ySwM_228.5550.1_5-29
KLEFL_kfl00620_0080_v1.1_177-200
OSTTA_jgi_Ostta1115_2_191579_fgenesh2_kg.unitig_12___350___TRINITY_DN1971_c1_g1_i1_244-268
CALTU_IDg1347t1_6-25
CALTU_IDg5650t1_115-136
OSTTA_jgi_Ostta1115_2_194221_fgenesh2_kg.unitig_81___386___TRINITY_DN663_c0_g1_i1_113-125
GRACH_PXF46341.1_69-92
CHOCR_CDF32404.1_302-325
GRACH_PXF44875.1_79-102
PORUM_Pum0127s0043.1.p_487-512
PORUM_Pum1300s0001.1.p_751-769
AZOFIhc_s1341.g101756_277-299
PORPU_evm.model.contig_3669.1_234-257
PYRYE_py02035.t1_1140-1163
AZOFIhc_s0069.g036615_894-916
PYRYE_py02437.t1_12-34
PYRYE_py01556.t1_12-34
AZOFIhc_s0032.g024933_1088-1111
GINBI_Gb_27710_882-904
GINBI_Gb_09467_819-841
GRACH_PXF46882.1_56-79
KLEFL_kfl00620_0080_v1.1_93-115
KLEFL_kfl00620_0080_v1.1_149-171
KLEFL_kfl00620_0080_v1.1_65-87
KLEFL_kfl00620_0080_v1.1_121-143
KLEFL_kfl00620_0080_v1.1_37-59
KLEFL_kfl01772_0020_v1.1_78-100
PYRYE_py08072.t1_303-326
PORUM_Pum0152s0031.1.p_224-247
PORUM_Pum1750s0001.1.p_964-989
PYRYE_py04491.t1_980-1005
CALTU_IDg19536t1_113-136
PYRYE_py04328.t1_490-513
CHOCR_CDF32513.1_197-220
GRACH_PXF39939.1_10-33
PORUM_Pum1300s0001.1.p_722-745
PYRYE_py05914.t1_849-872
PYRYE_py04051.t1_426-449
PORUM_Pum0339s0027.1.p_426-449
PORPU_evm.model.contig_548.1_844-867
CHOCR_CDF34427.1_618-641
PORUM_Pum0204s0019.1.p_765-788
PYRYE_py07213.t1_755-776
CHOCR_CDF34427.1_526-548
CHOCR_CDF36227.1_352-373
PYRYE_py04701.t1_290-307
CALTU_IDg16274t1_317-340
CHOCR_CDF32411.1_590-613
GRACH_PXF40532.1_331-354
CHOCR_CDF32404.1_273-296
GRACH_PXF44875.1_50-73
PORPU_evm.model.contig_2036.9_1052-1075
GRACH_PXF44896.1_296-319
GRACH_PXF41604.1_211-232
GRACH_PXF43072.1_265-286
PYRYE_py06183.t1_70-93
GRACH_PXF43276.1_137-158
CHOCR_CDF32132.1_382-405
GRACH_PXF40222.1_377-400
CALTU_IDg16113t1_104-125
CALTU_IDg20560t1_86-109
PORPU_evm.model.contig_4461.3_824-847
CHOCR_CDF32423.1_120-143
GRACH_PXF48773.1_191-214
CHOCR_CDF39570.1_282-305
PYRYE_py07017.t1_169-190
PORUM_Pum0027s0077.1.p_129-150
CALTU_IDg7939t1_288-310
PYRYE_py02565.t1_73-95
GRACH_PXF49627.1_150-173
PYRYE_py09539.t1_444-469
GRACH_PXF42936.1_92-115
ANTAG_AagrBONN_evm.model.Sc2ySwM_368.1620.1_143-167
GRACH_PXF42896.1_155-180
PYRYE_py10686.t1_153-176
PORUM_Pum0074s0008.1.p_642-665
CALTU_IDg14209t1_164-187
PORPU_evm.model.contig_2083.18_400-421
PORPU_evm.model.contig_2035b.30_566-589
PORPU_evm.model.contig_472.1_190-216
PORUM_Pum1432s0002.1.p_248-271
PYRYE_py10257.t1_313-336
CHOCR_CDF32404.1_245-267
GRACH_PXF44875.1_22-44
MARPO_Mapoly0029s0056.1.p_831-853
ORYSAJA_LOC_Os08g37904.1_312-335
ORYSAJA_LOC_Os08g37920.1_318-341
ORYSAJA_LOC_Os08g37904.1_361-383
ORYSAJA_LOC_Os08g37920.1_367-389
GINBI_Gb_41671_175-197
AZOFIhc_s0003.g007503_73-94
ANTAG_AagrBONN_evm.model.Sc2ySwM_228.1845.1_79-100
ANTAG_AagrBONN_evm.model.Sc2ySwM_368.1862.1_79-100
ORYSAJA_LOC_Os03g61640.1_82-103
SPIMU_SM000007S20763_45-66
SPIMU_SM000037S13531_176-197
CHLAT_Chrsp90S08140_81-102
SPIMU_SM000021S06416_71-92
PENMA_pm001781g0040_39-60
GONAV_comp38170_c0_seq1_6_ORF8_177_99-120
PORPU_evm.model.contig_3401.4_81-102
KLEFL_kfl00223_0030_v1.1_332-354
CHLAT_Chrsp259S03894_124-146
GALPH_Rhodophyta-Galdieria_phlegrea.GalB.2902.g4525.t1_200-221
CHOCR_CDF32513.1_226-249
GRACH_PXF39939.1_39-62
PORPU_evm.model.contig_2083.18_456-479
PORPU_evm.model.contig_2446.3_442-465
PORPU_evm.model.contig_798.2_488-511
PORPU_evm.model.contig_3491.13_391-414
PORPU_evm.model.contig_549.1_388-411

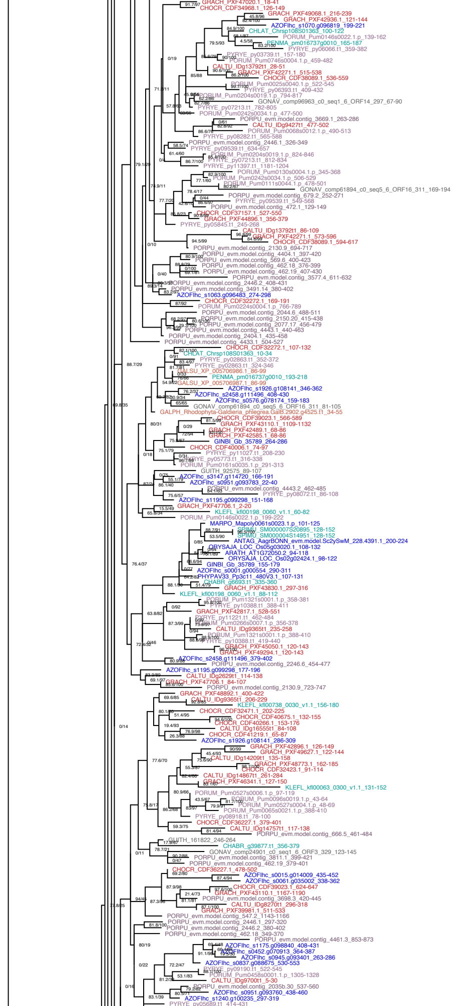
GRACH_PXF47020.1_18-41
CHOCR_CDF34968.1_126-149
GRACH_PXF49068.1_216-239
GRACH_PXF42936.1_121-144
AZOFlhc_s1070.g096819_199-221
CHLAT_Chrsp108S01363_100-122
PORUM_Pum0146s0022.1.p_139-162
PENMA_pm016737g0010_165-187
PYRYE_py06066.t1_359-382
PYRYE_py03739.t1_157-180
PORUM_Pum0746s0004.1.p_459-482
CALTU_IDg13792t1_28-51
GRACH_PXF42271.1_515-538
CHOCR_CDF38089.1_536-559
PORUM_Pum0025s0040.1.p_522-545
PYRYE_py06393.t1_409-432
PORUM_Pum0204s0019.1.p_794-817
GONAV_comp96963_c0_seq1_6_ORF14_297_67-90
PYRYE_py07213.t1_782-805
PORUM_Pum0242s0034.1.p_477-500
PORPU_evm.model.contig_3669.1_263-286
CALTU_IDg9427t1_477-502
PORUM_Pum0068s0012.1.p_490-513
PYRYE_py08282.t1_565-588
PORPU_evm.model.contig_2446.1_326-349
PYRYE_py09539.t1_634-657
PORUM_Pum0204s0019.1.p_824-846
PYRYE_py07213.t1_812-834
PYRYE_py11397.t1_1181-1204
PORUM_Pum0130s0004.1.p_345-368
PORUM_Pum0242s0034.1.p_506-529
PORUM_Pum0111s0044.1.p_478-501
GONAV_comp61894_c0_seq5_6_ORF16_311_169-194
PORPU_evm.model.contig_679.2_252-271
PYRYE_py09539.t1_549-568
PORPU_evm.model.contig_472.1_129-149
CHOCR_CDF37157.1_527-550
GRACH_PXF44896.1_356-379
PYRYE_py05845.t1_245-268
CALTU_IDg13792t1_86-109
GRACH_PXF42271.1_573-596
CHOCR_CDF38089.1_594-617
PORPU_evm.model.contig_2130.9_694-717
PORPU_evm.model.contig_4404.1_397-420
PORPU_evm.model.contig_569.6_400-423
PORPU_evm.model.contig_462.18_376-399
PORPU_evm.model.contig_462.19_407-430
PORPU_evm.model.contig_3577.4_611-632
PORPU_evm.model.contig_2446.2_408-431
PORPU_evm.model.contig_3491.14_380-402
AZOFlhc_s1063.g096483_274-298
CHOCR_CDF32272.1_169-191
PORUM_Pum0224s0004.1.p_766-789
PORPU_evm.model.contig_2044.6_488-511
PORPU_evm.model.contig_2150.20_415-438
PORPU_evm.model.contig_2077.17_456-479
PORPU_evm.model.contig_4443.1_440-463
PORPU_evm.model.contig_2404.1_435-458
PORPU_evm.model.contig_4433.1_504-527
CHOCR_CDF32272.1_107-132
CHLAT_Chrsp108S01363_10-34
PYRYE_py02863.t1_352-372
PYRYE_py02863.t1_324-346
GALSU_XP_005706986.1_86-99
PENMA_pm016737g0010_193-218
GALSU_XP_005706987.1_86-99
AZOFlhc_s1926.g108141_346-362
AZOFlhc_s2458.g111496_408-430
AZOFlhc_s0576.g078174_159-183
GONAV_comp61894_c0_seq5_6_ORF16_311_81-105
GALPH_Rhodophyta-Galdieria_phlegrea.GalB.2902.g4525.t1_34-55
CHOCR_CDF39023.1_566-589
GRACH_PXF43110.1_1109-1132
GRACH_PXF42489.1_68-86
GRACH_PXF42585.1_68-86
GINBI_Gb_35789_264-286
CHOCR_CDF40006.1_74-97
PYRYE_py11027.t1_208-230
PYRYE_py05773.t1_316-338
PORUM_Pum0161s0035.1.p_291-313
GUITH_92575_89-107
AZOFlhc_s3147.g114720_166-191
AZOFlhc_s0951.g093783_22-40
PORPU_evm.model.contig_4443.2_462-485
PYRYE_py08072.t1_86-108
AZOFlhc_s1195.g099298_151-168
GRACH_PXF47706.1_2-20
KLEFL_kfl00198_0060_v1.1_60-82
PORUM_Pum0146s0022.1.p_199-222
MARPO_Mapoly0061s0023.1.p_101-125
SPIMU_SM000007S20895_128-152
SPIMU_SM000004S14951_128-152
ANTAG_AagrBONN_evm.model.Sc2ySwM_228.4391.1_200-224
ORYSAJA_LOC_Os05g03020.1_108-132
ARATH_AT1G72050.2_94-118
ORYSAJA_LOC_Os02g02424.1_98-122
GINBI_Gb_35789_155-179
AZOFlhc_s0001.g000554_290-311
PHYPAV33_Pp3c11_480V3.1_107-131
CHABR_g6693.t1_335-360
GRACH_PXF43830.1_297-316
KLEFL_kfl00198_0060_v1.1_88-112
PORUM_Pum1321s0001.1.p_358-381
PYRYE_py10388.t1_388-411
GRACH_PXF42817.1_528-551
PYRYE_py11221.t1_462-484
PORUM_Pum0266s0007.1.p_356-378
CALTU_IDg9365t1_235-258
PORUM_Pum1321s0001.1.p_388-410
PYRYE_py10388.t1_419-440
GRACH_PXF45050.1_120-143
GRACH_PXF49294.1_120-143
AZOFlhc_s2458.g111496_379-402
PORPU_evm.model.contig_2246.6_454-477
AZOFlhc_s1195.g099298_177-196
CALTU_IDg2629t1_114-138
GRACH_PXF47706.1_84-107
PORPU_evm.model.contig_2130.9_723-747
GRACH_PXF48892.1_400-422
CALTU_IDg9365t1_206-229
KLEFL_kfl00738_0030_v1.1_156-180
CHOCR_CDF32471.1_202-225
CHOCR_CDF40675.1_132-155
CHOCR_CDF40266.1_153-176
CALTU_IDg16555t1_84-108
CHOCR_CDF41219.1_65-87
AZOFlhc_s1926.g108141_286-309
GRACH_PXF42896.1_126-149
GRACH_PXF49627.1_122-144
CALTU_IDg14209t1_135-158
GRACH_PXF48773.1_162-185
CHOCR_CDF32423.1_91-114
CALTU_IDg14867t1_261-284
GRACH_PXF46341.1_127-150
KLEFL_kfl00063_0300_v1.1_131-152
PORUM_Pum0527s0006.1.p_97-119
PORUM_Pum0096s0019.1.p_43-64
PORUM_Pum0527s0004.1.p_48-69
PORUM_Pum0065s0021.1.p_388-410
PYRYE_py08918.t1_78-100
CHOCR_CDF36227.1_379-401
CALTU_IDg14757t1_117-138
PORPU_evm.model.contig_666.5_461-484
GUITH_161822_246-264
CHABR_g39877.t1_356-379
GONAV_comp24901_c0_seq1_6_ORF3_329_123-145
PORPU_evm.model.contig_3811.1_399-421
PORPU_evm.model.contig_462.19_379-401
CHOCR_CDF36227.1_478-502
AZOFlhc_s0015.g014009_435-452
AZOFlhc_s0061.g035002_338-362
CHOCR_CDF39023.1_624-647
GRACH_PXF43110.1_1167-1190
PORPU_evm.model.contig_3698.3_420-445
CALTU_IDg8270t1_296-318
GRACH_PXF39981.1_511-533
PORPU_evm.model.contig_547.2_1143-1166
PORPU_evm.model.contig_2446.1_297-320
PORPU_evm.model.contig_2446.2_380-402
PORPU_evm.model.contig_462.18_349-370
PORPU_evm.model.contig_4461.3_853-873
AZOFlhc_s1175.g098840_408-431
AZOFlhc_s0452.g070913_364-387
AZOFlhc_s0945.g093401_263-286
AZOFlhc_s0837.g088675_530-553
PYRYE_py09190.t1_522-545
PORUM_Pum0458s0001.1.p_1305-1328
CALTU_IDg9700t1_5-30
PORPU_evm.model.contig_2035b.30_537-560
AZOFlhc_s0951.g093760_438-460
AZOFlhc_s1240.g100235_297-319
PYRYE_py05689.t1_414-431

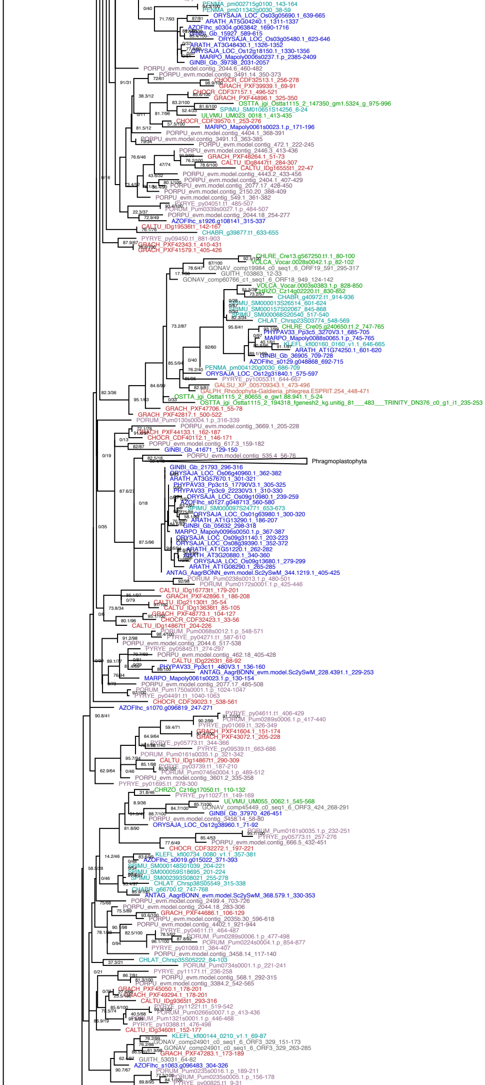

PENMA_pm002715g0100_143-164
PENMA_pm011342g0030_38-59
ORYSAJA_LOC_Os03g05690.1_639-665
ARATH_AT5G04240.1_1311-1337
AZOFIhc_s0304.g063842_1690-1716
GINBI_Gb_15927_589-615
ORYSAJA_LOC_Os03g05480.1_623-646
ARATH_AT3G48430.1_1326-1352
ORYSAJA_LOC_Os12g18150.1_1330-1356
MARPO_Mapoly0006s0237.1.p_2385-2409
GINBI_Gb_39738_2031-2057
PORPU_evm.model.contig_2044.6_460-482
PORPU_evm.model.contig_3491.14_350-373
CHOCR_CDF32513.1_256-278
GRACH_PXF39939.1_69-91
CHOCR_CDF37157.1_496-521
GRACH_PXF44896.1_325-350
OSTTA_jgi_Ostta1115_2_147350_gm1.5324_g_975-996
SPIMU_SM010651S14256_6-24
ULVMU_UM023_0018.1_413-435
CHOCR_CDF39570.1_253-276
MARPO_Mapoly0061s0023.1.p_171-196
PORPU_evm.model.contig_4404.1_368-391
PORPU_evm.model.contig_3491.13_363-385
PORPU_evm.model.contig_472.1_222-245
PORPU_evm.model.contig_2446.3_413-436
GRACH_PXF48264.1_51-73
CALTU_IDg8447t1_284-307
CALTU_IDg16555t1_22-47
PORPU_evm.model.contig_4443.2_433-456
PORPU_evm.model.contig_2404.1_407-429
PORPU_evm.model.contig_2077.17_428-450
PORPU_evm.model.contig_2150.20_388-409
PORPU_evm.model.contig_549.1_361-382
PYRYE_py04051.t1_485-507
PORUM_Pum0339s0027.1.p_484-507
PORPU_evm.model.contig_2044.18_254-277
AZOFIhc_s1926.g108141_315-337
CALTU_IDg19536t1_142-167
CHABR_g39877.t1_633-655
PYRYE_py09450.t1_881-903
GRACH_PXF42343.1_410-431
GRACH_PXF41579.1_405-426
CHLRE_Cre13.g567250.t1.1_80-100
VOLCA_Vocar.0028s0042.1.p_82-102
GONAV_comp19984_c0_seq1_6_ORF19_591_295-317
GUITH_103863_12-33
GONAV_comp60766_c1_seq1_6_ORF18_949_124-142
VOLCA_Vocar.0003s0383.1.p_828-850
CHRZO_Cz14g02220.t1_830-852
CHABR_g40972.t1_914-936
SPIMU_SM000013S26514_601-624
SPIMU_SM000157S02067_845-868
SPIMU_SM000068S20540_517-540
CHLAT_Chrsp23S03774_548-569
CHLRE_Cre05.g240650.t1.2_747-765
PHYPAV33_Pp3c5_3270V3.1_685-705
MARPO_Mapoly0088s0065.1.p_745-765
KLEFL_kfl00160_0160_v1.1_646-665
ARATH_AT1G74250.1_601-620
GINBI_Gb_36905_709-728
AZOFIhc_s0129.g048868_692-715
PENMA_pm004120g0030_686-709
ORYSAJA_LOC_Os12g31840.1_575-597
PYRYE_py10053.t1_644-667
GALSU_XP_005709343.1_473-496
GALPH_Rhodophyta-Galdieria_phlegrea.ESPRIT.254_448-471
OSTTA_jgi_Ostta1115_2_80655_e_gw1.88.941.1_5-24
OSTTA_jgi_Ostta1115_2_194318_fgenesh2_kg.unitig_81___483___TRINITY_DN376_c0_g1_i1_235-253
GRACH_PXF47706.1_55-78
GRACH_PXF42817.1_500-522
PORUM_Pum0130s0004.1.p_316-339
PORPU_evm.model.contig_3669.1_205-228
GRACH_PXF44133.1_162-187
CHOCR_CDF40112.1_146-171
PORPU_evm.model.contig_617.3_159-182
GINBI_Gb_41671_129-150
PORPU_evm.model.contig_535.4_56-76
Phragmoplastophyta
GINBI_Gb_21793_296-316
ORYSAJA_LOC_Os06g40960.1_362-382
ARATH_AT3G57670.1_301-321
PHYPAV33_Pp3c15_17790V3.1_305-325
PHYPAV33_Pp3c9_22230V3.1_310-330
ORYSAJA_LOC_Os09g10980.1_239-259
AZOFIhc_s0127.g048713_560-580
SPIMU_SM000097S24771_653-673
ORYSAJA_LOC_Os01g63980.1_300-320
ARATH_AT1G13290.1_186-207
GINBI_Gb_05632_298-318
MARPO_Mapoly0096s0050.1.p_367-387
ORYSAJA_LOC_Os09g31140.1_203-223
ORYSAJA_LOC_Os08g39390.1_352-372
ARATH_AT1G51220.1_262-282
ARATH_AT3G20880.1_340-360
ORYSAJA_LOC_Os09g13680.1_279-299
ARATH_AT1G08290.1_265-285
ANTAG_AagrBONN_evm.model.Sc2ySwM_344.1219.1_405-425
PORUM_Pum0238s0013.1.p_480-501
PORUM_Pum0172s0001.1.p_425-446
CALTU_IDg16773t1_179-201
GRACH_PXF42896.1_186-208
CALTU_IDg21130t1_35-54
CALTU_IDg13636t1_85-105
GRACH_PXF48773.1_104-127
CHOCR_CDF32423.1_33-56
CALTU_IDg14867t1_204-226
PORUM_Pum0068s0012.1.p_548-571
PYRYE_py04271.t1_587-610
PORPU_evm.model.contig_2044.6_517-538
PYRYE_py05845.t1_274-297
PORPU_evm.model.contig_462.18_405-428
CALTU_IDg2263t1_68-92
PHYPAV33_Pp3c11_480V3.1_136-160
ANTAG_AagrBONN_evm.model.Sc2ySwM_228.4391.1_229-253
MARPO_Mapoly0061s0023.1.p_130-154
PORPU_evm.model.contig_2077.17_485-508
PORUM_Pum1750s0001.1.p_1024-1047
PYRYE_py04491.t1_1040-1063
CHOCR_CDF39023.1_538-561
AZOFIhc_s1070.g096819_247-271
PYRYE_py04611.t1_406-429
PORUM_Pum0289s0006.1.p_417-440
PYRYE_py01069.t1_326-349
GRACH_PXF41604.1_151-174
GRACH_PXF43072.1_205-228
PYRYE_py05773.t1_344-366
PYRYE_py09539.t1_663-686
PORUM_Pum0161s0035.1.p_321-342
CALTU_IDg14867t1_290-309
PYRYE_py03739.t1_187-210
PORUM_Pum0746s0004.1.p_489-512
PORPU_evm.model.contig_3601.2_335-358
PYRYE_py01695.t1_278-300
CHRZO_Cz16g17050.t1_110-132
PYRYE_py11027.t1_149-169
ULVMU_UM055_0062.1_545-568
GONAV_comp45449_c0_seq1_6_ORF3_424_268-291
GINBI_Gb_37970_426-451
PORPU_evm.model.contig_3458.14_58-80
ORYSAJA_LOC_Os12g38960.1_71-92
PORUM_Pum0161s0035.1.p_232-251
PYRYE_py05773.t1_257-276
PORPU_evm.model.contig_666.5_432-451
CHOCR_CDF32272.1_197-221
KLEFL_kfl00734_0080_v1.1_357-381
AZOFIhc_s0019.g015022_371-393
SPIMU_SM000148S01039_204-221
SPIMU_SM000059S18695_201-224
SPIMU_SM002393S08021_255-278
CHLAT_Chrsp38S05549_315-338
CHABR_g66700.t2_747-768
ANTAG_AagrBONN_evm.model.Sc2ySwM_368.579.1_330-353
PORPU_evm.model.contig_2499.4_703-726
PORPU_evm.model.contig_2044.18_283-306
GRACH_PXF44686.1_106-129
PORPU_evm.model.contig_2035b.30_596-618
PORPU_evm.model.contig_4402.1_921-944
PYRYE_py04611.t1_464-487
PORUM_Pum0289s0006.1.p_477-498
PORUM_Pum0224s0004.1.p_854-877
PYRYE_py01069.t1_384-407
PORPU_evm.model.contig_3458.14_117-140
CHLAT_Chrsp35S05222_84-103
PORUM_Pum0734s0001.1.p_221-241
PYRYE_py11171.t1_236-258
PORPU_evm.model.contig_568.1_292-315
PORPU_evm.model.contig_3384.2_542-565
GRACH_PXF45050.1_178-201
GRACH_PXF49294.1_178-201
CALTU_IDg9365t1_293-316
PYRYE_py11221.t1_519-542
PORUM_Pum0266s0007.1.p_413-436
PORUM_Pum1321s0001.1.p_446-468
PYRYE_py10388.t1_476-498
CALTU_IDg3460t1_152-177
KLEFL_kfl00144_0210_v1.1_69-87
GONAV_comp24901_c0_seq1_6_ORF3_329_151-173
GONAV_comp24901_c0_seq1_6_ORF3_329_263-285
GRACH_PXF47283.1_173-189
GUITH_53031_64-82
AZOFIhc_s1063.g096483_304-326
PORUM_Pum0235s0016.1.p_189-211
PORUM_Pum0235s0005.1.p_156-178
PYRYE_py00825.t1_9-31

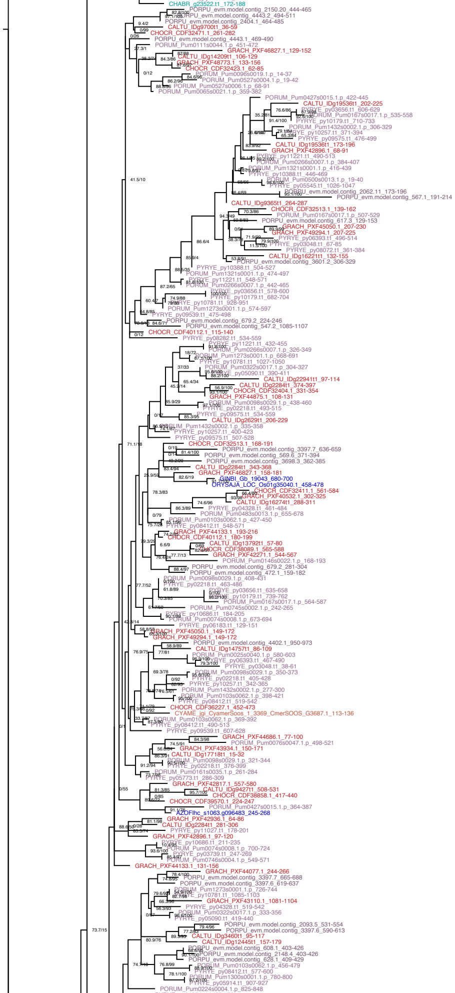

CHABR_g23522.t1_172-188
PORPU_evm.model.contig_2150.20_444-465
PORPU_evm.model.contig_4443.2_494-511
PORPU_evm.model.contig_2404.1_464-485
CALTU_IDg9700t1_36-59
CHOCR_CDF32471.1_261-282
PORPU_evm.model.contig_4443.1_469-490
PORUM_Pum0111s0044.1.p_451-472
GRACH_PXF46827.1_129-152
CALTU_IDg14209t1_106-129
GRACH_PXF48773.1_133-156
CHOCR_CDF32423.1_62-85
PORUM_Pum0096s0019.1.p_14-37
PORUM_Pum0527s0004.1.p_19-42
PORUM_Pum0527s0006.1.p_68-91
PORUM_Pum0065s0021.1.p_359-382
PORUM_Pum0427s0015.1.p_422-445
CALTU_IDg19536t1_202-225
PYRYE_py03656.t1_606-629
PORUM_Pum0167s0017.1.p_535-558
PYRYE_py10179.t1_710-733
PORUM_Pum1432s0002.1.p_306-329
PYRYE_py10257.t1_371-394
PYRYE_py09575.t1_476-499
CALTU_IDg19536t1_173-196
GRACH_PXF42896.1_68-91
PYRYE_py11221.t1_490-513
PORUM_Pum0266s0007.1.p_384-407
PORUM_Pum1321s0001.1.p_416-439
PYRYE_py10388.t1_446-469
PORUM_Pum0500s0013.1.p_19-40
PYRYE_py05545.t1_1026-1047
PORPU_evm.model.contig_2062.11_173-196
PORPU_evm.model.contig_567.1_191-214
CALTU_IDg9365t1_264-287
CHOCR_CDF32513.1_139-162
PORUM_Pum0167s0017.1.p_507-529
PORPU_evm.model.contig_617.3_129-153
GRACH_PXF45050.1_207-230
GRACH_PXF49294.1_207-225
PYRYE_py06393.t1_496-514
PYRYE_py03048.t1_67-85
PYRYE_py08072.t1_361-384
CALTU_IDg16221t1_132-155
PORPU_evm.model.contig_3601.2_306-329
PYRYE_py10388.t1_504-527
PORUM_Pum1321s0001.1.p_474-497
PYRYE_py11221.t1_548-571
PORUM_Pum0266s0007.1.p_442-465
PYRYE_py03656.t1_578-600
PYRYE_py10179.t1_682-704
PYRYE_py10781.t1_928-951
PORUM_Pum1273s0001.1.p_574-597
PYRYE_py09539.t1_475-498
PORPU_evm.model.contig_679.2_224-246
PORPU_evm.model.contig_547.2_1085-1107
CHOCR_CDF40112.1_115-140
PYRYE_py08282.t1_534-559
PYRYE_py11221.t1_432-455
PORUM_Pum0266s0007.1.p_326-349
PORUM_Pum1273s0001.1.p_668-691
PYRYE_py10781.t1_1027-1050
PORUM_Pum0322s0017.1.p_304-327
PYRYE_py05090.t1_390-411
CALTU_IDg22941t1_97-114
CALTU_IDg2284t1_374-397
CHOCR_CDF32404.1_331-354
GRACH_PXF44875.1_108-131
PORUM_Pum0098s0029.1.p_438-460
PYRYE_py02218.t1_493-515
PYRYE_py09575.t1_534-559
CALTU_IDg2629t1_206-229
PORUM_Pum1432s0002.1.p_335-358
PYRYE_py10257.t1_400-423
PYRYE_py09575.t1_507-528
CHOCR_CDF32513.1_168-191
PORPU_evm.model.contig_3397.7_636-659
PORPU_evm.model.contig_569.6_371-394
PORPU_evm.model.contig_3698.3_362-385
CALTU_IDg2284t1_343-368
GRACH_PXF46827.1_158-181
GINBI_Gb_19043_680-700
ORYSAJA_LOC_Os01g35040.1_458-478
CHOCR_CDF32411.1_561-584
GRACH_PXF40532.1_302-325
CALTU_IDg16274t1_288-311
PYRYE_py04328.t1_461-484
PORUM_Pum0483s0013.1.p_655-678
PORUM_Pum0103s0062.1.p_427-450
PYRYE_py08412.t1_548-571
GRACH_PXF44133.1_193-216
CHOCR_CDF40112.1_180-199
CALTU_IDg13792t1_57-80
CHOCR_CDF38089.1_565-588
GRACH_PXF42271.1_544-567
PORUM_Pum0146s0022.1.p_168-193
PORPU_evm.model.contig_679.2_281-304
PORPU_evm.model.contig_472.1_159-182
PORUM_Pum0098s0029.1.p_408-431
PYRYE_py02218.t1_463-486
PYRYE_py03656.t1_635-658
PYRYE_py10179.t1_739-762
PORUM_Pum0167s0017.1.p_564-587
PORUM_Pum0745s0002.1.p_242-265
PYRYE_py10686.t1_184-205
PORUM_Pum0074s0008.1.p_673-694
PYRYE_py06183.t1_129-151
GRACH_PXF45050.1_149-172
GRACH_PXF49294.1_149-172
PORPU_evm.model.contig_4402.1_950-973
CALTU_IDg14757t1_86-109
PORUM_Pum0025s0040.1.p_580-603
PYRYE_py06393.t1_467-490
PYRYE_py03048.t1_38-61
PORUM_Pum0098s0029.1.p_350-373
PYRYE_py02218.t1_405-428
PYRYE_py10257.t1_342-365
PORUM_Pum1432s0002.1.p_277-300
PORUM_Pum0103s0062.1.p_398-421
PYRYE_py08412.t1_519-542
CHOCR_CDF36227.1_452-473
CYAME_jgi_CymerSoos_1_3369_CmerSOOS_G3687.1_113-136
PORUM_Pum0103s0062.1.p_369-392
PYRYE_py08412.t1_490-513
PYRYE_py09539.t1_607-628
GRACH_PXF44686.1_77-100
PORUM_Pum0076s0047.1.p_498-521
GRACH_PXF43934.1_150-171
CALTU_IDg17718t1_15-32
PORUM_Pum0098s0029.1.p_321-344
PYRYE_py02218.t1_376-399
PORUM_Pum0161s0035.1.p_261-284
PYRYE_py05773.t1_286-309
GRACH_PXF42817.1_557-580
CALTU_IDg9427t1_508-531
CHOCR_CDF38858.1_417-440
CHOCR_CDF39570.1_224-247
PORUM_Pum0427s0015.1.p_364-387
AZOFIhc_s1063.g096483_245-268
GRACH_PXF42936.1_64-86
CALTU_IDg2284t1_281-306
PYRYE_py11027.t1_178-201
GRACH_PXF42896.1_97-120
PYRYE_py10686.t1_211-235
PORUM_Pum0074s0008.1.p_700-724
PYRYE_py03739.t1_247-269
PORUM_Pum0746s0004.1.p_549-571
GRACH_PXF44133.1_131-156
GRACH_PXF44077.1_244-266
PORPU_evm.model.contig_3397.7_665-688
PORPU_evm.model.contig_3397.6_619-637
PORUM_Pum1273s0001.1.p_726-744
PYRYE_py10781.t1_1085-1103
GRACH_PXF43110.1_1081-1104
PYRYE_py04328.t1_519-542
PORUM_Pum0322s0017.1.p_333-356
PYRYE_py05090.t1_419-440
PORPU_evm.model.contig_2093.5_531-554
PORPU_evm.model.contig_3397.6_590-613
CALTU_IDg3460t1_95-117
CALTU_IDg12445t1_157-179
PORPU_evm.model.contig_608.1_403-426
PORPU_evm.model.contig_2148.4_403-426
PORPU_evm.model.contig_628.1_409-429
PORUM_Pum0103s0062.1.p_456-479
PYRYE_py08412.t1_577-600
PORUM_Pum1300s0001.1.p_780-800
PYRYE_py05914.t1_907-927
PORUM_Pum0224s0004.1.p_825-848

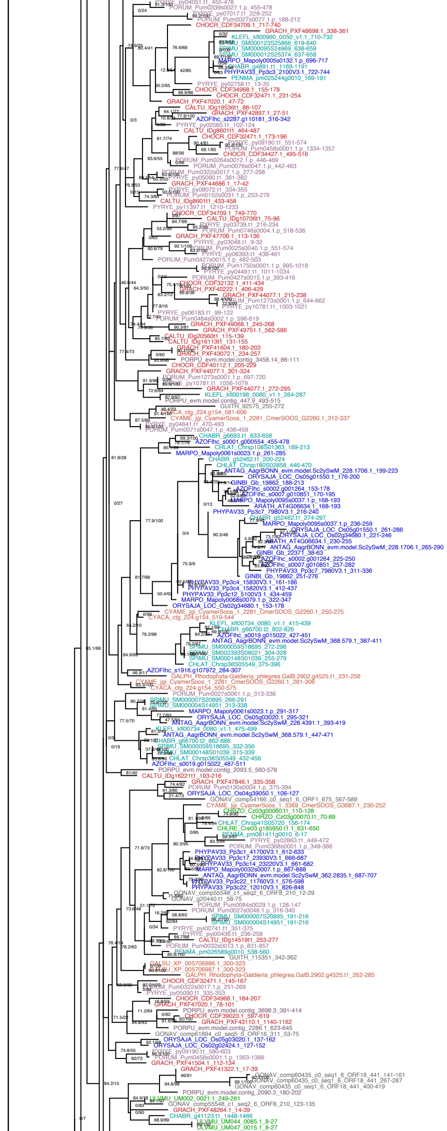
PORUM_Pum0339s0027.1.p_455-478
PYRYE_py07017.t1_228-252
PORUM_Pum0027s0077.1.p_188-212
CHOCR_CDF34709.1_717-740
GRACH_PXF48698.1_338-361
KLEFL_kfl00960_0050_v1.1_710-732
SPIMU_SM000123S25866_619-640
SPIMU_SM000095S24969_638-659
SPIMU_SM000012S25374_637-658
MARPO_Mapoly0005s0132.1.p_696-717
CHABR_g4891.t1_1169-1191
PHYPAV33_Pp3c3_2100V3.1_722-744
PENMA_pm025244g0010_169-191
PYRYE_py02758.t1_13-35
CHOCR_CDF34968.1_155-178
CHOCR_CDF32471.1_231-254
GRACH_PXF47020.1_47-72
CALTU_IDg19536t1_86-107
GRACH_PXF42897.1_27-51
AZOFIhc_s2287.g110181_316-342
PYRYE_py02565.t1_102-124
CALTU_IDg8601t1_464-487
CHOCR_CDF32471.1_173-196
PYRYE_py09190.t1_551-574
PORUM_Pum0458s0001.1.p_1334-1357
CHOCR_CDF34427.1_495-516
PORUM_Pum0264s0012.1.p_446-469
PORUM_Pum0076s0047.1.p_442-463
PORUM_Pum0322s0017.1.p_277-298
PYRYE_py05090.t1_361-382
GRACH_PXF44686.1_17-42
PYRYE_py08072.t1_334-355
PORUM_Pum0152s0031.1.p_253-276
CALTU_IDg8601t1_433-458
PYRYE_py11397.t1_1210-1233
CHOCR_CDF34709.1_749-770
CALTU_IDg10706t1_75-96
PYRYE_py03739.t1_216-234
PORUM_Pum0746s0004.1.p_518-536
GRACH_PXF47706.1_113-136
PYRYE_py03048.t1_9-32
PORUM_Pum0025s0040.1.p_551-574
PYRYE_py06393.t1_438-461
PORUM_Pum0427s0015.1.p_482-503
PORUM_Pum1750s0001.1.p_995-1018
PYRYE_py04491.t1_1011-1034
PORUM_Pum0427s0015.1.p_393-416
CHOCR_CDF32132.1_411-434
GRACH_PXF40222.1_406-429
GRACH_PXF44077.1_215-238
PORUM_Pum1273s0001.1.p_644-662
PYRYE_py10781.t1_1003-1021
PYRYE_py06183.t1_99-122
PORUM_Pum0484s0002.1.p_596-619
GRACH_PXF49068.1_245-268
GRACH_PXF49751.1_562-586
CALTU_IDg20560t1_115-139
CALTU_IDg16113t1_131-155
GRACH_PXF41604.1_180-203
GRACH_PXF43072.1_234-257
PORPU_evm.model.contig_3458.14_86-111
CHOCR_CDF40112.1_205-229
GRACH_PXF44077.1_301-324
PORUM_Pum1273s0001.1.p_697-720
PYRYE_py10781.t1_1056-1079
GRACH_PXF44077.1_272-295
KLEFL_kfl00198_0060_v1.1_264-287
PORPU_evm.model.contig_447.9_493-515
GUITH_92575_255-272
CYACA_ctg_224.g154_581-606
CYAME_jgi_CyamerSoos_1_2281_CmerSOOS_G2260.1_312-337
PYRYE_py04641.t1_470-493
PORUM_Pum0071s0047.1.p_436-459
CHABR_g6693.t1_633-658
AZOFIhc_s0001.g000554_455-478
CHLAT_Chrsp108S01363_189-213
MARPO_Mapoly0061s0023.1.p_261-285
CHABR_g52462.t1_200-224
CHLAT_Chrsp180S02858_446-470
ANTAG_AagrBONN_evm.model.Sc2ySwM_228.1706.1_199-223
ORYSAJA_LOC_Os05g01550.1_176-200
GINBI_Gb_19862_188-213
AZOFIhc_s0002.g001264_153-178
AZOFIhc_s0007.g010851_170-195
MARPO_Mapoly0095s0037.1.p_168-193
ARATH_AT4G06634.1_168-193
PHYPAV33_Pp3c7_7980V3.1_216-240
CHABR_g52462.t1_274-297
MARPO_Mapoly0095s0037.1.p_236-259
ORYSAJA_LOC_Os05g01550.1_261-286
ORYSAJA_LOC_Os02g34680.1_221-246
ARATH_AT4G06634.1_230-255
ANTAG_AagrBONN_evm.model.Sc2ySwM_228.1706.1_265-290
GINBI_Gb_22371_38-63
AZOFIhc_s0002.g001264_225-250
AZOFIhc_s0007.g010851_257-282
PHYPAV33_Pp3c7_7980V3.1_311-336
GINBI_Gb_19862_251-276
PHYPAV33_Pp3c4_15830V3.1_161-186
PHYPAV33_Pp3c4_15820V3.1_412-437
PHYPAV33_Pp3c12_5100V3.1_434-459
MARPO_Mapoly0068s0079.1.p_322-347
ORYSAJA_LOC_Os02g34680.1_153-178
CYAME_jgi_CyamerSoos_1_2281_CmerSOOS_G2260.1_250-275
CYACA_ctg_224.g154_519-544
KLEFL_kfl00734_0080_v1.1_415-439
CHABR_g66700.t2_802-826
AZOFIhc_s0019.g015022_427-451
ANTAG_AagrBONN_evm.model.Sc2ySwM_368.579.1_387-411
SPIMU_SM000059S18695_272-296
SPIMU_SM000239S08021_304-328
SPIMU_SM000148S01039_255-279
CHLAT_Chrsp38S05549_375-396
AZOFIhc_s1916.g107972_284-307
GALPH_Rhodophyta-Galdieria_phlegrea.GalB.2902.g4525.t1_231-256
CYAME_jgi_CyamerSoos_1_2281_CmerSOOS_G2260.1_281-306
CYACA_ctg_224.g154_550-575
PORUM_Pum0027s0061.1.p_313-336
SPIMU_SM000007S20895_266-291
SPIMU_SM000004S14951_313-338
MARPO_Mapoly0061s0023.1.p_291-317
ORYSAJA_LOC_Os05g03020.1_295-321
ANTAG_AagrBONN_evm.model.Sc2ySwM_228.4391.1_393-419
KLEFL_kfl00734_0080_v1.1_475-499
ANTAG_AagrBONN_evm.model.Sc2ySwM_368.579.1_447-471
CHABR_g66700.t2_862-886
SPIMU_SM000059S18695_332-356
SPIMU_SM000148S01039_315-339
CHLAT_Chrsp38S05549_432-456
AZOFIhc_s0019.g015022_487-511
PORPU_evm.model.contig_2093.5_560-578
CALTU_IDg16221t1_193-216
GRACH_PXF47846.1_335-358
PORUM_Pum0130s0004.1.p_375-394
ORYSAJA_LOC_Os04g39050.1_106-127
GONAV_comp54166_c0_seq1_6_ORF1_675_567-589
CYAME_jgi_CyamerSoos_1_3369_CmerSOOS_G3687.1_230-252
CHRZO_Cz03g00060.t1_110-128
CHRZO_Cz03g00070.t1_70-89
CHLAT_Chrsp41S05720_156-174
CHLRE_Cre03.g185950.t1.1_631-650
PENMA_pm081411g0010_6-17
PYRYE_py02863.t1_449-472
PORUM_Pum0368s0001.1.p_349-366
PHYPAV33_Pp3c1_41700V3.1_612-633
PHYPAV33_Pp3c17_23930V3.1_666-687
PHYPAV33_Pp3c14_23220V3.1_661-682
MARPO_Mapoly0032s0007.1.p_867-888
ANTAG_AagrBONN_evm.model.Sc2ySwM_362.2835.1_687-707
PHYPAV33_Pp3c22_11760V3.1_576-598
PHYPAV33_Pp3c22_12010V3.1_826-848
GONAV_comp55548_c1_seq2_6_ORF8_210_12-29
GONAV_g20440.t1_58-75
PORUM_Pum0084s0029.1.p_128-147
PORUM_Pum0027s0048.1.p_316-340
SPIMU_SM000007S20895_191-216
SPIMU_SM000004S14951_191-216
PYRYE_pyi00741.t1_351-375
PYRYE_pyi00436.t1_236-258
CALTU_IDg14519t1_253-277
PORUM_Pum0032s0013.1.p_831-857
PENMA_pm026589g0010_538-560
GUITH_115351_342-362
GALSU_XP_005706986.1_300-323
GALSU_XP_005706987.1_300-323
GALPH_Rhodophyta-Galdieria_phlegrea.GalB.2902.g4525.t1_262-285
CHOCR_CDF32471.1_145-167
PORUM_Pum0322s0017.1.p_251-269
PYRYE_py05090.t1_335-353
CHOCR_CDF34968.1_184-207
GRACH_PXF47020.1_78-101
PORPU_evm.model.contig_3698.3_391-414
CHOCR_CDF39023.1_597-619
GRACH_PXF43110.1_1140-1162
PORPU_evm.model.contig_2286.1_623-645
GONAV_comp61894_c0_seq5_6_ORF16_311_53-75
ORYSAJA_LOC_Os05g03020.1_137-162
ORYSAJA_LOC_Os02g02424.1_127-152
PYRYE_py09190.t1_580-603
PORUM_Pum0458s0001.1.p_1363-1386
GRACH_PXF41504.1_112-134
GRACH_PXF41322.1_17-39
GONAV_comp60435_c0_seq1_6_ORF18_441_141-161
GONAV_comp60435_c0_seq1_6_ORF18_441_267-287
GONAV_comp60435_c0_seq1_6_ORF18_441_400-419
PORPU_evm.model.contig_2090.3_180-202
ULVMU_UM002_0021.1_249-261
GONAV_comp55548_c1_seq2_6_ORF8_210_123-135
GRACH_PXF48264.1_14-39
CHABR_g41123.t1_1448-1466
ULVMU_UM044_0085.1_8-27
ULVMU_UM047_0015.1_8-27

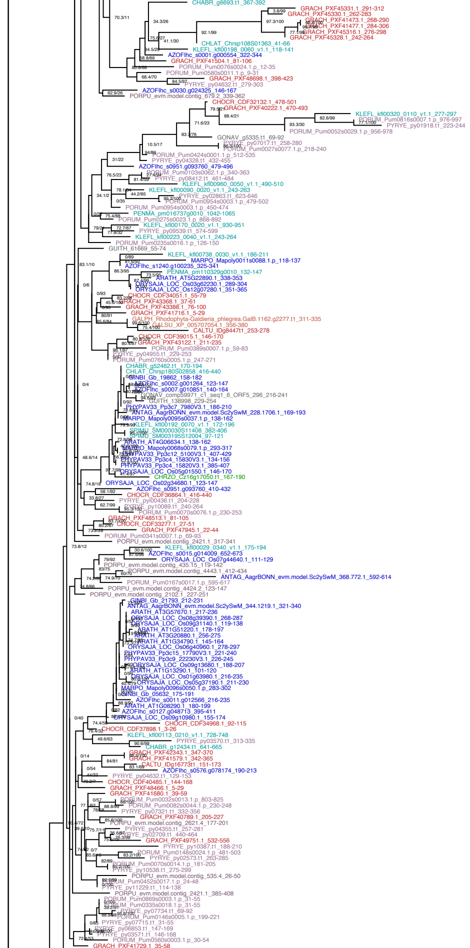

CHABR_g6693.t1_367-392
GRACH_PXF45331.1_291-312
GRACH_PXF45330.1_262-283
GRACH_PXF41473.1_268-290
GRACH_PXF41477.1_284-306
GRACH_PXF45316.1_276-298
GRACH_PXF45328.1_242-264
CHLAT_Chrsp108S01363_41-66
KLEFL_kfl00198_0060_v1.1_118-141
AZOFlhc_s0001.g000554_322-344
GRACH_PXF41504.1_81-106
PORUM_Pum0076s0024.1.p_12-35
PORUM_Pum0580s0011.1.p_9-31
GRACH_PXF48698.1_398-423
PYRYE_py04632.t1_279-303
AZOFlhc_s0030.g024325_146-167
PORPU_evm.model.contig_679.2_339-362
CHOCR_CDF32132.1_478-501
GRACH_PXF40222.1_470-493
KLEFL_kfl00320_0110_v1.1_277-297
PORUM_Pum0816s0007.1.p_976-997
PYRYE_py01918.t1_223-244
PORUM_Pum0052s0029.1.p_956-978
GONAV_g5335.t1_69-92
PYRYE_py07017.t1_258-280
PORUM_Pum0027s0077.1.p_218-240
PORUM_Pum0424s0001.1.p_512-535
PYRYE_py04328.t1_432-455
AZOFlhc_s0951.g093760_479-496
PORUM_Pum0103s0062.1.p_340-363
PYRYE_py08412.t1_461-484
KLEFL_kfl00960_0050_v1.1_490-510
KLEFL_kfl00090_0020_v1.1_243-263
PYRYE_py02863.t1_623-646
PORUM_Pum0954s0003.1.p_479-502
PORUM_Pum0954s0003.1.p_450-474
PENMA_pm016737g0010_1042-1065
PORUM_Pum0275s0023.1.p_868-892
KLEFL_kfl00170_0020_v1.1_930-951
PYRYE_py09539.t1_574-599
KLEFL_kfl00223_0040_v1.1_243-264
PORUM_Pum0235s0016.1.p_126-150
GUITH_61669_55-74
KLEFL_kfl00738_0030_v1.1_186-211
MARPO_Mapoly0011s0088.1.p_118-137
AZOFlhc_s1240.g100235_325-341
PENMA_pm110329g0010_132-147
ARATH_AT5G22890.1_338-353
ORYSAJA_LOC_Os03g62230.1_289-304
ORYSAJA_LOC_Os12g07280.1_351-365
CHOCR_CDF34051.1_55-79
GRACH_PXF43368.1_37-61
GRACH_PXF43388.1_76-100
GRACH_PXF41716.1_5-29
GALPH_Rhodophyta-Galdieria_phlegrea.GalB.1162.g2277.t1_311-335
GALSU_XP_005707054.1_356-380
CALTU_IDg8447t1_253-278
CHOCR_CDF39015.1_146-170
GRACH_PXF43122.1_211-235
PORUM_Pum0389s0007.1.p_59-83
PYRYE_py04955.t1_229-253
PORUM_Pum0760s0005.1.p_247-271
CHABR_g52462.t1_170-194
CHLAT_Chrsp180S02858_416-440
GINBI_Gb_19862_158-182
AZOFlhc_s0002.g001264_123-147
AZOFlhc_s0007.g010851_140-164
GONAV_comp59971_c1_seq1_6_ORF5_296_216-241
GUITH_138998_229-254
PHYPAV33_Pp3c7_7980V3.1_186-210
ANTAG_AagrBONN_evm.model.Sc2ySwM_228.1706.1_169-193
MARPO_Mapoly0095s0037.1.p_138-162
KLEFL_kfl00192_0070_v1.1_172-196
SPIMU_SM000030S11408_382-406
SPIMU_SM003195S12004_97-121
ARATH_AT4G06634.1_138-162
MARPO_Mapoly0068s0079.1.p_293-317
PHYPAV33_Pp3c12_5100V3.1_407-429
PHYPAV33_Pp3c4_15830V3.1_134-156
PHYPAV33_Pp3c4_15820V3.1_385-407
ORYSAJA_LOC_Os05g01550.1_146-170
CHRZO_Cz16g17050.t1_167-190
ORYSAJA_LOC_Os02g34680.1_123-147
AZOFlhc_s0951.g093760_410-432
CHOCR_CDF36864.1_416-440
PYRYE_pyi00436.t1_204-228
PYRYE_py10089.t1_240-264
PORUM_Pum0070s0076.1.p_230-253
GRACH_PXF48513.1_81-105
CHOCR_CDF33277.1_27-51
GRACH_PXF47945.1_22-44
PORUM_Pum0341s0007.1.p_69-93
PORPU_evm.model.contig_2421.1_317-341
KLEFL_kfl00029_0340_v1.1_175-194
AZOFlhc_s0015.g014009_652-673
ORYSAJA_LOC_Os07g44640.1_111-129
PORPU_evm.model.contig_435.15_119-142
PORPU_evm.model.contig_4443.1_412-434
ANTAG_AagrBONN_evm.model.Sc2ySwM_368.772.1_592-614
PORUM_Pum0167s0017.1.p_595-617
PORPU_evm.model.contig_4424.2_123-147
PORPU_evm.model.contig_2102.1_227-251
GINBI_Gb_21793_212-231
ANTAG_AagrBONN_evm.model.Sc2ySwM_344.1219.1_321-340
ARATH_AT3G57670.1_217-236
ORYSAJA_LOC_Os08g39390.1_268-287
ORYSAJA_LOC_Os09g31140.1_119-138
ARATH_AT1G51220.1_178-197
ARATH_AT3G20880.1_256-275
ARATH_AT1G34790.1_145-164
ORYSAJA_LOC_Os06g40960.1_278-297
PHYPAV33_Pp3c15_17790V3.1_221-240
PHYPAV33_Pp3c9_22230V3.1_226-245
ORYSAJA_LOC_Os09g13680.1_188-207
ARATH_AT1G13290.1_101-120
ORYSAJA_LOC_Os01g63980.1_216-235
ORYSAJA_LOC_Os05g37190.1_211-230
MARPO_Mapoly0096s0050.1.p_283-302
GINBI_Gb_05632_175-191
AZOFlhc_s0011.g012566_216-235
ARATH_AT1G08290.1_180-199
AZOFlhc_s0127.g048713_395-411
ORYSAJA_LOC_Os09g10980.1_155-174
CHOCR_CDF34968.1_92-115
CHOCR_CDF37898.1_3-26
KLEFL_kfl00113_0210_v1.1_728-748
PYRYE_py03570.t1_313-335
CHABR_g12434.t1_641-665
GRACH_PXF42343.1_347-370
GRACH_PXF41579.1_342-365
CALTU_IDg16773t1_151-173
AZOFlhc_s0576.g078174_190-213
PYRYE_py04632.t1_129-153
CHOCR_CDF40485.1_144-168
GRACH_PXF48466.1_5-29
GRACH_PXF41680.1_39-59
PORUM_Pum0032s0013.1.p_803-825
PORUM_Pum0082s0044.1.p_230-248
PYRYE_py07321.t1_332-356
GRACH_PXF40789.1_205-227
PORPU_evm.model.contig_2621.4_177-201
PYRYE_py04355.t1_257-281
PYRYE_py02709.t1_440-464
GRACH_PXF49751.1_532-556
PYRYE_py10387.t1_188-210
PORUM_Pum0148s0024.1.p_481-503
PYRYE_py02573.t1_263-285
PORUM_Pum0070s0014.1.p_181-205
PYRYE_py10538.t1_275-299
PORPU_evm.model.contig_535.4_26-50
[illegible]_Pum0452s0017.1.p_24-48
PYRYE_py11229.t1_114-138
PORPU_evm.model.contig_2421.1_385-408
PORUM_Pum0869s0003.1.p_31-55
PORUM_Pum0335s0018.1.p_31-55
PYRYE_py07734.t1_69-92
PORUM_Pum0146s0005.1.p_199-221
PYRYE_py07715.t1_31-55
PYRYE_py06853.t1_147-169
PYRYE_py03571.t1_146-168
PORUM_Pum0560s0003.1.p_30-54
GRACH_PXF41729.1_35-58

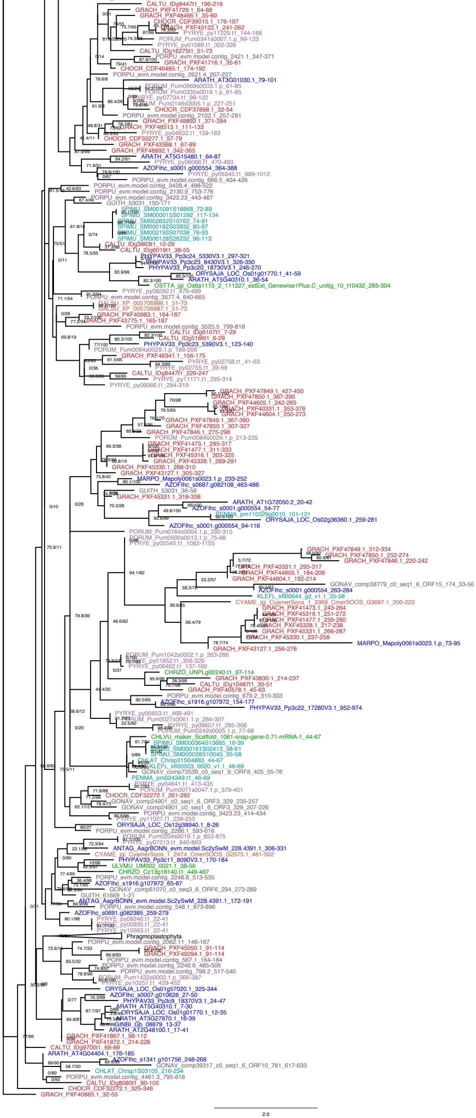

CALTU_IDg8447t1_196-219
GRACH_PXF41729.1_64-88
GRACH_PXF48466.1_35-60
CHOCR_CDF39015.1_176-197
GRACH_PXF43122.1_241-262
PYRYE_py11229.t1_144-166
PORUM_Pum0341s0007.1.p_99-123
PYRYE_py01089.t1_302-326
CALTU_IDg16275t1_51-73
PORPU_evm.model.contig_2421.1_347-371
GRACH_PXF41716.1_35-61
CHOCR_CDF40485.1_174-192
PORPU_evm.model.contig_2621.4_207-227
ARATH_AT3G01030.1_79-101
PORUM_Pum0869s0003.1.p_61-85
PORUM_Pum0335s0018.1.p_61-85
PYRYE_py07734.t1_98-122
PORUM_Pum0146s0005.1.p_227-251
CHOCR_CDF37898.1_32-54
PORPU_evm.model.contig_2102.1_257-281
GRACH_PXF48892.1_371-394
GRACH_PXF48513.1_111-133
PYRYE_py04632.t1_159-183
CHOCR_CDF33277.1_57-79
GRACH_PXF43368.1_67-89
GRACH_PXF48892.1_342-365
ARATH_AT5G15480.1_64-87
PYRYE_py06066.t1_470-493
AZOFIhc_s0001.g000554_364-388
PYRYE_py05545.t1_989-1012
PORPU_evm.model.contig_666.5_404-426
PORPU_evm.model.contig_3428.4_498-522
PORPU_evm.model.contig_2130.9_753-776
PORPU_evm.model.contig_3423.23_443-467
GUITH_53031_150-171
SPIMU_SM001091S18868_72-89
SPIMU_SM000015S01292_117-134
SPIMU_SM002832S10762_74-91
SPIMU_SM000182S03932_80-97
SPIMU_SM002193S07038_76-93
SPIMU_SM000128S26232_96-113
CALTU_IDg3863t1_12-29
CALTU_IDg6019t1_38-55
PHYPAV33_Pp3c24_5330V3.1_297-321
PHYPAV33_Pp3c23_8430V3.1_326-350
PHYPAV33_Pp3c20_18730V3.1_246-270
ORYSAJA_LOC_Os01g01770.1_41-59
ARATH_AT5G40310.1_36-54
OSTTA_jgi_Ostta1115_2_111227_estExt_Genewise1Plus.C_unitig_10_t10432_285-304
PYRYE_py08282.t1_476-499
PORPU_evm.model.contig_3577.4_640-665
GALSU_XP_005706986.1_51-73
GALSU_XP_005706987.1_51-73
GRACH_PXF40983.1_164-187
GRACH_PXF43775.1_165-187
PORPU_evm.model.contig_3525.5_799-818
CALTU_IDg6107t1_7-29
CALTU_IDg5186t1_6-29
PHYPAV33_Pp3c23_5390V3.1_123-140
PORUM_Pum0084s0029.1.p_188-206
GRACH_PXF46341.1_156-175
PYRYE_py02758.t1_41-63
PYRYE_py02755.t1_39-59
CALTU_IDg8447t1_226-247
PYRYE_py11171.t1_295-314
PYRYE_py06066.t1_294-319
GRACH_PXF47849.1_427-450
GRACH_PXF47850.1_367-390
GRACH_PXF44605.1_242-265
GRACH_PXF40331.1_353-376
GRACH_PXF44604.1_250-273
GRACH_PXF47849.1_367-390
GRACH_PXF47850.1_307-327
GRACH_PXF47846.1_275-298
PORUM_Pum0084s0029.1.p_213-235
GRACH_PXF41473.1_295-317
GRACH_PXF41477.1_311-333
GRACH_PXF45316.1_303-325
GRACH_PXF45328.1_269-291
GRACH_PXF45330.1_288-310
GRACH_PXF43127.1_305-327
MARPO_Mapoly0061s0023.1.p_233-252
AZOFIhc_s0687.g082108_463-486
GUITH_53031_36-58
GRACH_PXF45331.1_318-338
ARATH_AT1G72050.2_20-42
AZOFIhc_s0001.g000554_54-77
PENMA_pm110329g0010_101-121
ORYSAJA_LOC_Os02g36360.1_259-281
AZOFIhc_s0001.g000554_94-116
PORUM_Pum0764s0004.1.p_292-315
PORUM_Pum0500s0013.1.p_75-98
PYRYE_py05545.t1_1082-1105
GRACH_PXF47849.1_312-334
GRACH_PXF47850.1_252-274
GRACH_PXF47846.1_220-242
GRACH_PXF40331.1_295-317
GRACH_PXF44605.1_184-206
GRACH_PXF44604.1_192-214
GONAV_comp38779_c0_seq1_6_ORF15_174_33-56
AZOFIhc_s0001.g000554_263-284
KLEFL_kfl00644_g2_v1.1_35-58
CYAME_jgi_CyamerSoos_1_3369_CmerSOOS_G3687.1_200-223
GRACH_PXF41473.1_243-264
GRACH_PXF45316.1_251-272
GRACH_PXF41477.1_259-280
GRACH_PXF45328.1_217-238
GRACH_PXF45331.1_266-287
GRACH_PXF45330.1_237-258
MARPO_Mapoly0061s0023.1.p_73-95
GRACH_PXF43127.1_256-276
PORUM_Pum1042s0002.1.p_263-286
PYRYE_py01852.t1_306-329
PYRYE_py06462.t1_137-160
CHRZO_UNPLg00240.t1_97-114
GRACH_PXF43830.1_214-237
CALTU_IDg10487t1_30-51
GRACH_PXF40578.1_45-63
PORPU_evm.model.contig_679.2_310-333
AZOFIhc_s1916.g107972_154-177
PHYPAV33_Pp3c22_17280V3.1_952-974
PYRYE_py00653.t1_468-491
PORUM_Pum0027s0061.1.p_284-307
PYRYE_py09607.t1_285-306
PORUM_Pum0242s0005.1.p_77-98
CHLVU_maker_Scaffold_1061-snap-gene-0.71-mRNA-1_44-67
SPIMU_SM000364S13665_16-39
SPIMU_SM000161S02413_38-61
SPIMU_SM000028S10045_35-58
CHLAT_Chrsp31S04883_44-67
KLEFL_kfl00503_0020_v1.1_46-69
GONAV_comp73539_c0_seq1_6_ORF8_405_55-78
PENMA_pm024349.t1_46-69
PYRYE_py04641.t1_413-435
PORUM_Pum0071s0047.1.p_379-401
CHOCR_CDF32272.1_261-282
GONAV_comp24901_c0_seq1_6_ORF3_329_235-257
GONAV_comp24901_c0_seq1_6_ORF3_329_207-226
PORPU_evm.model.contig_3423.23_414-434
PYRYE_py11027.t1_238-255
ORYSAJA_LOC_Os12g38940.1_8-26
PORPU_evm.model.contig_2286.1_593-616
PORUM_Pum0204s0019.1.p_852-875
PYRYE_py07213.t1_840-863
ANTAG_AagrBONN_evm.model.Sc2ySwM_228.4391.1_306-331
CYAME_jgi_CyamerSoos_1_2474_CmerSOOS_G2575.1_481-502
PHYPAV33_Pp3c11_8090V3.1_170-184
ULVMU_UM002_0021.1_38-56
CHRZO_Cz13g18140.t1_449-467
PORPU_evm.model.contig_2246.6_513-535
AZOFIhc_s1916.g107972_65-87
GONAV_comp61070_c0_seq3_6_ORF6_294_273-289
GUITH_61669_1-21
ANTAG_AagrBONN_evm.model.Sc2ySwM_228.4391.1_172-191
PORPU_evm.model.contig_548.1_873-896
AZOFIhc_s0691.g082385_259-279
PYRYE_py09246.t1_22-41
PYRYE_py00935.t1_22-41
PYRYE_py10563.t1_22-41
Phragmoplastophyta
PORPU_evm.model.contig_2062.11_146-167
GRACH_PXF45050.1_91-114
GRACH_PXF49294.1_91-114
PORPU_evm.model.contig_567.1_164-184
PORPU_evm.model.contig_2246.6_485-506
PORPU_evm.model.contig_798.2_517-540
PORUM_Pum1432s0002.1.p_366-387
PYRYE_py10257.t1_429-452
ORYSAJA_LOC_Os01g57020.1_325-344
AZOFIhc_s0007.g010626_27-50
PHYPAV33_Pp3c9_18370V3.1_24-47
ARATH_AT5G40310.1_7-30
ORYSAJA_LOC_Os01g01770.1_12-35
ARATH_AT3G27970.1_16-39
GINBI_Gb_08879_13-37
ARATH_AT2G48100.1_17-41
GRACH_PXF41867.1_98-112
GRACH_PXF41872.1_214-228
CALTU_IDg9700t1_68-89
ARATH_AT4G04404.1_176-185
AZOFIhc_s1341.g101756_248-268
GONAV_comp39317_c0_seq1_6_ORF10_781_617-633
CHLAT_Chrsp1S03105_216-234
PORPU_evm.model.contig_4461.3_795-818
CALTU_IDg8080t1_90-105
CHOCR_CDF32272.1_325-346
GRACH_PXF40865.1_32-55
2.0

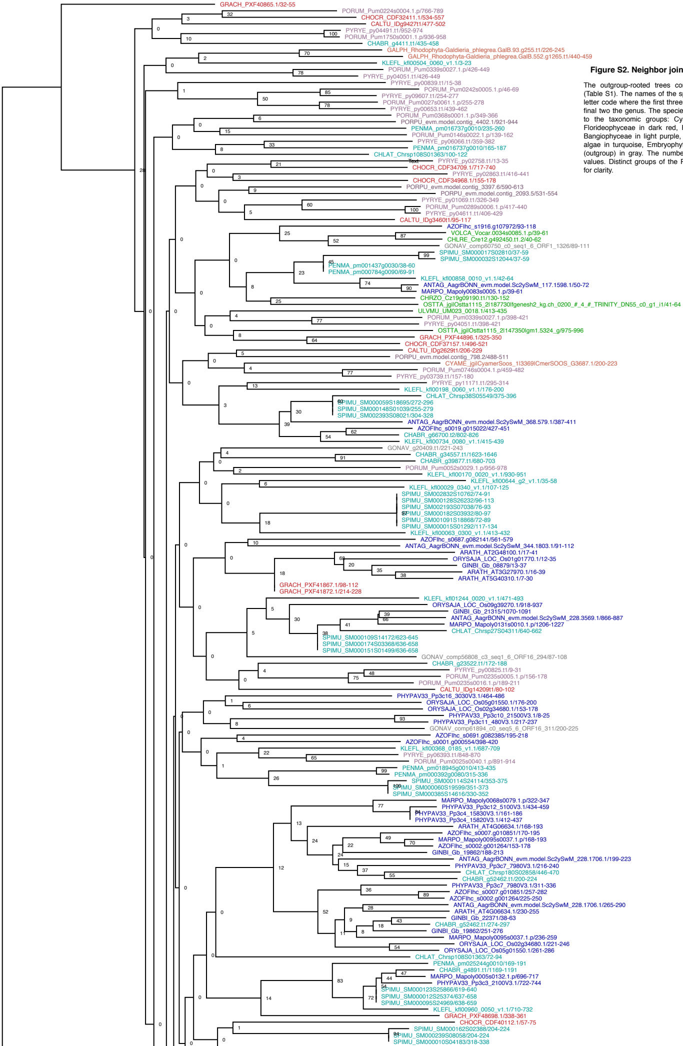

Figure S2. Neighbor joining tree of the C2H2 family.

The outgroup-rooted trees contain sequences from 30 species (Table S1). The names of the species are abbreviated using a five-letter code where the first three letters indicate the species and the final two the genus. The species names are color-coded according to the taxonomic groups: Cyanidiales are shown in light red, Florideophyceae in dark red, Porphyridiophyceae in dark purple, Bangiophyceae in light purple, Chlorophyta in green, streptophyte algae in turquoise, Embryophytes in dark blue, and Cryptophytes (outgroup) in gray. The numbers at the nodes indicate bootstrap values. Distinct groups of the Phragmoplastophyta were collapsed for clarity.

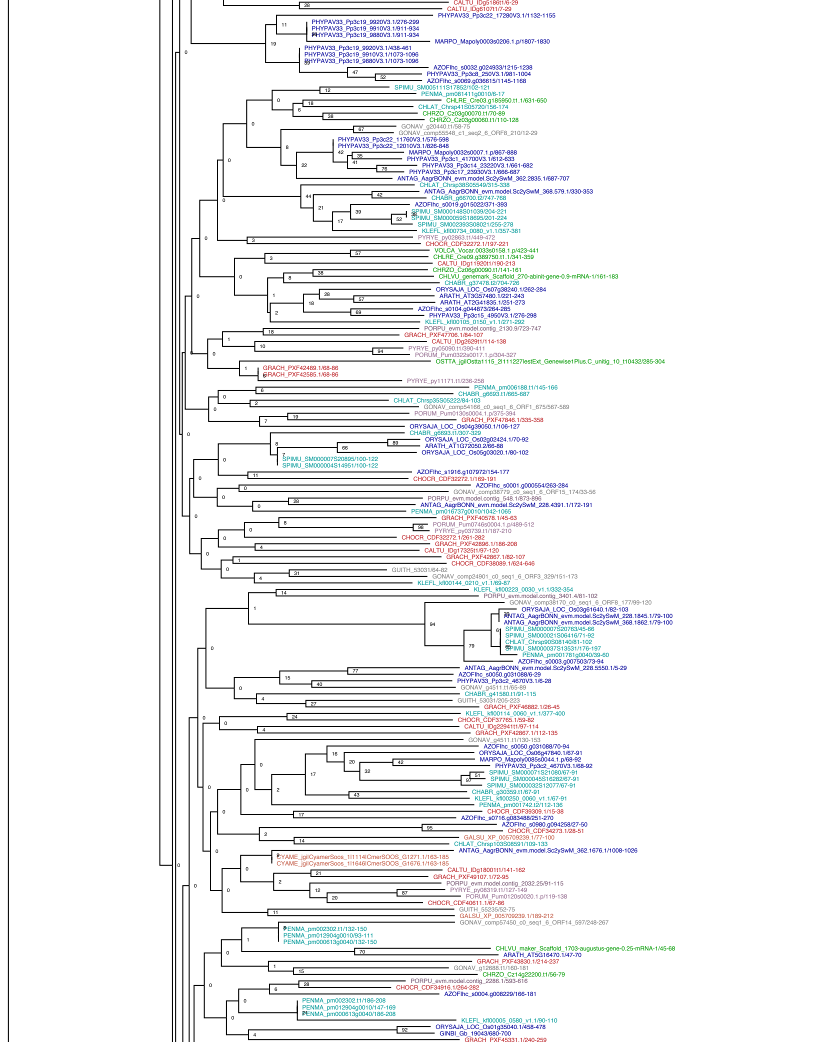
CALTU_IDg5186t1/6-29
CALTU_IDg6107t1/7-29
PHYPAV33_Pp3c22_17280V3.1/1132-1155
PHYPAV33_Pp3c19_9920V3.1/276-299
PHYPAV33_Pp3c19_9910V3.1/911-934
PHYPAV33_Pp3c19_9880V3.1/911-934
MARPO_Mapoly0003s0206.1.p/1807-1830
PHYPAV33_Pp3c19_9920V3.1/438-461
PHYPAV33_Pp3c19_9910V3.1/1073-1096
PHYPAV33_Pp3c19_9880V3.1/1073-1096
AZOFIhc_s0032.g024933/1215-1238
PHYPAV33_Pp3c8_250V3.1/981-1004
AZOFIhc_s0069.g036615/1145-1168
SPIMU_SM005111S17852/102-121
PENMA_pm081411g0010/6-17
CHLRE_Cre03.g185950.t1.1/631-650
CHLAT_Chrsp41S05720/156-174
CHRZO_Cz03g00070.t1/70-89
CHRZO_Cz03g00060.t1/110-128
GONAV_g20440.t1/58-75
GONAV_comp55548_c1_seq2_6_ORF8_210/12-29
PHYPAV33_Pp3c22_11760V3.1/576-598
PHYPAV33_Pp3c22_12010V3.1/826-848
MARPO_Mapoly0032s0007.1.p/867-888
PHYPAV33_Pp3c1_41700V3.1/612-633
PHYPAV33_Pp3c14_23220V3.1/661-682
PHYPAV33_Pp3c17_23930V3.1/666-687
ANTAG_AagrBONN_evm.model.Sc2ySwM_362.2835.1/687-707
CHLAT_Chrsp38S05549/315-338
ANTAG_AagrBONN_evm.model.Sc2ySwM_368.579.1/330-353
CHABR_g66700.t2/747-768
AZOFIhc_s0019.g015022/371-393
SPIMU_SM000148S01039/204-221
SPIMU_SM000059S18695/201-224
SPIMU_SM002393S08021/255-278
KLEFL_kfl00734_0080_v1.1/357-381
PYRYE_py02863.t1/449-472
CHOCR_CDF32272.1/197-221
VOLCA_Vocar.0033s0158.1.p/423-441
CHLRE_Cre09.g389750.t1.1/341-359
CALTU_IDg11920t1/190-213
CHRZO_Cz06g00090.t1/141-161
CHLVU_genemark_Scaffold_270-abinit-gene-0.9-mRNA-1/161-183
CHABR_g37478.t2/704-726
ORYSAJA_LOC_Os07g38240.1/262-284
ARATH_AT3G57480.1/221-243
ARATH_AT2G41835.1/251-273
AZOFIhc_s0104.g044873/264-285
PHYPAV33_Pp3c15_4950V3.1/276-298
KLEFL_kfl00105_0150_v1.1/271-292
PORPU_evm.model.contig_2130.9/723-747
GRACH_PXF47706.1/84-107
CALTU_IDg2629t1/114-138
PYRYE_py05090.t1/390-411
PORUM_Pum0322s0017.1.p/304-327
OSTTA_jgi|Ostta1115_2|111227|estExt_Genewise1Plus.C_unitig_10_t10432/285-304
GRACH_PXF42489.1/68-86
GRACH_PXF42585.1/68-86
PYRYE_py11171.t1/236-258
PENMA_pm006188.t1/145-166
CHABR_g6693.t1/665-687
CHLAT_Chrsp35S05222/84-103
GONAV_comp54166_c0_seq1_6_ORF1_675/567-589
PORUM_Pum0130s0004.1.p/375-394
GRACH_PXF47846.1/335-358
ORYSAJA_LOC_Os04g39050.1/106-127
CHABR_g6693.t1/307-329
ORYSAJA_LOC_Os02g02424.1/70-92
ARATH_AT1G72050.2/66-88
ORYSAJA_LOC_Os05g03020.1/80-102
SPIMU_SM000007S20895/100-122
SPIMU_SM000004S14951/100-122
AZOFIhc_s1916.g107972/154-177
CHOCR_CDF32272.1/169-191
AZOFIhc_s0001.g000554/263-284
GONAV_comp38779_c0_seq1_6_ORF15_174/33-56
PORPU_evm.model.contig_548.1/873-896
ANTAG_AagrBONN_evm.model.Sc2ySwM_228.4391.1/172-191
PENMA_pm016737g0010/1042-1065
GRACH_PXF40578.1/45-63
PORUM_Pum0746s0004.1.p/489-512
PYRYE_py03739.t1/187-210
CHOCR_CDF32272.1/261-282
GRACH_PXF42896.1/186-208
CALTU_IDg17325t1/97-120
GRACH_PXF42867.1/82-107
CHOCR_CDF38089.1/624-646
GUITH_53031/64-82
GONAV_comp24901_c0_seq1_6_ORF3_329/151-173
KLEFL_kfl00144_0210_v1.1/69-87
KLEFL_kfl00223_0030_v1.1/332-354
PORPU_evm.model.contig_3401.4/81-102
GONAV_comp38170_c0_seq1_6_ORF8_177/99-120
ORYSAJA_LOC_Os03g61640.1/82-103
ANTAG_AagrBONN_evm.model.Sc2ySwM_228.1845.1/79-100
ANTAG_AagrBONN_evm.model.Sc2ySwM_368.1862.1/79-100
SPIMU_SM000007S20763/45-66
SPIMU_SM000021S06416/71-92
CHLAT_Chrsp90S08140/81-102
SPIMU_SM000037S13531/176-197
PENMA_pm001781g0040/39-60
AZOFIhc_s0003.g007503/73-94
ANTAG_AagrBONN_evm.model.Sc2ySwM_228.5550.1/5-29
AZOFIhc_s0050.g031088/6-29
PHYPAV33_Pp3c2_4670V3.1/6-28
GONAV_g4511.t1/65-89
CHABR_g41580.t1/91-115
GUITH_53031/205-223
GRACH_PXF46882.1/26-45
KLEFL_kfl00114_0060_v1.1/377-400
CHOCR_CDF37765.1/59-82
CALTU_IDg22941t1/97-114
GRACH_PXF42867.1/112-135
GONAV_g4511.t1/130-153
AZOFIhc_s0050.g031088/70-94
ORYSAJA_LOC_Os06g47840.1/67-91
MARPO_Mapoly0085s0044.1.p/68-92
PHYPAV33_Pp3c2_4670V3.1/68-92
SPIMU_SM000071S21080/67-91
SPIMU_SM000045S16282/67-91
SPIMU_SM000032S12077/67-91
CHABR_g30359.t1/67-91
KLEFL_kfl00250_0060_v1.1/67-91
PENMA_pm001742.t2/112-136
CHOCR_CDF39309.1/15-38
AZOFIhc_s0716.g083488/251-270
AZOFIhc_s0980.g094258/27-50
CHOCR_CDF34273.1/28-51
GALSU_XP_005709239.1/77-100
CHLAT_Chrsp103S08591/109-133
ANTAG_AagrBONN_evm.model.Sc2ySwM_362.1676.1/1008-1026
CYAME_jgi|CyamerSoos_1|1114|CmerSOOS_G1271.1/163-185
CYAME_jgi|CyamerSoos_1|1646|CmerSOOS_G1676.1/163-185
CALTU_IDg18001t1/141-162
GRACH_PXF49107.1/72-95
PORPU_evm.model.contig_2032.25/91-115
PYRYE_py08319.t1/127-149
PORUM_Pum0120s0020.1.p/119-138
CHOCR_CDF40611.1/67-86
GUITH_55235/52-75
GALSU_XP_005709239.1/189-212
GONAV_comp57450_c0_seq1_6_ORF14_597/248-267
PENMA_pm002302.t1/132-150
PENMA_pm012904g0010/93-111
PENMA_pm000613g0040/132-150
CHLVU_maker_Scaffold_1703-augustus-gene-0.25-mRNA-1/45-68
ARATH_AT5G16470.1/47-70
GRACH_PXF43830.1/214-237
GONAV_g12688.t1/160-181
CHRZO_Cz14g22200.t1/56-79
PORPU_evm.model.contig_2286.1/593-616
CHOCR_CDF34916.1/264-282
AZOFIhc_s0004.g008229/166-181
PENMA_pm002302.t1/186-208
PENMA_pm012904g0010/147-169
PENMA_pm000613g0040/186-208
KLEFL_kfl00005_0580_v1.1/90-110
ORYSAJA_LOC_Os01g35040.1/458-478
GINBI_Gb_19043/680-700
GRACH_PXF45331.1/240-259

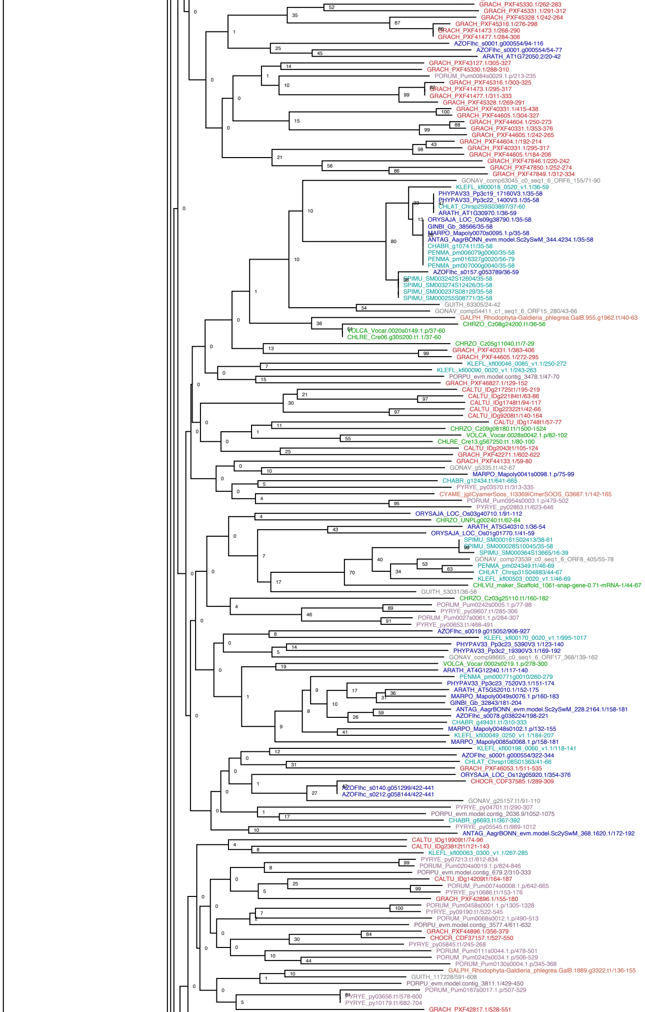
GRACH_PXF45330.1/262-283
GRACH_PXF45331.1/291-312
GRACH_PXF45328.1/242-264
GRACH_PXF45316.1/276-298
GRACH_PXF41473.1/268-290
GRACH_PXF41477.1/284-306
AZOFIhc_s0001.g000554/94-116
AZOFIhc_s0001.g000554/54-77
ARATH_AT1G72050.2/20-42
GRACH_PXF43127.1/305-327
GRACH_PXF45330.1/288-310
PORUM_Pum0084s0029.1.p/213-235
GRACH_PXF45316.1/303-325
GRACH_PXF41473.1/295-317
GRACH_PXF41477.1/311-333
GRACH_PXF45328.1/269-291
GRACH_PXF40331.1/415-438
GRACH_PXF44605.1/304-327
GRACH_PXF44604.1/250-273
GRACH_PXF40331.1/353-376
GRACH_PXF44605.1/242-265
GRACH_PXF44604.1/192-214
GRACH_PXF40331.1/295-317
GRACH_PXF44605.1/184-206
GRACH_PXF47846.1/220-242
GRACH_PXF47850.1/252-274
GRACH_PXF47849.1/312-334
GONAV_comp63045_c0_seq1_6_ORF6_155/71-90
KLEFL_kfl00018_0520_v1.1/36-59
PHYPAV33_Pp3c19_17160V3.1/35-58
PHYPAV33_Pp3c22_1400V3.1/35-58
CHLAT_Chrsp259S03897/37-60
ARATH_AT1G30970.1/36-59
ORYSAJA_LOC_Os09g38790.1/35-58
GINBI_Gb_38566/35-58
MARPO_Mapoly0070s0095.1.p/35-58
ANTAG_AagrBONN_evm.model.Sc2ySwM_344.4234.1/35-58
CHABR_g1074.t1/35-58
PENMA_pm006079g0060/35-58
PENMA_pm016327g0020/56-79
PENMA_pm007000g0040/35-58
AZOFIhc_s0157.g053789/36-59
SPIMU_SM003242S12604/35-58
SPIMU_SM003274S12426/35-58
SPIMU_SM000237S08129/35-58
SPIMU_SM000255S08771/35-58
GUITH_63305/24-42
GONAV_comp54411_c1_seq1_6_ORF15_280/43-66
GALPH_Rhodophyta-Galdieria_phlegrea.GalB.955.g1962.t1/40-63
CHRZO_Cz08g24200.t1/36-56
VOLCA_Vocar.0020s0149.1.p/37-60
CHLRE_Cre06.g305200.t1.1/37-60
CHRZO_Cz05g11040.t1/7-29
GRACH_PXF40331.1/383-406
GRACH_PXF44605.1/272-295
KLEFL_kfl00046_0085_v1.1/250-272
KLEFL_kfl00090_0020_v1.1/243-263
PORPU_evm.model.contig_3478.1/47-70
GRACH_PXF46827.1/129-152
CALTU_IDg21725t1/195-219
CALTU_IDg22184t1/63-86
CALTU_IDg1748t1/94-117
CALTU_IDg22322t1/42-66
CALTU_IDg9208t1/140-164
CALTU_IDg1748t1/57-77
CHRZO_Cz09g08180.t1/1500-1524
VOLCA_Vocar.0028s0042.1.p/82-102
CHLRE_Cre13.g567250.t1.1/80-100
CALTU_IDg2043t1/105-124
GRACH_PXF42271.1/602-622
GRACH_PXF44133.1/59-80
GONAV_g5335.t1/42-67
MARPO_Mapoly0041s0098.1.p/75-99
CHABR_g12434.t1/641-665
PYRYE_py03570.t1/313-335
CYAME_jgi|CyamerSoos_1|3369|CmerSOOS_G3687.1/142-165
PORUM_Pum0954s0003.1.p/479-502
PYRYE_py02863.t1/623-646
ORYSAJA_LOC_Os03g40710.1/91-112
CHRZO_UNPLg00240.t1/62-84
ARATH_AT5G40310.1/36-54
ORYSAJA_LOC_Os01g01770.1/41-59
SPIMU_SM000161S02413/38-61
SPIMU_SM000028S10045/35-58
SPIMU_SM000364S13665/16-39
GONAV_comp73539_c0_seq1_6_ORF8_405/55-78
PENMA_pm024349.t1/46-69
CHLAT_Chrsp31S04883/44-67
KLEFL_kfl00503_0020_v1.1/46-69
CHLVU_maker_Scaffold_1061-snap-gene-0.71-mRNA-1/44-67
GUITH_53031/36-58
CHRZO_Cz03g25110.t1/160-182
PORUM_Pum0242s0005.1.p/77-98
PYRYE_py09607.t1/285-306
PORUM_Pum0027s0061.1.p/284-307
PYRYE_py00653.t1/468-491
AZOFIhc_s0019.g015052/906-927
KLEFL_kfl00170_0020_v1.1/995-1017
PHYPAV33_Pp3c23_5390V3.1/123-140
PHYPAV33_Pp3c2_19390V3.1/169-192
GONAV_comp98665_c0_seq1_6_ORF17_368/139-162
VOLCA_Vocar.0002s0219.1.p/278-300
ARATH_AT4G12240.1/117-140
PENMA_pm000771g0010/260-279
PHYPAV33_Pp3c23_7520V3.1/151-174
ARATH_AT5G52010.1/152-175
MARPO_Mapoly0049s0076.1.p/160-183
GINBI_Gb_32843/181-204
ANTAG_AagrBONN_evm.model.Sc2ySwM_228.2164.1/158-181
AZOFIhc_s0078.g038224/198-221
CHABR_g49431.t1/310-333
MARPO_Mapoly0048s0102.1.p/132-155
KLEFL_kfl00049_0250_v1.1/184-207
MARPO_Mapoly0085s0068.1.p/158-181
KLEFL_kfl00198_0060_v1.1/118-141
AZOFIhc_s0001.g000554/322-344
CHLAT_Chrsp108S01363/41-66
GRACH_PXF46053.1/511-535
ORYSAJA_LOC_Os12g05920.1/354-376
CHOCR_CDF37585.1/289-309
AZOFIhc_s0140.g051299/422-441
AZOFIhc_s0212.g058144/422-441
GONAV_g25157.t1/91-110
PYRYE_py04701.t1/290-307
PORPU_evm.model.contig_2036.9/1052-1075
CHABR_g6693.t1/367-392
PYRYE_py05545.t1/989-1012
ANTAG_AagrBONN_evm.model.Sc2ySwM_368.1620.1/172-192
CALTU_IDg19909t1/74-96
CALTU_IDg23812t1/121-143
KLEFL_kfl00063_0300_v1.1/267-285
PYRYE_py07213.t1/812-834
PORUM_Pum0204s0019.1.p/824-846
PORPU_evm.model.contig_679.2/310-333
CALTU_IDg14209t1/164-187
PORUM_Pum0074s0008.1.p/642-665
PYRYE_py10686.t1/153-176
GRACH_PXF42896.1/155-180
PORUM_Pum0458s0001.1.p/1305-1328
PYRYE_py09190.t1/522-545
PORUM_Pum0068s0012.1.p/490-513
PORPU_evm.model.contig_3577.4/611-632
GRACH_PXF44896.1/356-379
CHOCR_CDF37157.1/527-550
PYRYE_py05845.t1/245-268
PORUM_Pum0111s0044.1.p/478-501
PORUM_Pum0242s0034.1.p/506-529
PORUM_Pum0130s0004.1.p/345-368
GALPH_Rhodophyta-Galdieria_phlegrea.GalB.1889.g3322.t1/136-155
GUITH_117228/591-608
PORPU_evm.model.contig_3811.1/429-450
PORUM_Pum0167s0017.1.p/507-529
PYRYE_py03656.t1/578-600
PYRYE_py10179.t1/682-704
GRACH_PXF42817.1/528-551

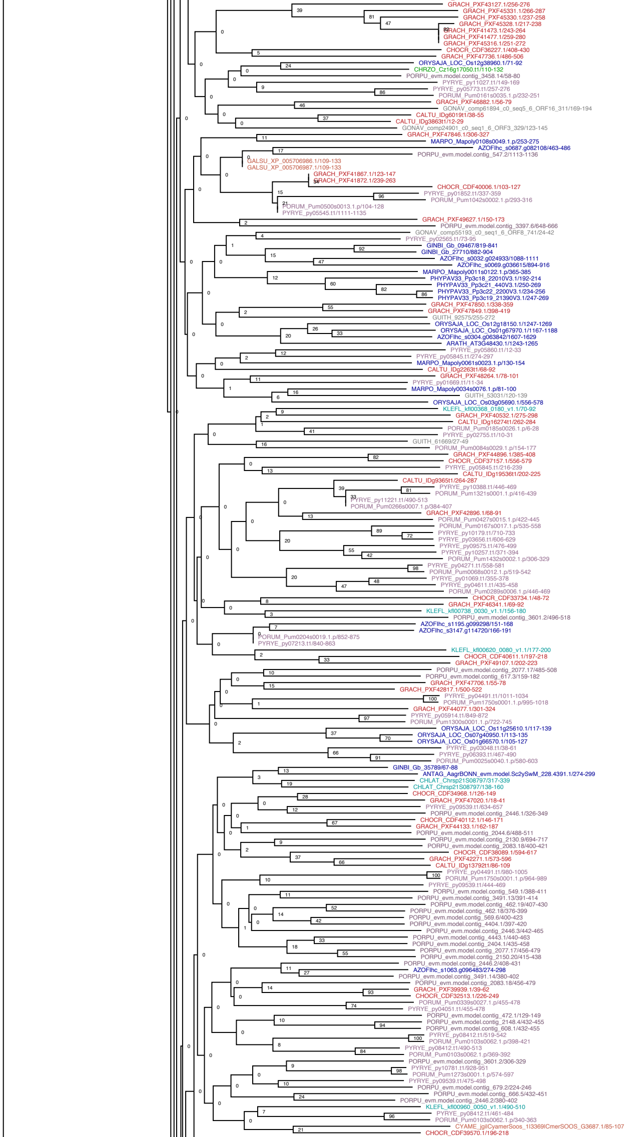
GRACH_PXF43127.1/256-276
GRACH_PXF45331.1/266-287
GRACH_PXF45330.1/237-258
GRACH_PXF45328.1/217-238
GRACH_PXF41473.1/243-264
GRACH_PXF41477.1/259-280
GRACH_PXF45316.1/251-272
CHOCR_CDF36227.1/408-430
GRACH_PXF47736.1/486-506
ORYSAJA_LOC_Os12g38960.1/71-92
CHRZO_Cz16g17050.t1/110-132
PORPU_evm.model.contig_3458.14/58-80
PYRYE_py11027.t1/149-169
PYRYE_py05773.t1/257-276
PORUM_Pum0161s0035.1.p/232-251
GRACH_PXF46882.1/56-79
GONAV_comp61894_c0_seq5_6_ORF16_311/169-194
CALTU_IDg6019t1/38-55
CALTU_IDg3863t1/12-29
GONAV_comp24901_c0_seq1_6_ORF3_329/123-145
GRACH_PXF47846.1/306-327
MARPO_Mapoly0108s0049.1.p/253-275
AZOFIhc_s0687.g082108/463-486
PORPU_evm.model.contig_547.2/1113-1136
GALSU_XP_005706986.1/109-133
GALSU_XP_005706987.1/109-133
GRACH_PXF41867.1/123-147
GRACH_PXF41872.1/239-263
CHOCR_CDF40006.1/103-127
PYRYE_py01852.t1/337-359
PORUM_Pum1042s0002.1.p/293-316
PORUM_Pum0500s0013.1.p/104-128
PYRYE_py05545.t1/1111-1135
GRACH_PXF49627.1/150-173
PORPU_evm.model.contig_3397.6/648-666
GONAV_comp55193_c0_seq1_6_ORF8_741/24-42
PYRYE_py02565.t1/73-95
GINBI_Gb_09467/819-841
GINBI_Gb_27710/882-904
AZOFIhc_s0032.g024933/1088-1111
AZOFIhc_s0069.g036615/894-916
MARPO_Mapoly0011s0122.1.p/365-385
PHYPAV33_Pp3c18_22010V3.1/192-214
PHYPAV33_Pp3c21_440V3.1/250-269
PHYPAV33_Pp3c22_2200V3.1/234-256
PHYPAV33_Pp3c19_21390V3.1/247-269
GRACH_PXF47850.1/338-359
GRACH_PXF47849.1/398-419
GUITH_92575/255-272
ORYSAJA_LOC_Os12g18150.1/1247-1269
ORYSAJA_LOC_Os01g67970.1/1167-1188
AZOFIhc_s0304.g063842/1607-1629
ARATH_AT3G48430.1/1243-1265
PYRYE_py05860.t1/12-33
PYRYE_py05845.t1/274-297
MARPO_Mapoly0061s0023.1.p/130-154
CALTU_IDg2263t1/68-92
GRACH_PXF48264.1/78-101
PYRYE_py01669.t1/11-34
MARPO_Mapoly0034s0076.1.p/81-100
GUITH_53031/120-139
ORYSAJA_LOC_Os03g05690.1/556-578
KLEFL_kfl00368_0180_v1.1/70-92
GRACH_PXF40532.1/275-298
CALTU_IDg16274t1/262-284
PORUM_Pum0185s0026.1.p/6-28
PYRYE_py02755.t1/10-31
GUITH_61669/27-49
PORUM_Pum0084s0029.1.p/154-177
GRACH_PXF44896.1/385-408
CHOCR_CDF37157.1/556-579
PYRYE_py05845.t1/216-239
CALTU_IDg19536t1/202-225
CALTU_IDg9365t1/264-287
PYRYE_py10388.t1/446-469
PORUM_Pum1321s0001.1.p/416-439
PYRYE_py11221.t1/490-513
PORUM_Pum0266s0007.1.p/384-407
GRACH_PXF42896.1/68-91
PORUM_Pum0427s0015.1.p/422-445
PORUM_Pum0167s0017.1.p/535-558
PYRYE_py10179.t1/710-733
PYRYE_py03656.t1/606-629
PYRYE_py09575.t1/476-499
PYRYE_py10257.t1/371-394
PORUM_Pum1432s0002.1.p/306-329
PYRYE_py04271.t1/558-581
PORUM_Pum0068s0012.1.p/519-542
PYRYE_py01069.t1/355-378
PYRYE_py04611.t1/435-458
PORUM_Pum0289s0006.1.p/446-469
CHOCR_CDF33734.1/48-72
GRACH_PXF46341.1/69-92
KLEFL_kfl00738_0030_v1.1/156-180
PORPU_evm.model.contig_3601.2/496-518
AZOFIhc_s1195.g099298/151-168
AZOFIhc_s3147.g114720/166-191
PORUM_Pum0204s0019.1.p/852-875
PYRYE_py07213.t1/840-863
KLEFL_kfl00620_0080_v1.1/177-200
CHOCR_CDF40611.1/197-218
GRACH_PXF49107.1/202-223
PORPU_evm.model.contig_2077.17/485-508
PORPU_evm.model.contig_617.3/159-182
GRACH_PXF47706.1/55-78
GRACH_PXF42817.1/500-522
PYRYE_py04491.t1/1011-1034
PORUM_Pum1750s0001.1.p/995-1018
GRACH_PXF44077.1/301-324
PYRYE_py05914.t1/849-872
PORUM_Pum1300s0001.1.p/722-745
ORYSAJA_LOC_Os11g25610.1/117-139
ORYSAJA_LOC_Os07g40950.1/113-135
ORYSAJA_LOC_Os01g66570.1/105-127
PYRYE_py03048.t1/38-61
PYRYE_py06393.t1/467-490
PORUM_Pum0025s0040.1.p/580-603
GINBI_Gb_35789/67-88
ANTAG_AagrBONN_evm.model.Sc2ySwM_228.4391.1/274-299
CHLAT_Chrsp21S08797/317-339
CHLAT_Chrsp21S08797/138-160
CHOCR_CDF34968.1/126-149
GRACH_PXF47020.1/18-41
PYRYE_py09539.t1/634-657
PORPU_evm.model.contig_2446.1/326-349
CHOCR_CDF40112.1/146-171
GRACH_PXF44133.1/162-187
PORPU_evm.model.contig_2044.6/488-511
PORPU_evm.model.contig_2130.9/694-717
PORPU_evm.model.contig_2083.18/400-421
CHOCR_CDF38089.1/594-617
GRACH_PXF42271.1/573-596
CALTU_IDg13792t1/86-109
PYRYE_py04491.t1/980-1005
PORUM_Pum1750s0001.1.p/964-989
PYRYE_py09539.t1/444-469
PORPU_evm.model.contig_549.1/388-411
PORPU_evm.model.contig_3491.13/391-414
PORPU_evm.model.contig_462.19/407-430
PORPU_evm.model.contig_462.18/376-399
PORPU_evm.model.contig_569.6/400-423
PORPU_evm.model.contig_4404.1/397-420
PORPU_evm.model.contig_2446.3/442-465
PORPU_evm.model.contig_4443.1/440-463
PORPU_evm.model.contig_2404.1/435-458
PORPU_evm.model.contig_2077.17/456-479
PORPU_evm.model.contig_2150.20/415-438
PORPU_evm.model.contig_2446.2/408-431
AZOFIhc_s1063.g096483/274-298
PORPU_evm.model.contig_3491.14/380-402
PORPU_evm.model.contig_2083.18/456-479
GRACH_PXF39939.1/39-62
CHOCR_CDF32513.1/226-249
PORUM_Pum0339s0027.1.p/455-478
PYRYE_py04051.t1/455-478
PORPU_evm.model.contig_472.1/129-149
PORPU_evm.model.contig_2148.4/432-455
PORPU_evm.model.contig_608.1/432-455
PYRYE_py08412.t1/519-542
PORUM_Pum0103s0062.1.p/398-421
PYRYE_py08412.t1/490-513
PORUM_Pum0103s0062.1.p/369-392
PORPU_evm.model.contig_3601.2/306-329
PYRYE_py10781.t1/928-951
PORUM_Pum1273s0001.1.p/574-597
PYRYE_py09539.t1/475-498
PORPU_evm.model.contig_679.2/224-246
PORPU_evm.model.contig_666.5/432-451
PORPU_evm.model.contig_2446.2/380-402
KLEFL_kfl00960_0050_v1.1/490-510
PYRYE_py08412.t1/461-484
PORUM_Pum0103s0062.1.p/340-363
CYAME_jgi|CyamerSoos_1|3369|CmerSOOS_G3687.1/85-107
CHOCR_CDF39570.1/196-218

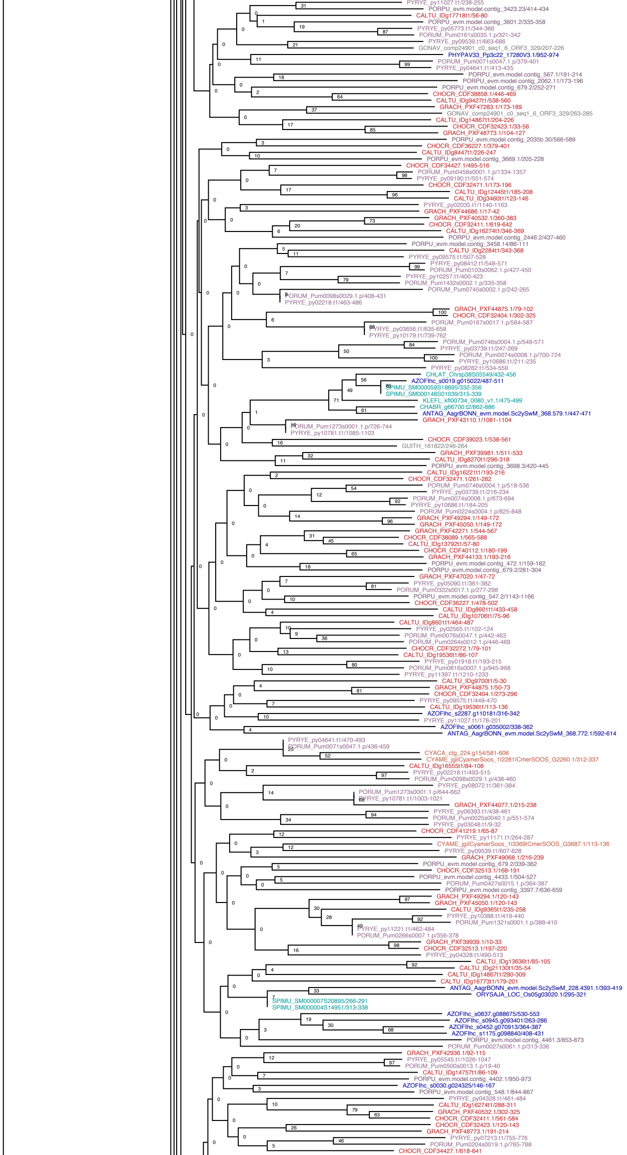
PYRYE_py11027.t1/238-255
PORPU_evm.model.contig_3423.23/414-434
CALTU_IDg17718t1/56-80
PORPU_evm.model.contig_3601.2/335-358
PYRYE_py05773.t1/344-366
PORUM_Pum0161s0035.1.p/321-342
PYRYE_py09539.t1/663-686
GONAV_comp24901_c0_seq1_6_ORF3_329/207-226
PHYPAV33_Pp3c22_17280V3.1/952-974
PORUM_Pum0071s0047.1.p/379-401
PYRYE_py04641.t1/413-435
PORPU_evm.model.contig_567.1/191-214
PORPU_evm.model.contig_2062.11/173-196
PORPU_evm.model.contig_679.2/252-271
CHOCR_CDF38858.1/446-469
CALTU_IDg9427t1/538-560
GRACH_PXF47283.1/173-189
GONAV_comp24901_c0_seq1_6_ORF3_329/263-285
CALTU_IDg14867t1/204-226
CHOCR_CDF32423.1/33-56
GRACH_PXF48773.1/104-127
PORPU_evm.model.contig_2035b.30/566-589
CHOCR_CDF36227.1/379-401
CALTU_IDg8447t1/226-247
PORPU_evm.model.contig_3669.1/205-228
CHOCR_CDF34427.1/495-516
PORUM_Pum0458s0001.1.p/1334-1357
PYRYE_py09190.t1/551-574
CHOCR_CDF32471.1/173-196
CALTU_IDg12445t1/185-208
CALTU_IDg3460t1/123-146
PYRYE_py02035.t1/1140-1163
GRACH_PXF44686.1/17-42
GRACH_PXF40532.1/360-383
CHOCR_CDF32411.1/619-642
CALTU_IDg16274t1/346-369
PORPU_evm.model.contig_2446.2/437-460
PORPU_evm.model.contig_3458.14/86-111
CALTU_IDg2284t1/343-368
PYRYE_py09575.t1/507-528
PYRYE_py08412.t1/548-571
PORUM_Pum0103s0062.1.p/427-450
PYRYE_py10257.t1/400-423
PORUM_Pum1432s0002.1.p/335-358
PORUM_Pum0745s0002.1.p/242-265
PORUM_Pum0098s0029.1.p/408-431
PYRYE_py02218.t1/463-486
GRACH_PXF44875.1/79-102
CHOCR_CDF32404.1/302-325
PORUM_Pum0167s0017.1.p/564-587
PYRYE_py03656.t1/635-658
PYRYE_py10179.t1/739-762
PORUM_Pum0746s0004.1.p/549-571
PYRYE_py03739.t1/247-269
PORUM_Pum0074s0008.1.p/700-724
PYRYE_py10686.t1/211-235
PYRYE_py08282.t1/534-559
CHLAT_Chrsp38S05549/432-456
AZOFIhc_s0019.g015022/487-511
SPIMU_SM000059S18695/332-356
SPIMU_SM000148S01039/315-339
KLEFL_kfl00734_0080_v1.1/475-499
CHABR_g66700.t2/862-886
ANTAG_AagrBONN_evm.model.Sc2ySwM_368.579.1/447-471
GRACH_PXF43110.1/1081-1104
PORUM_Pum1273s0001.1.p/726-744
PYRYE_py10781.t1/1085-1103
CHOCR_CDF39023.1/538-561
GUITH_161822/246-264
GRACH_PXF39981.1/511-533
CALTU_IDg8270t1/296-318
PORPU_evm.model.contig_3698.3/420-445
CALTU_IDg16221t1/193-216
CHOCR_CDF32471.1/261-282
PORUM_Pum0746s0004.1.p/518-536
PYRYE_py03739.t1/216-234
PORUM_Pum0074s0008.1.p/673-694
PYRYE_py10686.t1/184-205
PORUM_Pum0224s0004.1.p/825-848
GRACH_PXF49294.1/149-172
GRACH_PXF45050.1/149-172
GRACH_PXF42271.1/544-567
CHOCR_CDF38089.1/565-588
CALTU_IDg13792t1/57-80
CHOCR_CDF40112.1/180-199
GRACH_PXF44133.1/193-216
PORPU_evm.model.contig_472.1/159-182
PORPU_evm.model.contig_679.2/281-304
GRACH_PXF47020.1/47-72
PYRYE_py05090.t1/361-382
PORUM_Pum0322s0017.1.p/277-298
PORPU_evm.model.contig_547.2/1143-1166
CHOCR_CDF36227.1/478-502
CALTU_IDg8601t1/433-458
CALTU_IDg10706t1/75-96
CALTU_IDg8601t1/464-487
PYRYE_py02565.t1/102-124
PORUM_Pum0076s0047.1.p/442-463
PORUM_Pum0264s0012.1.p/446-469
CHOCR_CDF32272.1/79-101
CALTU_IDg19536t1/86-107
PYRYE_py01918.t1/193-215
PORUM_Pum0816s0007.1.p/945-968
PYRYE_py11397.t1/1210-1233
CALTU_IDg9700t1/5-30
GRACH_PXF44875.1/50-73
CHOCR_CDF32404.1/273-296
PYRYE_py09575.t1/449-470
CALTU_IDg19536t1/113-136
AZOFIhc_s2287.g110181/316-342
PYRYE_py11027.t1/178-201
AZOFIhc_s0061.g035002/338-362
ANTAG_AagrBONN_evm.model.Sc2ySwM_368.772.1/592-614
PYRYE_py04641.t1/470-493
PORUM_Pum0071s0047.1.p/436-459
CYACA_ctg_224.g154/581-606
CYAME_jgi|CyamerSoos_1|2281|CmerSOOS_G2260.1/312-337
CALTU_IDg16555t1/84-108
PYRYE_py02218.t1/493-515
PORUM_Pum0098s0029.1.p/438-460
PYRYE_py08072.t1/361-384
PORUM_Pum1273s0001.1.p/644-662
PYRYE_py10781.t1/1003-1021
GRACH_PXF44077.1/215-238
PYRYE_py06393.t1/438-461
PORUM_Pum0025s0040.1.p/551-574
PYRYE_py03048.t1/9-32
CHOCR_CDF41219.1/65-87
PYRYE_py11171.t1/264-287
CYAME_jgi|CyamerSoos_1|3369|CmerSOOS_G3687.1/113-136
PYRYE_py09539.t1/607-628
GRACH_PXF49068.1/216-239
PORPU_evm.model.contig_679.2/339-362
CHOCR_CDF32513.1/168-191
PORPU_evm.model.contig_4433.1/504-527
PORUM_Pum0427s0015.1.p/364-387
PORPU_evm.model.contig_3397.7/636-659
GRACH_PXF49294.1/120-143
GRACH_PXF45050.1/120-143
CALTU_IDg9365t1/235-258
PYRYE_py10388.t1/419-440
PORUM_Pum1321s0001.1.p/388-410
PYRYE_py11221.t1/462-484
PORUM_Pum0266s0007.1.p/356-378
GRACH_PXF39939.1/10-33
CHOCR_CDF32513.1/197-220
PYRYE_py04328.t1/490-513
CALTU_IDg13636t1/85-105
CALTU_IDg21130t1/35-54
CALTU_IDg14867t1/290-309
CALTU_IDg16773t1/179-201
ANTAG_AagrBONN_evm.model.Sc2ySwM_228.4391.1/393-419
ORYSAJA_LOC_Os05g03020.1/295-321
SPIMU_SM000007S20895/266-291
SPIMU_SM000004S14951/313-338
AZOFIhc_s0837.g088675/530-553
AZOFIhc_s0945.g093401/263-286
AZOFIhc_s0452.g070913/364-387
AZOFIhc_s1175.g098840/408-431
PORPU_evm.model.contig_4461.3/853-873
PORUM_Pum0027s0061.1.p/313-336
GRACH_PXF42936.1/92-115
PYRYE_py05545.t1/1026-1047
PORUM_Pum0500s0013.1.p/19-40
CALTU_IDg14757t1/86-109
PORPU_evm.model.contig_4402.1/950-973
AZOFIhc_s0030.g024325/146-167
PORPU_evm.model.contig_548.1/844-867
PYRYE_py04328.t1/461-484
CALTU_IDg16274t1/288-311
GRACH_PXF40532.1/302-325
CHOCR_CDF32411.1/561-584
CHOCR_CDF32423.1/120-143
GRACH_PXF48773.1/191-214
PYRYE_py07213.t1/755-776
PORUM_Pum0204s0019.1.p/765-788
CHOCR_CDF34427.1/618-641

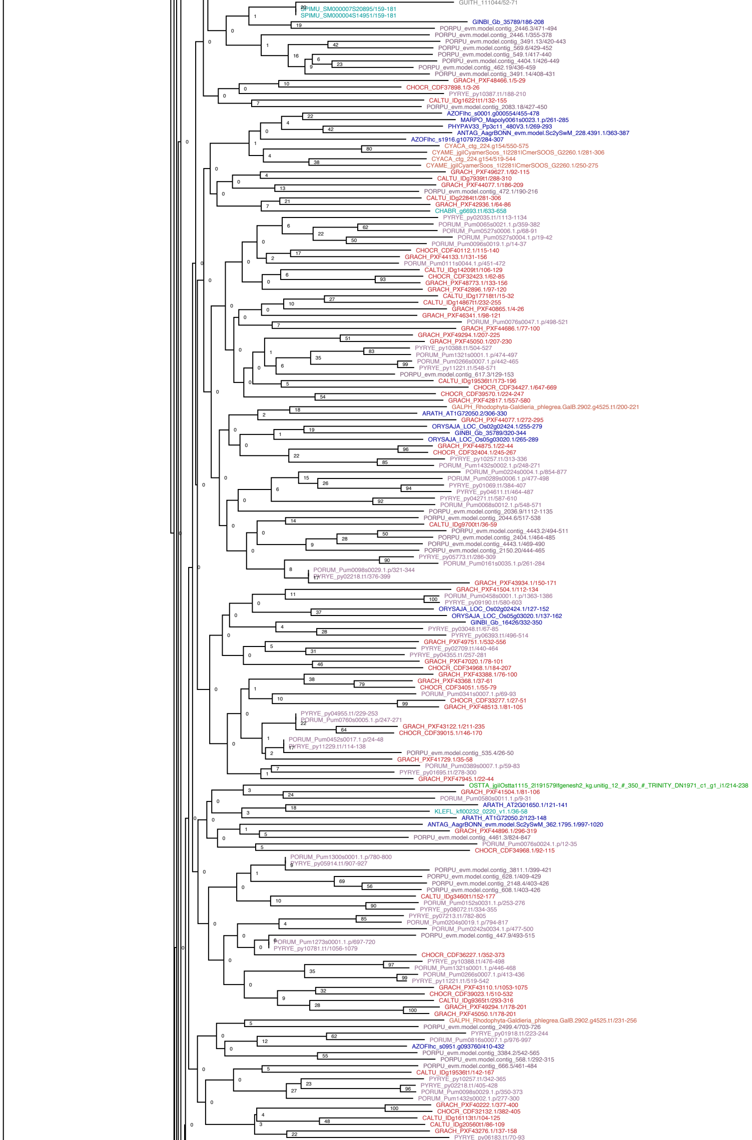
GUITH_111044/52-71
SPIMU_SM000007S20895/159-181
SPIMU_SM000004S14951/159-181
GINBI_Gb_35789/186-208
PORPU_evm.model.contig_2446.3/471-494
PORPU_evm.model.contig_2446.1/355-378
PORPU_evm.model.contig_3491.13/420-443
PORPU_evm.model.contig_569.6/429-452
PORPU_evm.model.contig_549.1/417-440
PORPU_evm.model.contig_4404.1/426-449
PORPU_evm.model.contig_462.19/436-459
PORPU_evm.model.contig_3491.14/408-431
GRACH_PXF48466.1/5-29
CHOCR_CDF37898.1/3-26
PYRYE_py10387.t1/188-210
CALTU_IDg16221t1/132-155
PORPU_evm.model.contig_2083.18/427-450
AZOFIhc_s0001.g000554/455-478
MARPO_Mapoly0061s0023.1.p/261-285
PHYPAV33_Pp3c11_480V3.1/269-293
ANTAG_AagrBONN_evm.model.Sc2ySwM_228.4391.1/363-387
AZOFIhc_s1916.g107972/284-307
CYACA_ctg_224.g154/550-575
CYAME_jgilCyamerSoos_1l2281lCmerSOOS_G2260.1/281-306
CYACA_ctg_224.g154/519-544
CYAME_jgilCyamerSoos_1l2281lCmerSOOS_G2260.1/250-275
GRACH_PXF49627.1/92-115
CALTU_IDg7939t1/288-310
GRACH_PXF44077.1/186-209
PORPU_evm.model.contig_472.1/190-216
CALTU_IDg2284t1/281-306
GRACH_PXF42936.1/64-86
CHABR_g6693.t1/633-658
PYRYE_py02035.t1/1113-1134
PORUM_Pum0065s0021.1.p/359-382
PORUM_Pum0527s0006.1.p/68-91
PORUM_Pum0527s0004.1.p/19-42
PORUM_Pum0096s0019.1.p/14-37
CHOCR_CDF40112.1/115-140
GRACH_PXF44133.1/131-156
PORUM_Pum0111s0044.1.p/451-472
CALTU_IDg14209t1/106-129
CHOCR_CDF32423.1/62-85
GRACH_PXF48773.1/133-156
GRACH_PXF42896.1/97-120
CALTU_IDg17718t1/15-32
CALTU_IDg14867t1/232-255
GRACH_PXF40865.1/4-26
GRACH_PXF46341.1/98-121
PORUM_Pum0076s0047.1.p/498-521
GRACH_PXF44686.1/77-100
GRACH_PXF49294.1/207-225
GRACH_PXF45050.1/207-230
PYRYE_py10388.t1/504-527
PORUM_Pum1321s0001.1.p/474-497
PORUM_Pum0266s0007.1.p/442-465
PYRYE_py11221.t1/548-571
PORPU_evm.model.contig_617.3/129-153
CALTU_IDg19536t1/173-196
CHOCR_CDF34427.1/647-669
CHOCR_CDF39570.1/224-247
GRACH_PXF42817.1/557-580
GALPH_Rhodophyta-Galdieria_phlegrea.GalB.2902.g4525.t1/200-221
ARATH_AT1G72050.2/306-330
GRACH_PXF44077.1/272-295
ORYSAJA_LOC_Os02g02424.1/255-279
GINBI_Gb_35789/320-344
ORYSAJA_LOC_Os05g03020.1/265-289
GRACH_PXF44875.1/22-44
CHOCR_CDF32404.1/245-267
PYRYE_py10257.t1/313-336
PORUM_Pum1432s0002.1.p/248-271
PORUM_Pum0224s0004.1.p/854-877
PORUM_Pum0289s0006.1.p/477-498
PYRYE_py01069.t1/384-407
PYRYE_py04611.t1/464-487
PYRYE_py04271.t1/587-610
PORUM_Pum0068s0012.1.p/548-571
PORPU_evm.model.contig_2036.9/1112-1135
PORPU_evm.model.contig_2044.6/517-538
CALTU_IDg9700t1/36-59
PORPU_evm.model.contig_4443.2/494-511
PORPU_evm.model.contig_2404.1/464-485
PORPU_evm.model.contig_4443.1/469-490
PORPU_evm.model.contig_2150.20/444-465
PYRYE_py05773.t1/286-309
PORUM_Pum0161s0035.1.p/261-284
PORUM_Pum0098s0029.1.p/321-344
PYRYE_py02218.t1/376-399
GRACH_PXF43934.1/150-171
GRACH_PXF41504.1/112-134
PORUM_Pum0458s0001.1.p/1363-1386
PYRYE_py09190.t1/580-603
ORYSAJA_LOC_Os02g02424.1/127-152
ORYSAJA_LOC_Os05g03020.1/137-162
GINBI_Gb_16426/332-350
PYRYE_py03048.t1/67-85
PYRYE_py06393.t1/496-514
GRACH_PXF49751.1/532-556
PYRYE_py02709.t1/440-464
PYRYE_py04355.t1/257-281
GRACH_PXF47020.1/78-101
CHOCR_CDF34968.1/184-207
GRACH_PXF43388.1/76-100
GRACH_PXF43368.1/37-61
CHOCR_CDF34051.1/55-79
PORUM_Pum0341s0007.1.p/69-93
CHOCR_CDF33277.1/27-51
GRACH_PXF48513.1/81-105
PYRYE_py04955.t1/229-253
PORUM_Pum0760s0005.1.p/247-271
GRACH_PXF43122.1/211-235
CHOCR_CDF39015.1/146-170
PORUM_Pum0452s0017.1.p/24-48
PYRYE_py11229.t1/114-138
PORPU_evm.model.contig_535.4/26-50
GRACH_PXF41729.1/35-58
PORUM_Pum0389s0007.1.p/59-83
PYRYE_py01695.t1/278-300
GRACH_PXF47945.1/22-44
OSTTA_jgilOstta1115_2l191579lfgenesh2_kg.unitig_12_#_350_#_TRINITY_DN1971_c1_g1_i1/214-238
GRACH_PXF41504.1/81-106
PORUM_Pum0580s0011.1.p/9-31
ARATH_AT2G01650.1/121-141
KLEFL_kfl00232_0220_v1.1/36-58
ARATH_AT1G72050.2/123-148
ANTAG_AagrBONN_evm.model.Sc2ySwM_362.1795.1/997-1020
GRACH_PXF44896.1/296-319
PORPU_evm.model.contig_4461.3/824-847
PORUM_Pum0076s0024.1.p/12-35
CHOCR_CDF34968.1/92-115
PORUM_Pum1300s0001.1.p/780-800
PYRYE_py05914.t1/907-927
PORPU_evm.model.contig_3811.1/399-421
PORPU_evm.model.contig_628.1/409-429
PORPU_evm.model.contig_2148.4/403-426
PORPU_evm.model.contig_608.1/403-426
CALTU_IDg3460t1/152-177
PORUM_Pum0152s0031.1.p/253-276
PYRYE_py08072.t1/334-355
PYRYE_py07213.t1/782-805
PORUM_Pum0204s0019.1.p/794-817
PORUM_Pum0242s0034.1.p/477-500
PORPU_evm.model.contig_447.9/493-515
PORUM_Pum1273s0001.1.p/697-720
PYRYE_py10781.t1/1056-1079
CHOCR_CDF36227.1/352-373
PYRYE_py10388.t1/476-498
PORUM_Pum1321s0001.1.p/446-468
PORUM_Pum0266s0007.1.p/413-436
PYRYE_py11221.t1/519-542
GRACH_PXF43110.1/1053-1075
CHOCR_CDF39023.1/510-532
CALTU_IDg9365t1/293-316
GRACH_PXF49294.1/178-201
GRACH_PXF45050.1/178-201
GALPH_Rhodophyta-Galdieria_phlegrea.GalB.2902.g4525.t1/231-256
PORPU_evm.model.contig_2499.4/703-726
PYRYE_py01918.t1/223-244
PORUM_Pum0816s0007.1.p/976-997
AZOFIhc_s0951.g093760/410-432
PORPU_evm.model.contig_3384.2/542-565
PORPU_evm.model.contig_568.1/292-315
PORPU_evm.model.contig_666.5/461-484
CALTU_IDg19536t1/142-167
PYRYE_py10257.t1/342-365
PYRYE_py02218.t1/405-428
PORUM_Pum0098s0029.1.p/350-373
PORUM_Pum1432s0002.1.p/277-300
GRACH_PXF40222.1/377-400
CHOCR_CDF32132.1/382-405
CALTU_IDg16113t1/104-125
CALTU_IDg20560t1/86-109
GRACH_PXF43276.1/137-158
PYRYE_py06183.t1/70-93

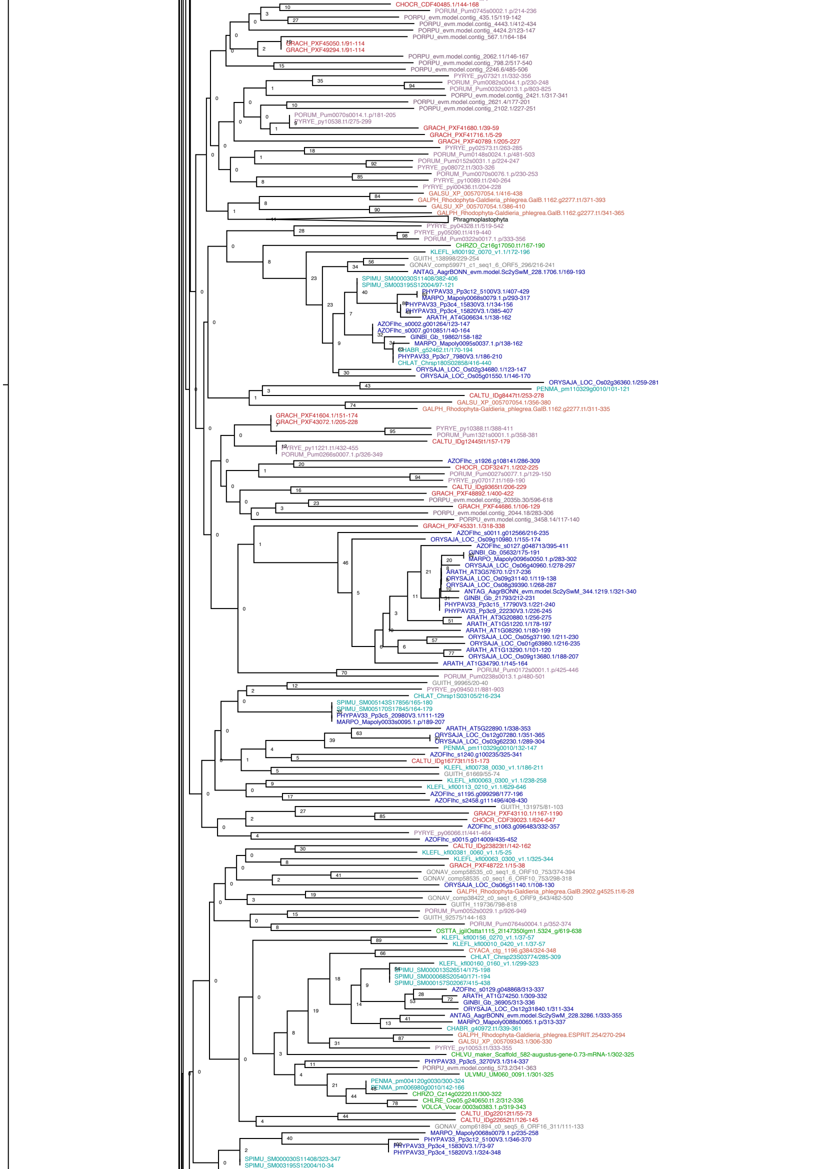
CHOCR_CDF40485.1/144-168
PORUM_Pum0745s0002.1.p/214-236
PORPU_evm.model.contig_435.15/119-142
PORPU_evm.model.contig_4443.1/412-434
PORPU_evm.model.contig_4424.2/123-147
PORPU_evm.model.contig_567.1/164-184
GRACH_PXF45050.1/91-114
GRACH_PXF49294.1/91-114
PORPU_evm.model.contig_2062.11/146-167
PORPU_evm.model.contig_798.2/517-540
PORPU_evm.model.contig_2246.6/485-506
PYRYE_py07321.t1/332-356
PORUM_Pum0082s0044.1.p/230-248
PORUM_Pum0032s0013.1.p/803-825
PORPU_evm.model.contig_2421.1/317-341
PORPU_evm.model.contig_2621.4/177-201
PORPU_evm.model.contig_2102.1/227-251
PORUM_Pum0070s0014.1.p/181-205
PYRYE_py10538.t1/275-299
GRACH_PXF41680.1/39-59
GRACH_PXF41716.1/5-29
GRACH_PXF40789.1/205-227
PYRYE_py02573.t1/263-285
PORUM_Pum0148s0024.1.p/481-503
PORUM_Pum0152s0031.1.p/224-247
PYRYE_py08072.t1/303-326
PORUM_Pum0070s0076.1.p/230-253
PYRYE_py10089.t1/240-264
PYRYE_pyi00436.t1/204-228
GALSU_XP_005707054.1/416-438
GALPH_Rhodophyta-Galdieria_phlegrea.GalB.1162.g2277.t1/371-393
GALSU_XP_005707054.1/386-410
GALPH_Rhodophyta-Galdieria_phlegrea.GalB.1162.g2277.t1/341-365
Phragmoplastophyta
PYRYE_py04328.t1/519-542
PYRYE_py05090.t1/419-440
PORUM_Pum0322s0017.1.p/333-356
CHRZO_Cz16g17050.t1/167-190
KLEFL_kfl00192_0070_v1.1/172-196
GUITH_138998/229-254
GONAV_comp59971_c1_seq1_6_ORF5_296/216-241
ANTAG_AagrBONN_evm.model.Sc2ySwM_228.1706.1/169-193
SPIMU_SM000030S11408/382-406
SPIMU_SM003195S12004/97-121
PHYPAV33_Pp3c12_5100V3.1/407-429
MARPO_Mapoly0068s0079.1.p/293-317
PHYPAV33_Pp3c4_15830V3.1/134-156
PHYPAV33_Pp3c4_15820V3.1/385-407
ARATH_AT4G06634.1/138-162
AZOFIhc_s0002.g001264/123-147
AZOFIhc_s0007.g010851/140-164
GINBI_Gb_19862/158-182
MARPO_Mapoly0095s0037.1.p/138-162
CHABR_g52462.t1/170-194
PHYPAV33_Pp3c7_7980V3.1/186-210
CHLAT_Chrsp180S02858/416-440
ORYSAJA_LOC_Os02g34680.1/123-147
ORYSAJA_LOC_Os05g01550.1/146-170
ORYSAJA_LOC_Os02g36360.1/259-281
PENMA_pm110329g0010/101-121
CALTU_IDg8447t1/253-278
GALSU_XP_005707054.1/356-380
GALPH_Rhodophyta-Galdieria_phlegrea.GalB.1162.g2277.t1/311-335
GRACH_PXF41604.1/151-174
GRACH_PXF43072.1/205-228
PYRYE_py10388.t1/388-411
PORUM_Pum1321s0001.1.p/358-381
CALTU_IDg12445t1/157-179
PYRYE_py11221.t1/432-455
PORUM_Pum0266s0007.1.p/326-349
AZOFIhc_s1926.g108141/286-309
CHOCR_CDF32471.1/202-225
PORUM_Pum0027s0077.1.p/129-150
PYRYE_py07017.t1/169-190
CALTU_IDg9365t1/206-229
GRACH_PXF48892.1/400-422
PORPU_evm.model.contig_2035b.30/596-618
GRACH_PXF44686.1/106-129
PORPU_evm.model.contig_2044.18/283-306
PORPU_evm.model.contig_3458.14/117-140
GRACH_PXF45331.1/318-338
AZOFIhc_s0011.g012566/216-235
ORYSAJA_LOC_Os09g10980.1/155-174
AZOFIhc_s0127.g048713/395-411
GINBI_Gb_05632/175-191
MARPO_Mapoly0096s0050.1.p/283-302
ORYSAJA_LOC_Os06g40960.1/278-297
ARATH_AT3G57670.1/217-236
ORYSAJA_LOC_Os09g31140.1/119-138
ORYSAJA_LOC_Os08g39390.1/268-287
ANTAG_AagrBONN_evm.model.Sc2ySwM_344.1219.1/321-340
GINBI_Gb_21793/212-231
PHYPAV33_Pp3c15_17790V3.1/221-240
PHYPAV33_Pp3c9_22230V3.1/226-245
ARATH_AT3G20880.1/256-275
ARATH_AT1G51220.1/178-197
ARATH_AT1G08290.1/180-199
ORYSAJA_LOC_Os05g37190.1/211-230
ORYSAJA_LOC_Os01g63980.1/216-235
ARATH_AT1G13290.1/101-120
ORYSAJA_LOC_Os09g13680.1/188-207
ARATH_AT1G34790.1/145-164
PORUM_Pum0172s0001.1.p/425-446
PORUM_Pum0238s0013.1.p/480-501
GUITH_99965/20-40
PYRYE_py09450.t1/881-903
CHLAT_Chrsp1S03105/216-234
SPIMU_SM005143S17856/165-180
SPIMU_SM005170S17845/164-179
PHYPAV33_Pp3c5_20980V3.1/111-129
MARPO_Mapoly0033s0095.1.p/189-207
ARATH_AT5G22890.1/338-353
ORYSAJA_LOC_Os12g07280.1/351-365
ORYSAJA_LOC_Os03g62230.1/289-304
PENMA_pm110329g0010/132-147
AZOFIhc_s1240.g100235/325-341
CALTU_IDg16773t1/151-173
KLEFL_kfl00738_0030_v1.1/186-211
GUITH_61669/55-74
KLEFL_kfl00063_0300_v1.1/238-258
KLEFL_kfl00113_0210_v1.1/629-646
AZOFIhc_s1195.g099298/177-196
AZOFIhc_s2458.g111496/408-430
GUITH_131975/81-103
GRACH_PXF43110.1/1167-1190
CHOCR_CDF39023.1/624-647
AZOFIhc_s1063.g096483/332-357
PYRYE_py06066.t1/441-464
AZOFIhc_s0015.g014009/435-452
CALTU_IDg23823t1/142-162
KLEFL_kfl00381_0060_v1.1/5-25
KLEFL_kfl00063_0300_v1.1/325-344
GRACH_PXF48722.1/15-38
GONAV_comp58535_c0_seq1_6_ORF10_753/374-394
GONAV_comp58535_c0_seq1_6_ORF10_753/298-318
ORYSAJA_LOC_Os06g51140.1/108-130
GALPH_Rhodophyta-Galdieria_phlegrea.GalB.2902.g4525.t1/6-28
GONAV_comp38422_c0_seq1_6_ORF9_643/482-500
GUITH_119736/798-818
PORUM_Pum0052s0029.1.p/926-949
GUITH_92575/144-163
PORUM_Pum0764s0004.1.p/352-374
OSTTA_jgi|Ostta1115_2|147350|gm1.5324_g/619-638
KLEFL_kfl00156_0270_v1.1/37-57
KLEFL_kfl00010_0420_v1.1/37-57
CYACA_ctg_1196.g384/324-348
CHLAT_Chrsp23S03774/285-309
KLEFL_kfl00160_0160_v1.1/299-323
SPIMU_SM000013S26514/175-198
SPIMU_SM000068S20540/171-194
SPIMU_SM000157S02067/415-438
AZOFIhc_s0129.g048868/313-337
ARATH_AT1G74250.1/309-332
GINBI_Gb_36905/313-336
ORYSAJA_LOC_Os12g31840.1/311-334
ANTAG_AagrBONN_evm.model.Sc2ySwM_228.3286.1/333-355
MARPO_Mapoly0088s0065.1.p/313-337
CHABR_g40972.t1/339-361
GALPH_Rhodophyta-Galdieria_phlegrea.ESPRIT.254/270-294
GALSU_XP_005709343.1/306-330
PYRYE_py10053.t1/333-355
CHLVU_maker_Scaffold_582-augustus-gene-0.73-mRNA-1/302-325
PHYPAV33_Pp3c5_3270V3.1/314-337
PORPU_evm.model.contig_573.2/341-363
ULVMU_UM060_0091.1/301-325
PENMA_pm004120g0030/300-324
PENMA_pm006980g0010/142-166
CHRZO_Cz14g02220.t1/300-322
CHLRE_Cre05.g240650.t1.2/312-336
VOLCA_Vocar.0003s0383.1.p/319-343
CALTU_IDg22012t1/55-73
CALTU_IDg22652t1/126-145
GONAV_comp61894_c0_seq5_6_ORF16_311/111-133
MARPO_Mapoly0068s0079.1.p/235-258
PHYPAV33_Pp3c12_5100V3.1/346-370
PHYPAV33_Pp3c4_15830V3.1/73-97
PHYPAV33_Pp3c4_15820V3.1/324-348
SPIMU_SM000030S11408/323-347
SPIMU_SM003195S12004/10-34

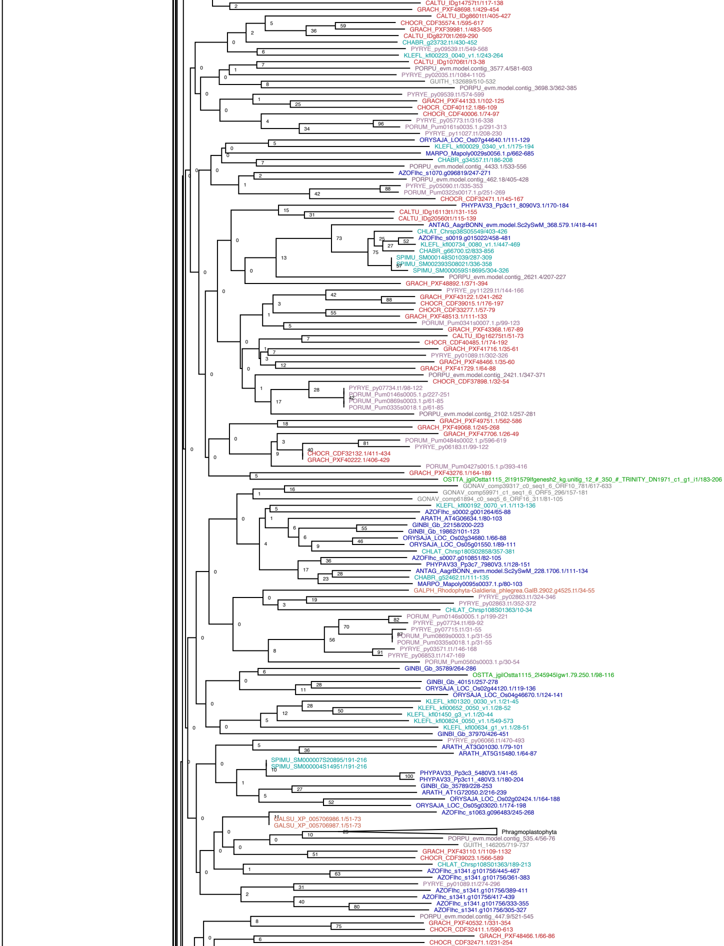
CALTU_IDg14757t1/117-138
GRACH_PXF48698.1/429-454
CALTU_IDg8601t1/405-427
CHOCR_CDF35574.1/595-617
GRACH_PXF39981.1/483-505
CALTU_IDg8270t1/269-290
CHABR_g23732.t1/430-452
PYRYE_py09539.t1/549-568
KLEFL_kfl00223_0040_v1.1/243-264
CALTU_IDg10706t1/13-38
PORPU_evm.model.contig_3577.4/581-603
PYRYE_py02035.t1/1084-1105
GUITH_132689/510-532
PORPU_evm.model.contig_3698.3/362-385
PYRYE_py09539.t1/574-599
GRACH_PXF44133.1/102-125
CHOCR_CDF40112.1/86-109
CHOCR_CDF40006.1/74-97
PYRYE_py05773.t1/316-338
PORUM_Pum0161s0035.1.p/291-313
PYRYE_py11027.t1/208-230
ORYSAJA_LOC_Os07g44640.1/111-129
KLEFL_kfl00029_0340_v1.1/175-194
MARPO_Mapoly0029s0056.1.p/662-685
CHABR_g34557.t1/186-208
PORPU_evm.model.contig_4433.1/533-556
AZOFlhc_s1070.g096819/247-271
PORPU_evm.model.contig_462.18/405-428
PYRYE_py05090.t1/335-353
PORUM_Pum0322s0017.1.p/251-269
CHOCR_CDF32471.1/145-167
PHYPAV33_Pp3c11_8090V3.1/170-184
CALTU_IDg16113t1/131-155
CALTU_IDg20560t1/115-139
ANTAG_AagrBONN_evm.model.Sc2ySwM_368.579.1/418-441
CHLAT_Chrsp38S05549/403-426
AZOFlhc_s0019.g015022/458-481
KLEFL_kfl00734_0080_v1.1/447-469
CHABR_g66700.t2/833-856
SPIMU_SM000148S01039/287-309
SPIMU_SM002393S08021/336-358
SPIMU_SM000059S18695/304-326
PORPU_evm.model.contig_2621.4/207-227
GRACH_PXF48892.1/371-394
PYRYE_py11229.t1/144-166
GRACH_PXF43122.1/241-262
CHOCR_CDF39015.1/176-197
CHOCR_CDF33277.1/57-79
GRACH_PXF48513.1/111-133
PORUM_Pum0341s0007.1.p/99-123
GRACH_PXF43368.1/67-89
CALTU_IDg16275t1/51-73
CHOCR_CDF40485.1/174-192
GRACH_PXF41716.1/35-61
PYRYE_py01089.t1/302-326
GRACH_PXF48466.1/35-60
GRACH_PXF41729.1/64-88
PORPU_evm.model.contig_2421.1/347-371
CHOCR_CDF37898.1/32-54
PYRYE_py07734.t1/98-122
PORUM_Pum0146s0005.1.p/227-251
PORUM_Pum0869s0003.1.p/61-85
PORUM_Pum0335s0018.1.p/61-85
PORPU_evm.model.contig_2102.1/257-281
GRACH_PXF49751.1/562-586
GRACH_PXF49068.1/245-268
GRACH_PXF47706.1/26-49
PORUM_Pum0484s0002.1.p/596-619
PYRYE_py06183.t1/99-122
CHOCR_CDF32132.1/411-434
GRACH_PXF40222.1/406-429
PORUM_Pum0427s0015.1.p/393-416
GRACH_PXF43276.1/164-189
OSTTA_jgi|Ostta1115_2|191579|fgenesh2_kg.unitig_12_#_350_#_TRINITY_DN1971_c1_g1_i1/183-206
GONAV_comp39317_c0_seq1_6_ORF10_781/617-633
GONAV_comp59971_c1_seq1_6_ORF5_296/157-181
GONAV_comp61894_c0_seq5_6_ORF16_311/81-105
KLEFL_kfl00192_0070_v1.1/113-136
AZOFlhc_s0002.g001264/65-88
ARATH_AT4G06634.1/80-103
GINBI_Gb_22158/200-223
GINBI_Gb_19862/101-123
ORYSAJA_LOC_Os02g34680.1/66-88
ORYSAJA_LOC_Os05g01550.1/89-111
CHLAT_Chrsp180S02858/357-381
AZOFlhc_s0007.g010851/82-105
PHYPAV33_Pp3c7_7980V3.1/128-151
ANTAG_AagrBONN_evm.model.Sc2ySwM_228.1706.1/111-134
CHABR_g52462.t1/111-135
MARPO_Mapoly0095s0037.1.p/80-103
GALPH_Rhodophyta-Galdieria_phlegrea.GalB.2902.g4525.t1/34-55
PYRYE_py02863.t1/324-346
PYRYE_py02863.t1/352-372
CHLAT_Chrsp108S01363/10-34
PORUM_Pum0146s0005.1.p/199-221
PYRYE_py07734.t1/69-92
PYRYE_py07715.t1/31-55
PORUM_Pum0869s0003.1.p/31-55
PORUM_Pum0335s0018.1.p/31-55
PYRYE_py03571.t1/146-168
PYRYE_py06853.t1/147-169
PORUM_Pum0560s0003.1.p/30-54
GINBI_Gb_35789/264-286
OSTTA_jgi|Ostta1115_2|45945|gw1.79.250.1/98-116
GINBI_Gb_40151/257-278
ORYSAJA_LOC_Os02g44120.1/119-136
ORYSAJA_LOC_Os04g46670.1/124-141
KLEFL_kfl01320_0030_v1.1/21-45
KLEFL_kfl00652_0050_v1.1/28-52
KLEFL_kfl01450_g3_v1.1/20-44
KLEFL_kfl00824_0050_v1.1/549-573
KLEFL_kfl00634_g1_v1.1/28-51
GINBI_Gb_37970/426-451
PYRYE_py06066.t1/470-493
ARATH_AT3G01030.1/79-101
ARATH_AT5G15480.1/64-87
SPIMU_SM000007S20895/191-216
SPIMU_SM000004S14951/191-216
PHYPAV33_Pp3c3_5480V3.1/41-65
PHYPAV33_Pp3c11_480V3.1/180-204
GINBI_Gb_35789/228-253
ARATH_AT1G72050.2/216-239
ORYSAJA_LOC_Os02g02424.1/164-188
ORYSAJA_LOC_Os05g03020.1/174-198
AZOFlhc_s1063.g096483/245-268
GALSU_XP_005706986.1/51-73
GALSU_XP_005706987.1/51-73
Phragmoplastophyta
PORPU_evm.model.contig_535.4/56-76
GUITH_146205/719-737
GRACH_PXF43110.1/1109-1132
CHOCR_CDF39023.1/566-589
CHLAT_Chrsp108S01363/189-213
AZOFlhc_s1341.g101756/445-467
AZOFlhc_s1341.g101756/361-383
PYRYE_py01089.t1/274-296
AZOFlhc_s1341.g101756/389-411
AZOFlhc_s1341.g101756/417-439
AZOFlhc_s1341.g101756/333-355
AZOFlhc_s1341.g101756/305-327
PORPU_evm.model.contig_447.9/521-545
GRACH_PXF40532.1/331-354
CHOCR_CDF32411.1/590-613
GRACH_PXF48466.1/66-86
CHOCR_CDF32471.1/231-254

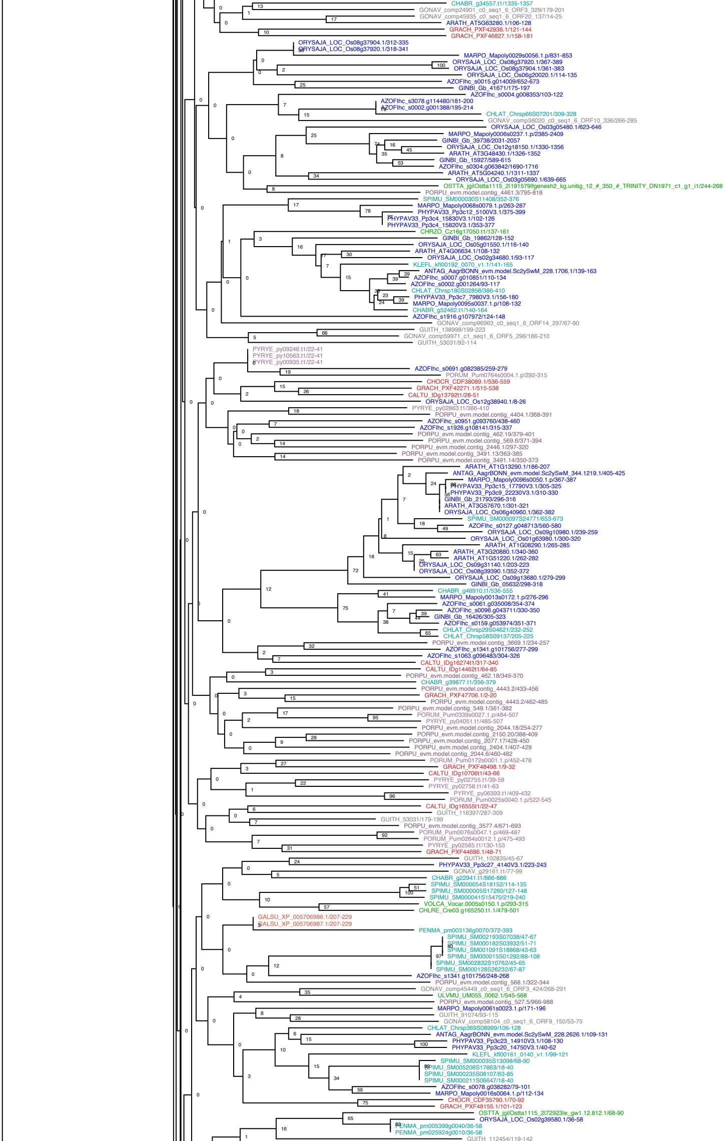
CHABR_g34557.t1/1335-1357
GONAV_comp24901_c0_seq1_6_ORF3_329/179-201
GONAV_comp45935_c0_seq1_6_ORF20_137/14-25
ARATH_AT5G63280.1/106-128
GRACH_PXF42936.1/121-144
GRACH_PXF46827.1/158-181
ORYSAJA_LOC_Os08g37904.1/312-335
ORYSAJA_LOC_Os08g37920.1/318-341
MARPO_Mapoly0029s0056.1.p/831-853
ORYSAJA_LOC_Os08g37920.1/367-389
ORYSAJA_LOC_Os08g37904.1/361-383
ORYSAJA_LOC_Os06g20020.1/114-135
AZOFIhc_s0015.g014009/652-673
GINBI_Gb_41671/175-197
AZOFIhc_s0004.g008353/103-122
AZOFIhc_s3078.g114480/181-200
AZOFIhc_s0002.g001388/195-214
CHLAT_Chrsp66S07201/309-328
GONAV_comp38020_c0_seq1_6_ORF10_336/266-285
ORYSAJA_LOC_Os03g05480.1/623-646
MARPO_Mapoly0006s0237.1.p/2385-2409
GINBI_Gb_39738/2031-2057
ORYSAJA_LOC_Os12g18150.1/1330-1356
ARATH_AT3G48430.1/1326-1352
GINBI_Gb_15927/589-615
AZOFIhc_s0304.g063842/1690-1716
ARATH_AT5G04240.1/1311-1337
ORYSAJA_LOC_Os03g05690.1/639-665
OSTTA_jgi|Ostta1115_2|191579|fgenesh2_kg.unitig_12_#_350_#_TRINITY_DN1971_c1_g1_i1/244-268
PORPU_evm.model.contig_4461.3/795-818
SPIMU_SM000030S11408/352-376
MARPO_Mapoly0068s0079.1.p/263-287
PHYPAV33_Pp3c12_5100V3.1/375-399
PHYPAV33_Pp3c4_15830V3.1/102-126
PHYPAV33_Pp3c4_15820V3.1/353-377
CHRZO_Cz16g17050.t1/137-161
GINBI_Gb_19862/128-152
ORYSAJA_LOC_Os05g01550.1/116-140
ARATH_AT4G06634.1/108-132
ORYSAJA_LOC_Os02g34680.1/93-117
KLEFL_kfl00192_0070_v1.1/141-165
ANTAG_AagrBONN_evm.model.Sc2ySwM_228.1706.1/139-163
AZOFIhc_s0007.g010851/110-134
AZOFIhc_s0002.g001264/93-117
CHLAT_Chrsp180S02858/386-410
PHYPAV33_Pp3c7_7980V3.1/156-180
MARPO_Mapoly0095s0037.1.p/108-132
CHABR_g52462.t1/140-164
AZOFIhc_s1916.g107972/124-148
GONAV_comp96963_c0_seq1_6_ORF14_297/67-90
GUITH_138998/199-223
GONAV_comp59971_c1_seq1_6_ORF5_296/186-210
GUITH_53031/92-114
PYRYE_py09246.t1/22-41
PYRYE_py10563.t1/22-41
PYRYE_py00935.t1/22-41
AZOFIhc_s0691.g082385/259-279
PORUM_Pum0764s0004.1.p/292-315
CHOCR_CDF38089.1/536-559
GRACH_PXF42271.1/515-538
CALTU_IDg13792t1/28-51
ORYSAJA_LOC_Os12g38940.1/8-26
PYRYE_py02863.t1/386-410
PORPU_evm.model.contig_4404.1/368-391
AZOFIhc_s0951.g093760/438-460
AZOFIhc_s1926.g108141/315-337
PORPU_evm.model.contig_462.19/379-401
PORPU_evm.model.contig_569.6/371-394
PORPU_evm.model.contig_2446.1/297-320
PORPU_evm.model.contig_3491.13/363-385
PORPU_evm.model.contig_3491.14/350-373
ARATH_AT1G13290.1/186-207
ANTAG_AagrBONN_evm.model.Sc2ySwM_344.1219.1/405-425
MARPO_Mapoly0096s0050.1.p/367-387
PHYPAV33_Pp3c15_17790V3.1/305-325
PHYPAV33_Pp3c9_22230V3.1/310-330
GINBI_Gb_21793/296-316
ARATH_AT3G57670.1/301-321
ORYSAJA_LOC_Os06g40960.1/362-382
SPIMU_SM000097S24771/653-673
AZOFIhc_s0127.g048713/560-580
ORYSAJA_LOC_Os09g10980.1/239-259
ORYSAJA_LOC_Os01g63980.1/300-320
ARATH_AT1G08290.1/265-285
ARATH_AT3G20880.1/340-360
ARATH_AT1G51220.1/262-282
ORYSAJA_LOC_Os09g31140.1/203-223
ORYSAJA_LOC_Os08g39390.1/352-372
ORYSAJA_LOC_Os09g13680.1/279-299
GINBI_Gb_05632/298-318
CHABR_g46910.t1/536-555
MARPO_Mapoly0013s0172.1.p/276-296
AZOFIhc_s0061.g035008/354-374
AZOFIhc_s0096.g043711/330-350
GINBI_Gb_16426/305-323
AZOFIhc_s0159.g053974/351-371
CHLAT_Chrsp29S04621/232-252
CHLAT_Chrsp58S09137/205-225
PORPU_evm.model.contig_3669.1/234-257
AZOFIhc_s1341.g101756/277-299
AZOFIhc_s1063.g096483/304-326
CALTU_IDg16274t1/317-340
CALTU_IDg14462t1/64-85
PORPU_evm.model.contig_462.18/349-370
CHABR_g39877.t1/356-379
PORPU_evm.model.contig_4443.2/433-456
GRACH_PXF47706.1/2-20
PORPU_evm.model.contig_4443.2/462-485
PORPU_evm.model.contig_549.1/361-382
PORUM_Pum0339s0027.1.p/484-507
PYRYE_py04051.t1/485-507
PORPU_evm.model.contig_2044.18/254-277
PORPU_evm.model.contig_2150.20/388-409
PORPU_evm.model.contig_2077.17/428-450
PORPU_evm.model.contig_2404.1/407-429
PORPU_evm.model.contig_2044.6/460-482
PORUM_Pum0172s0001.1.p/452-478
GRACH_PXF48498.1/9-32
CALTU_IDg10706t1/43-66
PYRYE_py02755.t1/39-59
PYRYE_py02758.t1/41-63
PYRYE_py06393.t1/409-432
PORUM_Pum0025s0040.1.p/522-545
CALTU_IDg16555t1/22-47
GUITH_116397/287-309
GUITH_53031/179-199
PORPU_evm.model.contig_3577.4/671-693
PORUM_Pum0076s0047.1.p/469-487
PORUM_Pum0264s0012.1.p/475-493
PYRYE_py02565.t1/130-153
GRACH_PXF44686.1/48-71
GUITH_102835/45-67
PHYPAV33_Pp3c27_4140V3.1/223-243
GONAV_g29161.t1/77-99
CHABR_g22941.t1/866-886
SPIMU_SM000054S18152/114-135
SPIMU_SM000005S17260/127-148
SPIMU_SM000041S15470/219-240
VOLCA_Vocar.0005s0150.1.p/293-315
CHLRE_Cre03.g165250.t1.1/479-501
GALSU_XP_005706986.1/207-229
GALSU_XP_005706987.1/207-229
PENMA_pm003136g0070/372-393
SPIMU_SM002193S07038/47-67
SPIMU_SM000182S03932/51-71
SPIMU_SM001091S18868/43-63
SPIMU_SM000015S01292/88-108
SPIMU_SM002832S10762/45-65
SPIMU_SM000128S26232/67-87
AZOFIhc_s1341.g101756/248-268
PORPU_evm.model.contig_568.1/322-344
GONAV_comp45449_c0_seq1_6_ORF3_424/268-291
ULVMU_UM055_0062.1/545-568
PORPU_evm.model.contig_527.5/966-988
MARPO_Mapoly0061s0023.1.p/171-196
GUITH_91074/93-115
GONAV_comp58104_c0_seq1_6_ORF9_150/53-75
CHLAT_Chrsp369S08999/106-128
ANTAG_AagrBONN_evm.model.Sc2ySwM_228.2626.1/109-131
PHYPAV33_Pp3c23_14910V3.1/108-130
PHYPAV33_Pp3c20_14750V3.1/40-62
KLEFL_kfl00161_0140_v1.1/99-121
SPIMU_SM000035S13098/68-90
SPIMU_SM005208S17863/18-40
SPIMU_SM000235S08107/63-85
SPIMU_SM000211S06647/18-40
AZOFIhc_s0078.g038282/79-101
MARPO_Mapoly0016s0064.1.p/112-134
CHOCR_CDF35790.1/70-92
GRACH_PXF48155.1/101-123
OSTTA_jgi|Ostta1115_2|72923|e_gw1.12.812.1/68-90
ORYSAJA_LOC_Os02g39580.1/36-58
PENMA_pm005399g0040/36-58
PENMA_pm025924g0010/36-58
GUITH_112454/119-142

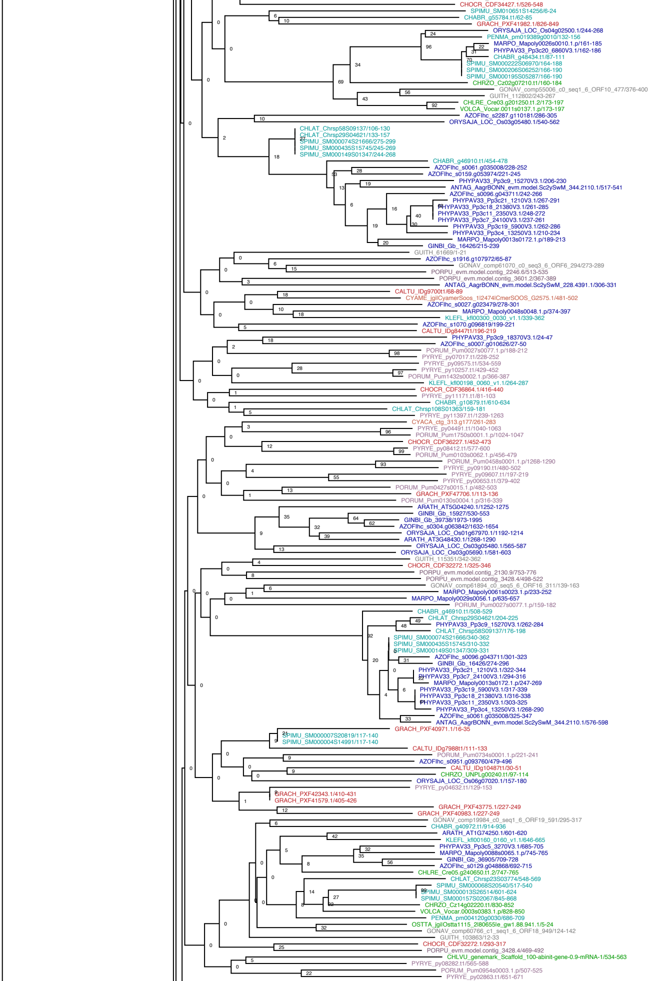

CHOCR_CDF34427.1/526-548
SPIMU_SM010651S14256/6-24
CHABR_g55784.t1/62-85
GRACH_PXF41982.1/826-849
ORYSAJA_LOC_Os04g02500.1/244-268
PENMA_pm019389g0010/132-156
MARPO_Mapoly0026s0010.1.p/161-185
PHYPAV33_Pp3c20_6860V3.1/162-186
CHABR_g48434.t1/87-111
SPIMU_SM000222S06970/164-188
SPIMU_SM000206S06252/166-190
SPIMU_SM000195S05287/166-190
CHRZO_Cz02g07210.t1/160-184
GONAV_comp55006_c0_seq1_6_ORF10_477/376-400
GUITH_112802/243-267
CHLRE_Cre03.g201250.t1.2/173-197
VOLCA_Vocar.0011s0137.1.p/173-197
AZOFIhc_s2287.g110181/286-305
ORYSAJA_LOC_Os03g05480.1/540-562
CHLAT_Chrsp58S09137/106-130
CHLAT_Chrsp29S04621/133-157
SPIMU_SM000074S21666/275-299
SPIMU_SM000435S15745/245-269
SPIMU_SM000149S01347/244-268
CHABR_g46910.t1/454-478
AZOFIhc_s0061.g035008/228-252
AZOFIhc_s0159.g053974/221-245
PHYPAV33_Pp3c9_15270V3.1/206-230
ANTAG_AagrBONN_evm.model.Sc2ySwM_344.2110.1/517-541
AZOFIhc_s0096.g043711/242-266
PHYPAV33_Pp3c21_1210V3.1/267-291
PHYPAV33_Pp3c18_21380V3.1/261-285
PHYPAV33_Pp3c11_2350V3.1/248-272
PHYPAV33_Pp3c7_24100V3.1/237-261
PHYPAV33_Pp3c19_5900V3.1/262-286
PHYPAV33_Pp3c4_13250V3.1/210-234
MARPO_Mapoly0013s0172.1.p/189-213
GINBI_Gb_16426/215-239
GUITH_61669/1-21
AZOFIhc_s1916.g107972/65-87
GONAV_comp61070_c0_seq3_6_ORF6_294/273-289
PORPU_evm.model.contig_2246.6/513-535
PORPU_evm.model.contig_3601.2/367-389
ANTAG_AagrBONN_evm.model.Sc2ySwM_228.4391.1/306-331
CALTU_IDg9700t1/68-89
CYAME_jgi|CyamerSoos_1|2474|CmerSOOS_G2575.1/481-502
AZOFIhc_s0027.g023479/278-301
MARPO_Mapoly0048s0048.1.p/374-397
KLEFL_kfl00300_0030_v1.1/339-362
AZOFIhc_s1070.g096819/199-221
CALTU_IDg8447t1/196-219
PHYPAV33_Pp3c9_18370V3.1/24-47
AZOFIhc_s0007.g010626/27-50
PORUM_Pum0027s0077.1.p/188-212
PYRYE_py07017.t1/228-252
PYRYE_py09575.t1/534-559
PYRYE_py10257.t1/429-452
PORUM_Pum1432s0002.1.p/366-387
KLEFL_kfl00198_0060_v1.1/264-287
CHOCR_CDF36864.1/416-440
PYRYE_py11171.t1/81-103
CHABR_g10879.t1/610-634
CHLAT_Chrsp108S01363/159-181
PYRYE_py11397.t1/1239-1263
CYACA_ctg_313.g177/261-283
PYRYE_py04491.t1/1040-1063
PORUM_Pum1750s0001.1.p/1024-1047
CHOCR_CDF36227.1/452-473
PYRYE_py08412.t1/577-600
PORUM_Pum0103s0062.1.p/456-479
PORUM_Pum0458s0001.1.p/1268-1290
PYRYE_py09190.t1/480-502
PYRYE_py09607.t1/197-219
PYRYE_py00653.t1/379-402
PORUM_Pum0427s0015.1.p/482-503
GRACH_PXF47706.1/113-136
PORUM_Pum0130s0004.1.p/316-339
ARATH_AT5G04240.1/1252-1275
GINBI_Gb_15927/530-553
GINBI_Gb_39738/1973-1995
AZOFIhc_s0304.g063842/1632-1654
ORYSAJA_LOC_Os01g67970.1/1192-1214
ARATH_AT3G48430.1/1268-1290
ORYSAJA_LOC_Os03g05480.1/565-587
ORYSAJA_LOC_Os03g05690.1/581-603
GUITH_115351/342-362
CHOCR_CDF32272.1/325-346
PORPU_evm.model.contig_2130.9/753-776
PORPU_evm.model.contig_3428.4/498-522
GONAV_comp61894_c0_seq5_6_ORF16_311/139-163
MARPO_Mapoly0061s0023.1.p/233-252
MARPO_Mapoly0029s0056.1.p/635-657
PORUM_Pum0027s0077.1.p/159-182
CHABR_g46910.t1/508-529
CHLAT_Chrsp29S04621/204-225
PHYPAV33_Pp3c9_15270V3.1/262-284
CHLAT_Chrsp58S09137/176-198
SPIMU_SM000074S21666/340-362
SPIMU_SM000435S15745/310-332
SPIMU_SM000149S01347/309-331
AZOFIhc_s0096.g043711/301-323
GINBI_Gb_16426/274-296
PHYPAV33_Pp3c21_1210V3.1/322-344
PHYPAV33_Pp3c7_24100V3.1/294-316
MARPO_Mapoly0013s0172.1.p/247-269
PHYPAV33_Pp3c19_5900V3.1/317-339
PHYPAV33_Pp3c18_21380V3.1/316-338
PHYPAV33_Pp3c11_2350V3.1/303-325
PHYPAV33_Pp3c4_13250V3.1/268-290
AZOFIhc_s0061.g035008/325-347
ANTAG_AagrBONN_evm.model.Sc2ySwM_344.2110.1/576-598
GRACH_PXF40971.1/16-35
SPIMU_SM000007S20819/117-140
SPIMU_SM000004S14991/117-140
CALTU_IDg7988t1/111-133
PORUM_Pum0734s0001.1.p/221-241
AZOFIhc_s0951.g093760/479-496
CALTU_IDg10487t1/30-51
CHRZO_UNPLg00240.t1/97-114
ORYSAJA_LOC_Os06g07020.1/157-180
PYRYE_py04632.t1/129-153
GRACH_PXF42343.1/410-431
GRACH_PXF41579.1/405-426
GRACH_PXF43775.1/227-249
GRACH_PXF40983.1/227-249
GONAV_comp19984_c0_seq1_6_ORF19_591/295-317
CHABR_g40972.t1/914-936
ARATH_AT1G74250.1/601-620
KLEFL_kfl00160_0160_v1.1/646-665
PHYPAV33_Pp3c5_3270V3.1/685-705
MARPO_Mapoly0088s0065.1.p/745-765
GINBI_Gb_36905/709-728
AZOFIhc_s0129.g048868/692-715
CHLRE_Cre05.g240650.t1.2/747-765
CHLAT_Chrsp23S03774/548-569
SPIMU_SM000068S20540/517-540
SPIMU_SM000013S26514/601-624
SPIMU_SM000157S02067/845-868
CHRZO_Cz14g02220.t1/830-852
VOLCA_Vocar.0003s0383.1.p/828-850
PENMA_pm004120g0030/686-709
OSTTA_jgi|Ostta1115_2|80655|e_gw1.88.941.1/5-24
GONAV_comp60766_c1_seq1_6_ORF18_949/124-142
GUITH_103863/12-33
CHOCR_CDF32272.1/293-317
PORPU_evm.model.contig_3428.4/469-492
CHLVU_genemark_Scaffold_100-abinit-gene-0.9-mRNA-1/534-563
PYRYE_py08282.t1/565-588
PORUM_Pum0954s0003.1.p/507-525
PYRYE_py02863.t1/651-671

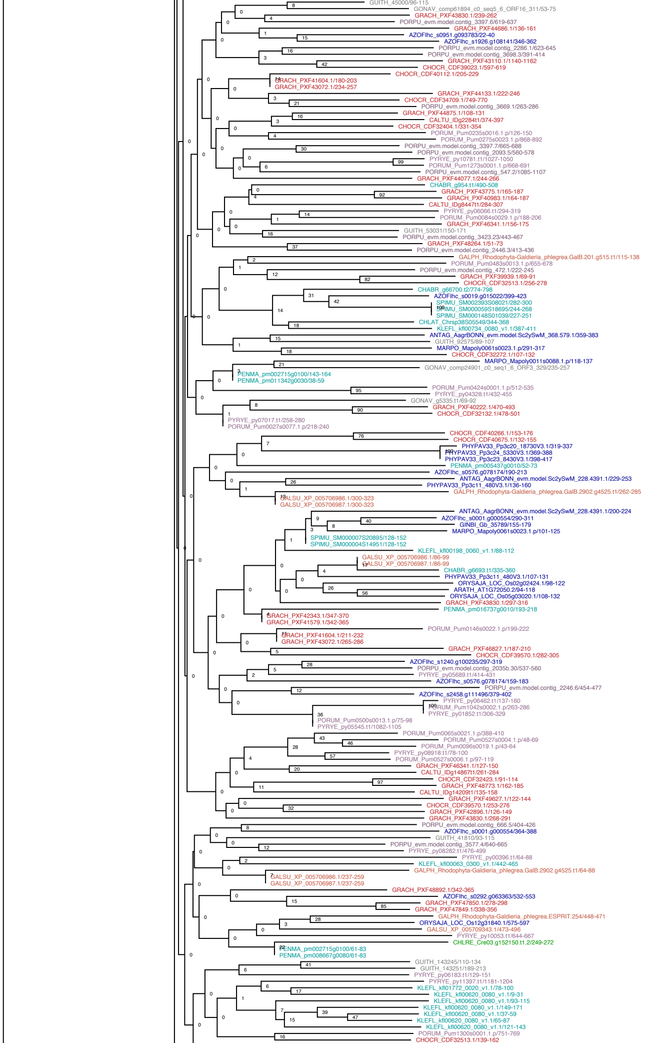

GUITH_45000/96-115
GONAV_comp61894_c0_seq5_6_ORF16_311/53-75
GRACH_PXF43830.1/239-262
PORPU_evm.model.contig_3397.6/619-637
GRACH_PXF44686.1/136-161
AZOFlhc_s0951.g093783/22-40
AZOFlhc_s1926.g108141/346-362
PORPU_evm.model.contig_2286.1/623-645
PORPU_evm.model.contig_3698.3/391-414
GRACH_PXF43110.1/1140-1162
CHOCR_CDF39023.1/597-619
CHOCR_CDF40112.1/205-229
GRACH_PXF41604.1/180-203
GRACH_PXF43072.1/234-257
GRACH_PXF44133.1/222-246
CHOCR_CDF34709.1/749-770
PORPU_evm.model.contig_3669.1/263-286
GRACH_PXF44875.1/108-131
CALTU_IDg2284t1/374-397
CHOCR_CDF32404.1/331-354
PORUM_Pum0235s0016.1.p/126-150
PORUM_Pum0275s0023.1.p/868-892
PORPU_evm.model.contig_3397.7/665-688
PORPU_evm.model.contig_2093.5/560-578
PYRYE_py10781.t1/1027-1050
PORUM_Pum1273s0001.1.p/668-691
PORPU_evm.model.contig_547.2/1085-1107
GRACH_PXF44077.1/244-266
CHABR_g954.t1/490-508
GRACH_PXF43775.1/165-187
GRACH_PXF40983.1/164-187
CALTU_IDg8447t1/284-307
PYRYE_py06066.t1/294-319
PORUM_Pum0084s0029.1.p/188-206
GRACH_PXF46341.1/156-175
GUITH_53031/150-171
PORPU_evm.model.contig_3423.23/443-467
GRACH_PXF48264.1/51-73
PORPU_evm.model.contig_2446.3/413-436
GALPH_Rhodophyta-Galdieria_phlegrea.GalB.201.g515.t1/115-138
PORUM_Pum0483s0013.1.p/655-678
PORPU_evm.model.contig_472.1/222-245
GRACH_PXF39939.1/69-91
CHOCR_CDF32513.1/256-278
CHABR_g66700.t2/774-798
AZOFlhc_s0019.g015022/399-423
SPIMU_SM002393S08021/282-300
SPIMU_SM000059S18695/244-268
SPIMU_SM000148S01039/227-251
CHLAT_Chrsp38S05549/344-368
KLEFL_kfl00734_0080_v1.1/387-411
ANTAG_AagrBONN_evm.model.Sc2ySwM_368.579.1/359-383
GUITH_92575/89-107
MARPO_Mapoly0061s0023.1.p/291-317
CHOCR_CDF32272.1/107-132
MARPO_Mapoly0011s0088.1.p/118-137
GONAV_comp24901_c0_seq1_6_ORF3_329/235-257
PENMA_pm002715g0100/143-164
PENMA_pm011342g0030/38-59
PORUM_Pum0424s0001.1.p/512-535
PYRYE_py04328.t1/432-455
GONAV_g5335.t1/69-92
GRACH_PXF40222.1/470-493
CHOCR_CDF32132.1/478-501
PYRYE_py07017.t1/258-280
PORUM_Pum0027s0077.1.p/218-240
CHOCR_CDF40266.1/153-176
CHOCR_CDF40675.1/132-155
PHYPAV33_Pp3c20_18730V3.1/319-337
PHYPAV33_Pp3c24_5330V3.1/369-388
PHYPAV33_Pp3c23_8430V3.1/398-417
PENMA_pm005437g0010/52-73
AZOFlhc_s0576.g078174/190-213
ANTAG_AagrBONN_evm.model.Sc2ySwM_228.4391.1/229-253
PHYPAV33_Pp3c11_480V3.1/136-160
GALPH_Rhodophyta-Galdieria_phlegrea.GalB.2902.g4525.t1/262-285
GALSU_XP_005706986.1/300-323
GALSU_XP_005706987.1/300-323
ANTAG_AagrBONN_evm.model.Sc2ySwM_228.4391.1/200-224
AZOFlhc_s0001.g000554/290-311
GINBI_Gb_35789/155-179
MARPO_Mapoly0061s0023.1.p/101-125
SPIMU_SM000007S20895/128-152
SPIMU_SM000004S14951/128-152
KLEFL_kfl00198_0060_v1.1/88-112
GALSU_XP_005706986.1/86-99
GALSU_XP_005706987.1/86-99
CHABR_g6693.t1/335-360
PHYPAV33_Pp3c11_480V3.1/107-131
ORYSAJA_LOC_Os02g02424.1/98-122
ARATH_AT1G72050.2/94-118
ORYSAJA_LOC_Os05g03020.1/108-132
GRACH_PXF43830.1/297-316
PENMA_pm016737g0010/193-218
GRACH_PXF42343.1/347-370
GRACH_PXF41579.1/342-365
PORUM_Pum0146s0022.1.p/199-222
GRACH_PXF41604.1/211-232
GRACH_PXF43072.1/265-286
GRACH_PXF46827.1/187-210
CHOCR_CDF39570.1/282-305
AZOFlhc_s1240.g100235/297-319
PORPU_evm.model.contig_2035b.30/537-560
PYRYE_py05689.t1/414-431
AZOFlhc_s0576.g078174/159-183
PORPU_evm.model.contig_2246.6/454-477
AZOFlhc_s2458.g111496/379-402
PYRYE_py06462.t1/137-160
PORUM_Pum1042s0002.1.p/263-286
PYRYE_py01852.t1/306-329
PORUM_Pum0500s0013.1.p/75-98
PYRYE_py05545.t1/1082-1105
PORUM_Pum0065s0021.1.p/388-410
PORUM_Pum0527s0004.1.p/48-69
PORUM_Pum0096s0019.1.p/43-64
PYRYE_py08918.t1/78-100
PORUM_Pum0527s0006.1.p/97-119
GRACH_PXF46341.1/127-150
CALTU_IDg14867t1/261-284
CHOCR_CDF32423.1/91-114
GRACH_PXF48773.1/162-185
CALTU_IDg14209t1/135-158
GRACH_PXF49627.1/122-144
CHOCR_CDF39570.1/253-276
GRACH_PXF42896.1/126-149
GRACH_PXF43830.1/268-291
PORPU_evm.model.contig_666.5/404-426
AZOFlhc_s0001.g000554/364-388
GUITH_41810/93-115
PORPU_evm.model.contig_3577.4/640-665
PYRYE_py08282.t1/476-499
PYRYE_py00396.t1/64-88
KLEFL_kfl00063_0300_v1.1/442-465
GALPH_Rhodophyta-Galdieria_phlegrea.GalB.2902.g4525.t1/64-88
GALSU_XP_005706986.1/237-259
GALSU_XP_005706987.1/237-259
GRACH_PXF48892.1/342-365
AZOFlhc_s0292.g063363/532-553
GRACH_PXF47850.1/278-298
GRACH_PXF47849.1/338-356
GALPH_Rhodophyta-Galdieria_phlegrea.ESPRIT.254/448-471
ORYSAJA_LOC_Os12g31840.1/575-597
GALSU_XP_005709343.1/473-496
PYRYE_py10053.t1/644-667
CHLRE_Cre03.g152150.t1.2/249-272
PENMA_pm002715g0100/61-83
PENMA_pm008667g0080/61-83
GUITH_143245/110-134
GUITH_143251/189-213
PYRYE_py06183.t1/129-151
PYRYE_py11397.t1/1181-1204
KLEFL_kfl01772_0020_v1.1/78-100
KLEFL_kfl00620_0080_v1.1/9-31
KLEFL_kfl00620_0080_v1.1/93-115
KLEFL_kfl00620_0080_v1.1/149-171
KLEFL_kfl00620_0080_v1.1/37-59
KLEFL_kfl00620_0080_v1.1/65-87
KLEFL_kfl00620_0080_v1.1/121-143
PORUM_Pum1300s0001.1.p/751-769
CHOCR_CDF32513.1/139-162

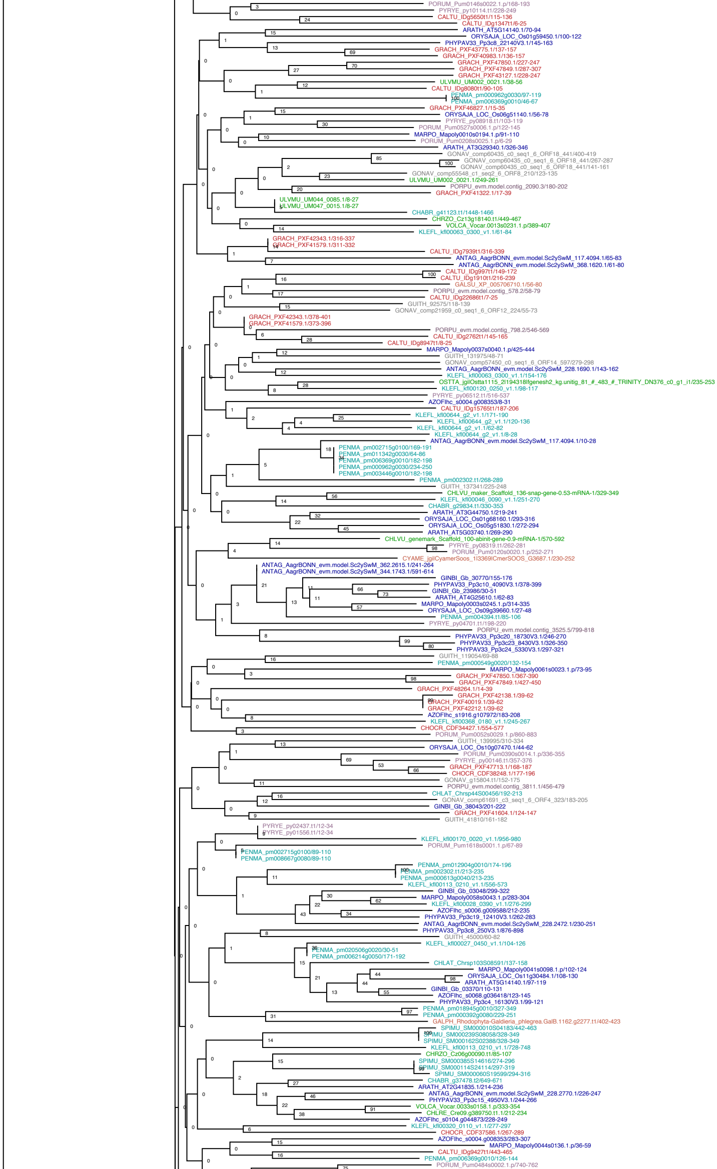
PORUM_Pum0146s0022.1.p/168-193
PYRYE_py10114.t1/228-249
CALTU_IDg5650t1/115-136
CALTU_IDg1347t1/6-25
ARATH_AT5G14140.1/70-94
ORYSAJA_LOC_Os01g59450.1/100-122
PHYPAV33_Pp3c8_22140V3.1/145-163
GRACH_PXF43775.1/137-157
GRACH_PXF40983.1/136-157
GRACH_PXF47850.1/227-247
GRACH_PXF47849.1/287-307
GRACH_PXF43127.1/228-247
ULVMU_UM002_0021.1/38-56
CALTU_IDg8080t1/90-105
PENMA_pm000962g0030/97-119
PENMA_pm006369g0010/46-67
GRACH_PXF46827.1/15-35
ORYSAJA_LOC_Os06g51140.1/56-78
PYRYE_py08918.t1/103-119
PORUM_Pum0527s0006.1.p/122-145
MARPO_Mapoly0010s0194.1.p/91-110
PORUM_Pum0208s0025.1.p/6-29
ARATH_AT3G29340.1/326-346
GONAV_comp60435_c0_seq1_6_ORF18_441/400-419
GONAV_comp60435_c0_seq1_6_ORF18_441/267-287
GONAV_comp60435_c0_seq1_6_ORF18_441/141-161
GONAV_comp55548_c1_seq2_6_ORF8_210/123-135
ULVMU_UM002_0021.1/249-261
PORPU_evm.model.contig_2090.3/180-202
GRACH_PXF41322.1/17-39
ULVMU_UM044_0085.1/8-27
ULVMU_UM047_0015.1/8-27
CHABR_g41123.t1/1448-1466
CHRZO_Cz13g18140.t1/449-467
VOLCA_Vocar.0013s0231.1.p/389-407
KLEFL_kfl00063_0300_v1.1/61-84
GRACH_PXF42343.1/316-337
GRACH_PXF41579.1/311-332
CALTU_IDg7939t1/316-339
ANTAG_AagrBONN_evm.model.Sc2ySwM_117.4094.1/65-83
ANTAG_AagrBONN_evm.model.Sc2ySwM_368.1620.1/61-80
CALTU_IDg997t1/149-172
CALTU_IDg1910t1/216-239
GALSU_XP_005706710.1/56-80
PORPU_evm.model.contig_578.2/58-79
CALTU_IDg22686t1/7-25
GUITH_92575/118-139
GONAV_comp21959_c0_seq1_6_ORF12_224/55-73
GRACH_PXF42343.1/378-401
GRACH_PXF41579.1/373-396
PORPU_evm.model.contig_798.2/546-569
CALTU_IDg2762t1/145-165
CALTU_IDg8947t1/8-25
MARPO_Mapoly0037s0040.1.p/425-444
GUITH_131975/48-71
GONAV_comp57450_c0_seq1_6_ORF14_597/279-298
ANTAG_AagrBONN_evm.model.Sc2ySwM_228.1690.1/143-162
KLEFL_kfl00063_0300_v1.1/154-176
OSTTA_jgi|Ostta1115_2|194318|fgenesh2_kg.unitig_81_#_483_#_TRINITY_DN376_c0_g1_i1/235-253
KLEFL_kfl00120_0250_v1.1/98-117
PYRYE_py06512.t1/516-537
AZOFIhc_s0004.g008353/8-31
CALTU_IDg15765t1/187-206
KLEFL_kfl00644_g2_v1.1/171-190
KLEFL_kfl00644_g2_v1.1/120-136
KLEFL_kfl00644_g2_v1.1/62-82
KLEFL_kfl00644_g2_v1.1/8-28
ANTAG_AagrBONN_evm.model.Sc2ySwM_117.4094.1/10-28
PENMA_pm002715g0100/169-191
PENMA_pm011342g0030/64-86
PENMA_pm006369g0010/182-198
PENMA_pm000962g0030/234-250
PENMA_pm003446g0010/182-198
PENMA_pm002302.t1/268-289
GUITH_137341/225-248
CHLVU_maker_Scaffold_136-snap-gene-0.53-mRNA-1/329-349
KLEFL_kfl00046_0090_v1.1/251-270
CHABR_g29834.t1/330-353
ARATH_AT3G44750.1/219-241
ORYSAJA_LOC_Os01g68160.1/293-316
ORYSAJA_LOC_Os05g51830.1/272-294
ARATH_AT5G03740.1/269-290
CHLVU_genemark_Scaffold_100-abinit-gene-0.9-mRNA-1/570-592
PYRYE_py08319.t1/262-281
PORUM_Pum0120s0020.1.p/252-271
CYAME_jgi|CyamerSoos_1|3369|CmerSOOS_G3687.1/230-252
ANTAG_AagrBONN_evm.model.Sc2ySwM_362.2615.1/241-264
ANTAG_AagrBONN_evm.model.Sc2ySwM_344.1743.1/591-614
GINBI_Gb_30770/155-176
PHYPAV33_Pp3c10_4090V3.1/378-399
GINBI_Gb_23986/30-51
ARATH_AT4G25610.1/62-83
MARPO_Mapoly0003s0245.1.p/314-335
ORYSAJA_LOC_Os09g39660.1/27-48
PENMA_pm004394.t1/85-106
PYRYE_py04701.t1/198-220
PORPU_evm.model.contig_3525.5/799-818
PHYPAV33_Pp3c20_18730V3.1/246-270
PHYPAV33_Pp3c23_8430V3.1/326-350
PHYPAV33_Pp3c24_5330V3.1/297-321
GUITH_119054/69-88
PENMA_pm000549g0020/132-154
MARPO_Mapoly0061s0023.1.p/73-95
GRACH_PXF47850.1/367-390
GRACH_PXF47849.1/427-450
GRACH_PXF48264.1/14-39
GRACH_PXF42138.1/39-62
GRACH_PXF40019.1/39-62
GRACH_PXF42212.1/39-62
AZOFIhc_s1916.g107972/183-208
KLEFL_kfl00368_0180_v1.1/245-267
CHOCR_CDF34427.1/554-577
PORUM_Pum0052s0029.1.p/860-883
GUITH_139995/310-334
ORYSAJA_LOC_Os10g07470.1/44-62
PORUM_Pum0390s0014.1.p/336-355
PYRYE_py00146.t1/357-376
GRACH_PXF47713.1/168-187
CHOCR_CDF38248.1/177-196
GONAV_g15804.t1/152-175
PORPU_evm.model.contig_3811.1/456-479
CHLAT_Chrsp44S00456/192-213
GONAV_comp61691_c3_seq1_6_ORF4_323/183-205
GINBI_Gb_38043/201-222
GRACH_PXF41604.1/124-147
GUITH_41810/161-182
PYRYE_py02437.t1/12-34
PYRYE_py01556.t1/12-34
KLEFL_kfl00170_0020_v1.1/956-980
PORUM_Pum1618s0001.1.p/67-89
PENMA_pm002715g0100/89-110
PENMA_pm008667g0080/89-110
PENMA_pm012904g0010/174-196
PENMA_pm002302.t1/213-235
PENMA_pm000613g0040/213-235
KLEFL_kfl00113_0210_v1.1/556-573
GINBI_Gb_03048/299-322
MARPO_Mapoly0058s0043.1.p/283-304
KLEFL_kfl00028_0390_v1.1/276-299
AZOFIhc_s0006.g009588/212-235
PHYPAV33_Pp3c19_12410V3.1/262-283
ANTAG_AagrBONN_evm.model.Sc2ySwM_228.2472.1/230-251
PHYPAV33_Pp3c8_250V3.1/876-898
GUITH_45000/60-82
KLEFL_kfl00027_0450_v1.1/104-126
PENMA_pm020506g0020/30-51
PENMA_pm006214g0050/171-192
CHLAT_Chrsp103S08591/137-158
MARPO_Mapoly0041s0098.1.p/102-124
ORYSAJA_LOC_Os11g30484.1/108-130
ARATH_AT5G14140.1/97-119
GINBI_Gb_03370/110-131
AZOFIhc_s0068.g036418/123-145
PHYPAV33_Pp3c4_16130V3.1/99-121
PENMA_pm018945g0010/327-349
PENMA_pm000392g0080/229-251
GALPH_Rhodophyta-Galdieria_phlegrea.GalB.1162.g2277.t1/402-423
SPIMU_SM000010S04183/442-463
SPIMU_SM000239S08058/328-349
SPIMU_SM000162S02388/328-349
KLEFL_kfl00113_0210_v1.1/728-748
CHRZO_Cz06g00090.t1/85-107
SPIMU_SM000385S14616/274-296
SPIMU_SM000114S24114/297-319
SPIMU_SM000060S19599/294-316
CHABR_g37478.t2/649-671
ARATH_AT2G41835.1/214-236
ANTAG_AagrBONN_evm.model.Sc2ySwM_228.2770.1/226-247
PHYPAV33_Pp3c15_4950V3.1/244-266
VOLCA_Vocar.0033s0158.1.p/333-354
CHLRE_Cre09.g389750.t1.1/212-234
AZOFIhc_s0104.g044873/228-249
KLEFL_kfl00320_0110_v1.1/277-297
CHOCR_CDF37586.1/267-289
AZOFIhc_s0004.g008353/283-307
MARPO_Mapoly0044s0136.1.p/36-59
CALTU_IDg9427t1/443-465
PENMA_pm006369g0010/126-144
PORUM_Pum0484s0002.1.p/740-762

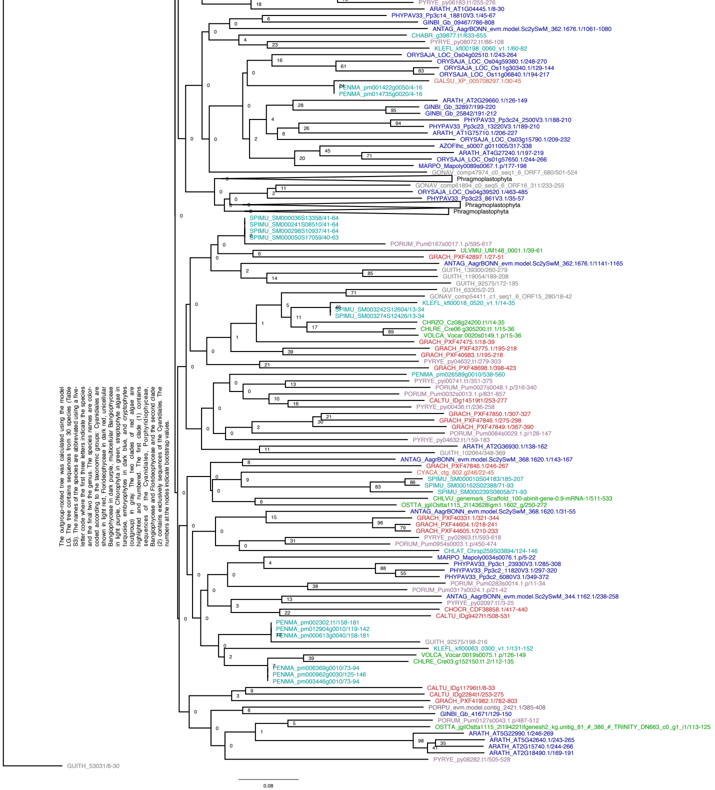

The outgroup-rooted tree was calculated using the model LG. The tree contains sequences from 30 species (Table S3). The names of the species are abbreviated using a five-letter code where the first three letters indicate the species and the final two the genus. The species names are color-coded according to the taxonomic groups: Cyanidiales are shown in light red, Florideophyceae in dark red, unicellular Bangiophyceae in dark purple, multicellular Bangiophyceae in light purple, Chlorophyta in green, streptophyte algae in turquoise, embryophytes in dark blue, and cryptophytes (outgroup) in gray. The two clades of red algae are highlighted and numbered. The first clade (1) contains sequences of the Cyanidiales, Porphyridiophyceae, Bangiophyceae and Florideophyceae and the second clade (2) contains exclusively sequences of the Cyanidiales. The numbers at the nodes indicate bootstrap values.
