# Supplementary material for: Signatures of Transcription Factor Evolution and the Secondary Gain of Red Algae Complexity
Source: Genes (Basel). 2021 Jul 9;12(7):1055. doi: 10.3390/genes12071055 (PMC8304369; doi:10.3390/genes12071055)
Supplement: Supplementary file 1 [file genes-12-01055-s001.zip › Figure S3+S4.pdf]

Figure S3: Neighbor joining tree of the HSF family.

The outgroup-rooted trees contain sequences from 30 species (Table S1). The names of the species are abbreviated using a five-letter code where the first three letters indicate the species and the final two the genus. The species names are color-coded according to the taxonomic groups: Cyanidiales are shown in light red, Florideophyceae in dark red, Porphyridiophyceae in dark purple, Bangiophyceae in light purple, Chlorophyta in green, streptophyte algae in turquoise, embryophytes in dark blue, and cryptophytes (outgroup) in gray. The numbers at the nodes indicate bootstrap values.

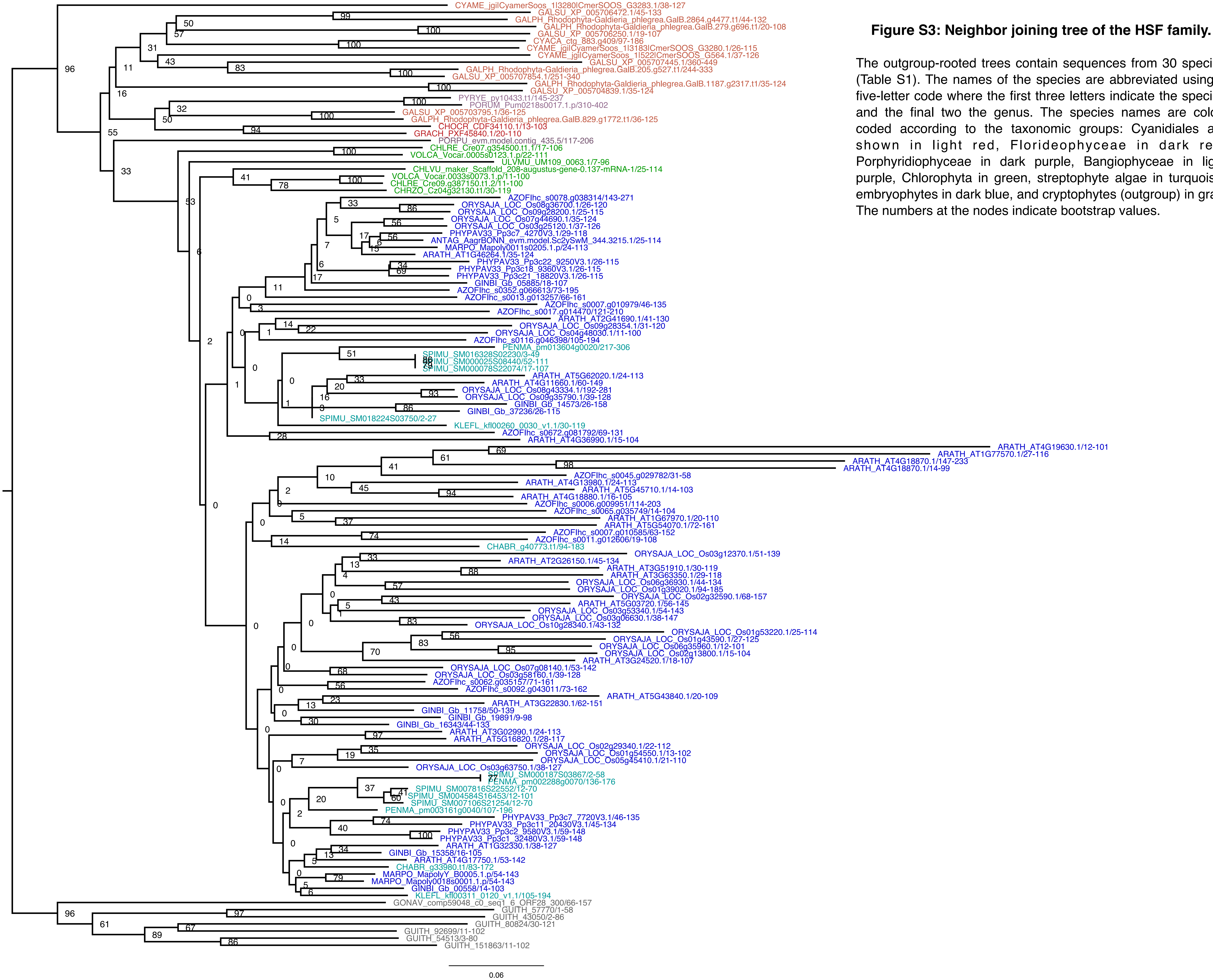

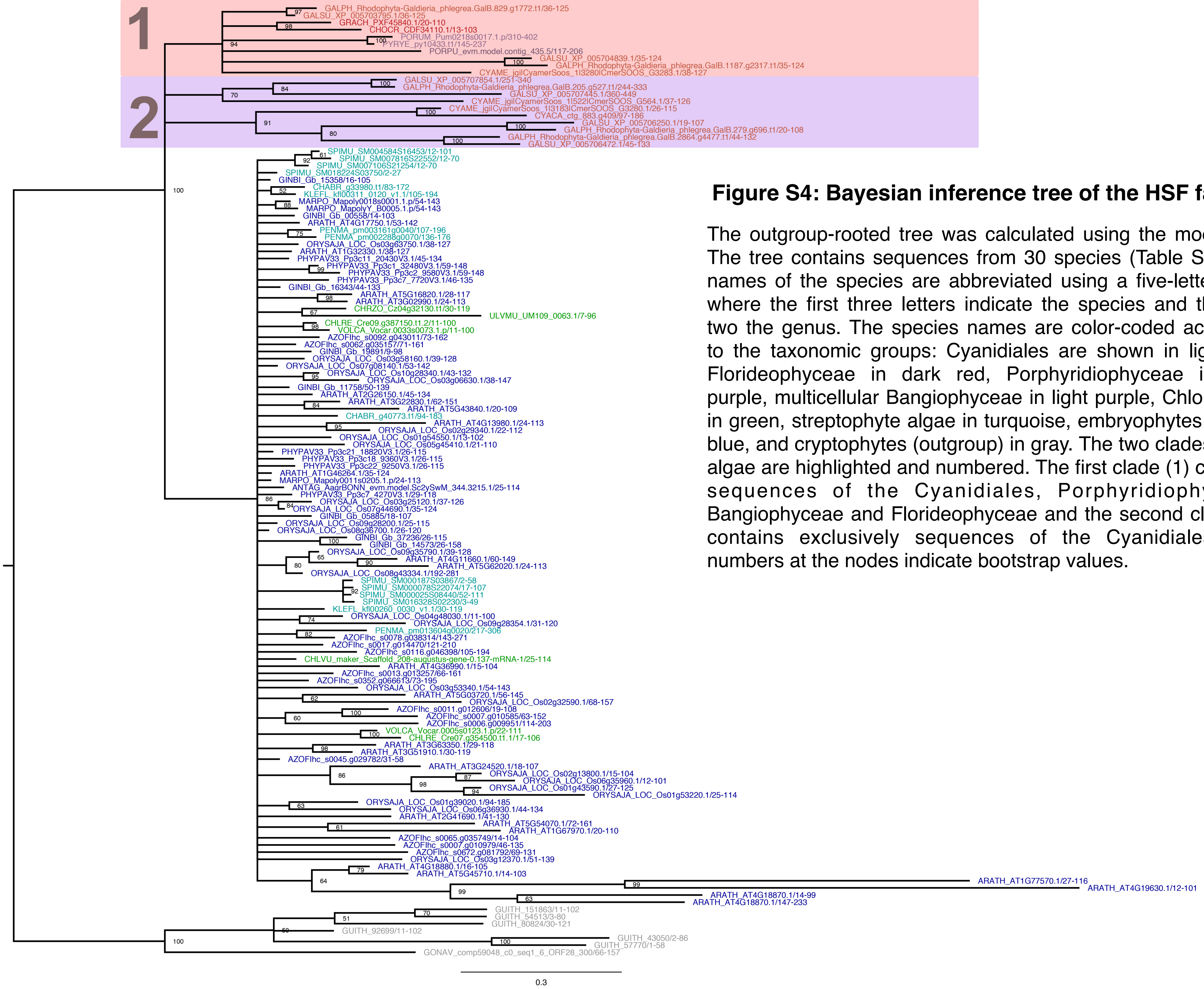

Figure S4: Bayesian inference tree of the HSF family.

The outgroup-rooted tree was calculated using the model LG. The tree contains sequences from 30 species (Table S1). The names of the species are abbreviated using a five-letter code where the first three letters indicate the species and the final two the genus. The species names are color-coded according to the taxonomic groups: Cyanidiales are shown in light red, Florideophyceae in dark red, Porphyridiophyceae in dark purple, multicellular Bangiophyceae in light purple, Chlorophyta in green, streptophyte algae in turquoise, embryophytes in dark blue, and cryptophytes (outgroup) in gray. The two clades of red algae are highlighted and numbered. The first clade (1) contains sequences of the Cyanidiales, Porphyridiophyceae, Bangiophyceae and Florideophyceae and the second clade (2) contains exclusively sequences of the Cyanidiales. The numbers at the nodes indicate bootstrap values.
